# Response of marine benthic fauna to thin-layer capping with activated carbon in a large-scale field experiment in the Grenland fjords, Norway

**DOI:** 10.1007/s11356-017-8851-6

**Published:** 2017-04-18

**Authors:** Göran S Samuelsson, Caroline Raymond, Stefan Agrenius, Morten Schaanning, Gerard Cornelissen, Jonas S Gunnarsson

**Affiliations:** 1 Department of Ecology, Environment and Plant Sciences (DEEP), Stockholm University, 106 91 Stockholm, Sweden; 2 Svensk Ekologikonsult, Vegagatan 3, 113 29 Stockholm, Sweden; 3 Department of Marine Sciences–Kristineberg, University of Gothenburg, 451 78 Fiskebäckskil, Sweden; 4 Norwegian Institute for Water Research (NIVA), 0349 Oslo, Norway; 5 Norwegian Geotechnical Institute (NGI), P.O. Box 3930 Ullevål Stadium, 0806 Oslo, Norway; 6 Faculty of Environmental Sciences and Natural Resource Management, Norwegian University of Life Sciences (NMBU), 1432 Ås, Norway

**Keywords:** Benthic ecology, Macrofauna, Benthic community, Feeding guilds, Contaminated sediment, Remediation

## Abstract

A field experiment with thin-layer capping was conducted in the Grenland fjords, Norway, for remediation in situ of mercury and dioxin-contaminated sediments. Experimental fields at 30 and 95 m depth were capped with (i) powdered activated carbon (AC) mixed with clay (AC+cla`y), (ii) clay, and (iii) crushed limestone. Ecological effects on the benthic community and species-feeding guilds were studied 1 and 14 months after capping, and a total of 158 species were included in the analyses. The results show that clay and limestone had only minor effects on the benthic community, while AC+clay caused severe perturbations. AC+clay reduced the abundance, biomass, and number of species by up to 90% at both 30 and 95 m depth, and few indications of recovery were found during the period of this investigation. The negative effects of AC+clay were observed on a wide range of species with different feeding strategies, although the suspension feeding brittle star *Amphiura filiformis* was particularly affected. Even though activated carbon is effective in reducing sediment-to-water fluxes of dioxins and other organic pollutants, this study shows that capping with powdered AC can lead to substantial disturbances to the benthic community.

**Electronic supplementary material:**

The online version of this article (doi:10.1007/s11356-017-8851-6) contains supplementary material, which is available to authorized users.

## Introduction

Thin-layer capping (1–10 cm) in situ with active sorbents, such as activated carbon (AC), has been proposed as an alternative method to dredging or conventional capping for sediment remediation (Ghosh et al. 2011). The moderate amount of material needed in thin-layer capping makes the method particularly suitable for remediation of large and deep areas. Activated carbon has a strong sorption capacity for hydrophobic organic contaminants (HOCs) (Cornelissen et al. 2005; Grathwohl and Kleineidam 2000; Luthy et al. 1997), and thin-layer capping with AC has proven to be an efficient method to decrease the bioavailability and the sediment-to-water fluxes of HOCs such as PCBs, PAHs, dioxins, and furans (Beckingham and Ghosh 2011; Cho et al. 2009, 2007; Cornelissen et al. 2012, 2011; Josefsson et al. 2012; Kupryianchyk et al. 2013b; Lin et al. 2014; McLeod et al. 2007; Millward et al. 2005; Samuelsson et al. 2015; Zimmerman et al. 2004, 2005).

Thin-layer capping with AC is also suggested to be less harmful to the benthic fauna compared to conventional capping or dredging (Ghosh et al. 2011). Several studies on AC remediation have reported no negative effects on the benthic fauna (Janssen and Beckingham 2013; Rakowska et al. 2012). However, in a review with a compilation of 82 tests performed within a total of 18 species, negative biological effects of AC treatment were recorded in one fifth of the tests (Janssen and Beckingham 2013). The negative effects on single species assays include decrease in growth (Janssen et al. 2012; Kupryianchyk et al. 2011; Macleod et al. 2008; Millward et al. 2005; Nybom et al. 2012, 2015), lipid content (Janssen et al. 2011, 2012; Jonker et al. 2009; Nybom et al. 2012), and survival (Kupryianchyk et al. 2011; McLeod et al. 2008) as well as changes in behavior (Jonker et al. 2009; Nybom et al. 2012, 2015), reproduction (Nybom et al. 2012, 2015), and morphology (Nybom et al. 2015). Further, a few studies on benthic community level exposed to AC also show contradictory results. No negative effects were shown with granular AC in a fresh water benthic community in Grasse River, USA (Beckingham et al. 2013). A benthic community in a fresh water ditch (Veenkampen, The Netherlands) showed an initial perturbation followed by recolonization and recovery 1 year after exposure of fine particle AC (Kupryianchyk et al. 2012). In contrast, a marine benthic community showed a significant decrease in both the number of species and in their respective abundances 1 year after capping in situ with powdered AC in the Trondheim Harbor, Norway (Cornelissen et al. 2011). However, when the powdered AC was mixed with clay before being applied on top of the sediment, the severity of the effects was reduced (Cornelissen et al. 2011). Furthermore, negative effects on abundance and number of species were documented in a mesocosm experiment, where an intact marine benthic community was exposed to thin-layer capping with powdered AC (Näslund et al. 2012).

Since the benthic macrofauna community has a key role in benthic ecological processes, it is essential to understand the effects of AC on benthic macrofauna communities before AC remediation in situ can be recommended as an environmentally sustainable remediation option. This study addresses the ecological effects of thin-layer capping treatments on benthic communities in the up until now largest field experiment in a marine environment (3 × 10,000 and 1 × 40,000 m^2^). The experiment was conducted in the Grenland fjords in SE Norway, where the sediment has been severely contaminated from historic industrial emissions of dioxins, furans, and mercury (Knutzen et al. 2003). Therefore, a sediment remediation project using thin-layer capping has been considered for a large part of the 53 km^2^ fjord area. We studied the effects of thin-layer capping with AC on two benthic communities, one at 30 m and the other at 95 m depth. The active sorbent AC was mixed with sediment clay in order to prevent negative effects from high concentration of AC, as well as to facilitate its placement on the seafloor (Cornelissen et al. 2011). Additionally, we studied the effects of thin-layer capping with two non-active materials, clay and crushed limestone, at 30 m depth. The ecological effects on the benthic macrofauna were measured 1 and 14 months after capping. The capping efficiency on sediment-to-water fluxes have been presented in Cornelissen et al. (2012, 2015). Compared to other studies with thin-layer capping on marine sediment, this field experiment was carried out on a much larger scale and at greater depth. Moreover, the taxonomic resolution with 158 taxa was greater in this study compared to previous investigations. Benthic communities are structured by the prevailing abiotic conditions, and by the dominating species and feeding guilds (Pearson and Rosenberg 1978, 1987). For a better understanding of the ecological and functional implications on the community, each taxon was grouped into one of four feeding guilds (subsurface deposit feeders, deposit feeders, suspension/filter feeders, and carnivores).

## Materials and methods

### The Grenland fjords

**Fig. 1:**
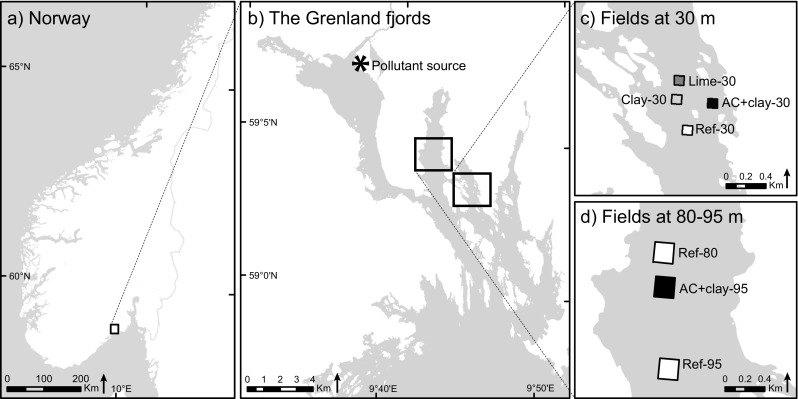
**a** The Grenland fjords are located in south-eastern Norway. **b** The magnesium smelter (pollutant source) was situated in the inner part of the fjord system, nearby the mouth of the Skien River. **c** The experimental fields at 30 m are located in the Ormerfjord, and **d** the fields at 80–95 m depth are located in the Eidangerfjord

The test fields were established at 30 and 80–95 m depth in two branches (Ormerfjord and Eidangerfjord) of the outer Grenland fjords system in SE Norway (Fig. 1, Table 1). The sediments in the fjords are contaminated with persistent organic pollutants such as polychlorinated dibenzo-*p*-dioxins (PCDDs) and dibenzofurans (PCDFs), as well as mercury (Hg) (Knutzen et al. 2003). The contamination originates mainly from a magnesium smelter active from 1951 to 2002, located in the inner part of the fjord system (Fig. 1). The levels of PCDD/F and Hg are three to four times higher at the 80–95 m depth in Eidangerfjord compared to the shallower Ormerfjord (Table 2). The outer fjord area, where the test fields were established, is separated from the inner fjord by a shallow sill at 23 m. The outer fjord is also separated from Skagerrak by a deeper sill at 55 m depth. The inner part of the fjord has a fresh water outflow from the Skien River, resulting in a brackish surface layer (Molvær 1999). Below the brackish layer, the salinity is approximately 30 and increases further to 34.5. The salinity at the sample sites reached 33.4 at 30 m and 33.8–34.5 at 80–95 m depth (Table 2).

**Table 1:** Treatments at the 30 m deep fields in the Ormerfjord and at the 80–95 m deep fields in the Eidangerfjord

Experimental site	Treatment	Abbreviation	Samples unit	Cap thickness (mm)	Field area (m^2^)
30 m depth Ormerfjord	Limestone gravel	Lime-30	Lime-30:1 (*n = 3*) Lime-30:14 (*n* = *5*)	21 ± 12	10,000
	Clay (dredged)	Clay-30	Clay-30:1 (*n* = *3*) Clay-30:14 (*n* = *5*)	37 ± 11	10,000
	Active carbon mixed in clay	AC+clay-30	AC+clay-30:1 (*n* = *3*) AC+clay-30:14 (*n* = *5*)	11 ± 6	10,000
	Reference	Ref-30	Ref-30:1 (*n* = *3*) Ref-30:14 (*n* = *5*)	−	10,000
80–95 m depth Eidangerfjord	Active carbon mixed in clay	AC+clay-95	AC+clay-95:1 (*n* = *3*) AC+clay-95:14 (*n* = *5*)	12 ± 3	40,000
	Reference	Ref-80	Ref-80:1 (*n* = *3*) Ref-80:14 (*n* = *5*)	−	40,000
	Reference	Ref-95	Ref-95:14 (*n* = *5*)	−	40,000

**Table 2:** Sediment characteristics at the experimental fields including total organic carbon (TOC), mercury (Hg), and PCDD/F (TEQ values derived with the 2005 WHO factors), as well as the water oxygen level (O_2_), salinity, and temperature measured in the bottom water during sampling month 1 (September 2009) and month 14 (October 2010)

Field	Depth (m)	Bottom substrate	TOC (%)	Hg (ng/g)	TEQ (ng/kg)	O_2_ (mg/l)month 1 | 14	Salinity month 1 | 14	Temp. (°C) month 1 | 14
Lime-30	29–31	Silty clay	1.6 ± 0.4	255	352	6.0 | 6.3	33.5 | 33.4	13.8 | 12.1
Clay-30	29–30	Silty clay	1.6 ± 0.4	243	344	6.3 | 5.9	33.4 | 33.4	14.2 | 12.2
AC+clay-30	25–29	Silty-muddy clay	1.5 ± 0.3	243	182	6.4 | 7.3	33.2 | 33.3	14.9 | 12.2
Ref-30	29–30	Silty-muddy clay	1.4 ± 0.1	275	346	6.0 | 6.5	33.6 | 33.4	13.9 | 12.2
AC+clay-95	93–95	Silty-clayey mud	2.5 ± 0.7	875	1019	6.7 | 5.1	34.0 | 34.5	12.0 | 7.5
Ref-80	79–83	Silty-clayey mud	2.2 ± 0.4	951	1278	6.4 | 5.4	34.1 | 34.5	10.7 | 7.6
Ref-95	96–98	Silty-clayey mud	–^a^	–^a^	–^a^	– | 5.4	– | 34.5	– | 7.5

^a^The additional reference field Ref-95 was not represented in the TOC, Hg, and PCDD/F analyses since this field was added to the experimental design after these measurements

The bottom environment at 30 m depth in the Ormerfjord can be characterized as a transport bottom, where the sediment consists of silty clay with a mean total organic carbon (TOC) content of 1.4–1.6% in the 0–5-cm top sediment (Table 2). At 80–95 m in the Eidangerfjord, the fields were located on an accumulation bottom with silty-clayey mud sediment and a mean TOC of 2.2–2.5% (Table 2). The deeper area receives approximately three times more sedimented material than the shallower area (1–2 mm compared to ca 0.5 mm aged and compacted sediment, annually). Instead of using an experimental design with pre-cap samples, the homogeneity of the benthic conditions and community structures in the experimental areas were confirmed with data from monitoring programs, daygrab samples (0.025 m^2^) and sediment profile imagery (SPI) (Schaanning et al. 2011). At 30 m, the benthic macrofauna community was dominated by the brittle star *Amphiura* *filiformis*, a passive suspension feeder dependent on water current above the sediment surface, together with the filter feeding bivalve *Corbula gibba*. The community was also characterized by large deposit feeding reworkers, e.g., the burrowing sea urchins *Brissopsis lyrifera* and *Echinocardium cordatum*. At 80–95 m, the benthic community was characterized by reworking deposit feeders e.g., the polychaete *Spiophanes kroeyeri* and the subsurface deposit feeding bivalve *Thyasira equalis*.

### Experimental design

At 30 m depth in the Ormerfjord, three test fields (100 × 100 m, i.e., 10,000 m^2^) were capped with three different capping materials: (i) AC mixed in clay (AC+clay-30), (ii) clean marine clay (Clay-30), and (iii) crushed limestone (Lime-30) (Table 1). The fields were compared to an uncapped reference field (Ref-30). AC was tested as an active sorbent, and clay and limestone were tested as non-active alternatives and carrier material. Clay was also used as cap control in order to separate the cap effect from effects from the AC at 30 m depth. The most innovative treatment, AC+clay, was also tested in the deeper Eidangerfjord, where one test field (200 × 200 m, i.e., 40,000 m^2^) at 95 m depth was capped with AC mixed with clay (AC+clay-95). This deeper capped field was compared to an untreated reference field at 80 m depth (Ref-80). An additional reference field at 95 m depth (Ref-95) was introduced after 14 months in order to control that effects on benthic fauna were due to treatment rather than to different depths. In highly species diverse systems as in this experiment, species composition can change naturally with time (dependent on natural variations in environmental factors and opportunity for e.g., spawning). Therefore, it is essential that the treated fields were compared to similar untreated reference fields sampled at the same time, although comparison to pre-capping condition also would have been valuable. One month after capping, three replicate grabs were sampled from each field (Table 1). Fourteen months after capping, the sampling was extended to five replicate grabs per field in order to better account for the variation connected to the inherent patchiness in species distribution, and to improve the statistical power. The capping was performed in September 2009 and benthic samples were taken 13–14 October 2009 and 8–9 November 2010 i.e., 1 and 14 months after capping.

### Capping materials and establishment of the fields

Limestone obtained from a nearby quarry (NOAH, Langøya) was crushed and sieved to obtain a limestone material ranging from silt to fine gravel (0–5 mm; TOC 0.1%). Clay was suction-dredged at 10 m depth in the inner part of the Ormerfjord, where the top 10 cm of the sediment was discarded to obtain a clean clay sediment (TOC 1.8%; <1 ng kg^−1^ TEQ) (Cornelissen et al. 2012). For the AC+clay treatment, powdered AC (Jacobi Carbons, BP2 fine powder; average particle size of 20 μm; 80% smaller than 45 μm; TOC 76%) was mixed in a 1:10 dw/dw ratio with clay (the same as in the Clay-30) in a large tank on the ship. Salt was added in order to increase the density of the activated carbon, making the slurry salinity to reach 40. During placement, however, the cap would rapidly have been diluted to a salinity that the organisms are adapted to. The capping materials were pumped out 5 to 10 m above the sediment surface. The target cap thickness was 5 cm; however, the clay did not absorb water to the calculated extent. A thinner layer of AC+clay was accepted since the main objective was the active sorbent AC rather than the thickness of the cap. The achieved thickness of the caps were after 1 month measured to 11 ± 6 and 12 ± 3 mm for AC+clay-30 and AC+clay-95 respectively, 37 ± 11 mm for Clay-30, and 21 ± 12 mm for Lime-30 (Eek et al. 2011). After 9 months, the final AC concentration was 2% by dry weight of sediment measured in the AC-treated fields (Cornelissen et al. 2012). A more detailed description of how the capping materials were applied and their effects on reducing contaminant fluxes are presented in Cornelissen et al. (2012, 2015).

### Sampling and analyses of benthic fauna

Benthic macrofauna was sampled with a van Veen grab (0.1 m^2^), and only grabs with the full volume of 19 l were accepted. The samples were sieved through a 1-mm mesh, and the retained material was conserved in 4% buffered formaldehyde. All specimens were with few exceptions identified to species level. Species within the groups Nemertea and Turbellaria were identified only as groups. The sampling in field and the following sample processing and taxon determination in the laboratory followed the European standard (EN ISO 16665: 2014). Abundance (number of individuals per 0.1 m^2^) and biomass (g wet weight per 0.1 m^2^) were determined for each taxon; see supplementary material Table S1 for a complete list of all taxa. All taxa were also classified into functional groups based on their primary feeding strategies: subsurface deposit feeders, (surface) deposit feeders, suspension/filter feeders, and carnivores (predators and scavengers). The classification of feeding strategy is based on literature and expertise knowledge, and a complete list for all taxa is presented in supplementary material Table S2.

### Data analysis

The two experimental areas (Ormerfjord at 30 m and Eidangerfjord at 80–95 m) were treated separately in all statistical analyses. Differences among capping treatments were analyzed using permutational analysis of variance (PERMANOVA) (Anderson 2001) with PRIMER 6+ PERMANOVA statistical software package (Plymouth Laboratories, UK). Benthic community structure was analyzed with multivariate statistics using Bray-Curtis dissimilarity index after fourth-root transformation. The data were also tested for differences in dispersion using the PERM-DISP routine in the PERMANOVA software, since differences in dispersion can influence the result as well as being a measurement of disturbance in a community (Warwick and Clarke 1993). From the multivariate matrix, cluster analysis and non-metric multidimensional scaling plots (n-MDS) were created to visualize relative similarities and dissimilarities between the benthic communities. Similarity analysis was performed on the community structure using the similarity percentage method SIMPER, where similarity between groups is compared at the species level.

Univariate metrics i.e., abundance, number of species, and total biomass were also analyzed using PERMANOVA. Euclidian distance was used for the univariate variables, which were left untransformed unless transformation was needed to achieve homogenous variances.

Complementary post hoc pairwise tests were carried out using the same PERMANOVA procedures (equivalent to Dunnett’s post hoc test in a traditional ANOVA), and Monte Carlo sampling was used when the numbers of unique permutations were low. The significance level for all statistical tests was set at α = 0.05. Significant differences between capping treatments and the references are generally considered as effects by the treatment in the analyses.

Non-statistical analyses included differences in average wet weight per individual. The mean individual weight of each species was compared between the AC+clay and the reference (and clay) fields, and the number of species with increased or decreased mean individual weight was summed and compared between the fields. This was only possible for species co-occurring in the AC+clay and the reference (and clay) fields at the same sampling occasion.

## Results

A total of 4437 specimens from 158 different species (or taxa) were included in the analyses, with 1253 specimens belonging to the 116 species obtained from the 18 van Veen grab samples collected 1 month after capping, and 3184 specimens belonging to the 123 species obtained from the 35 van Veen grab samples collected 14 months after capping. In the fields at 30 m depth, 103 species were found while 112 species were found in the fields at 80–95 m depth.

**Fig. 2:**
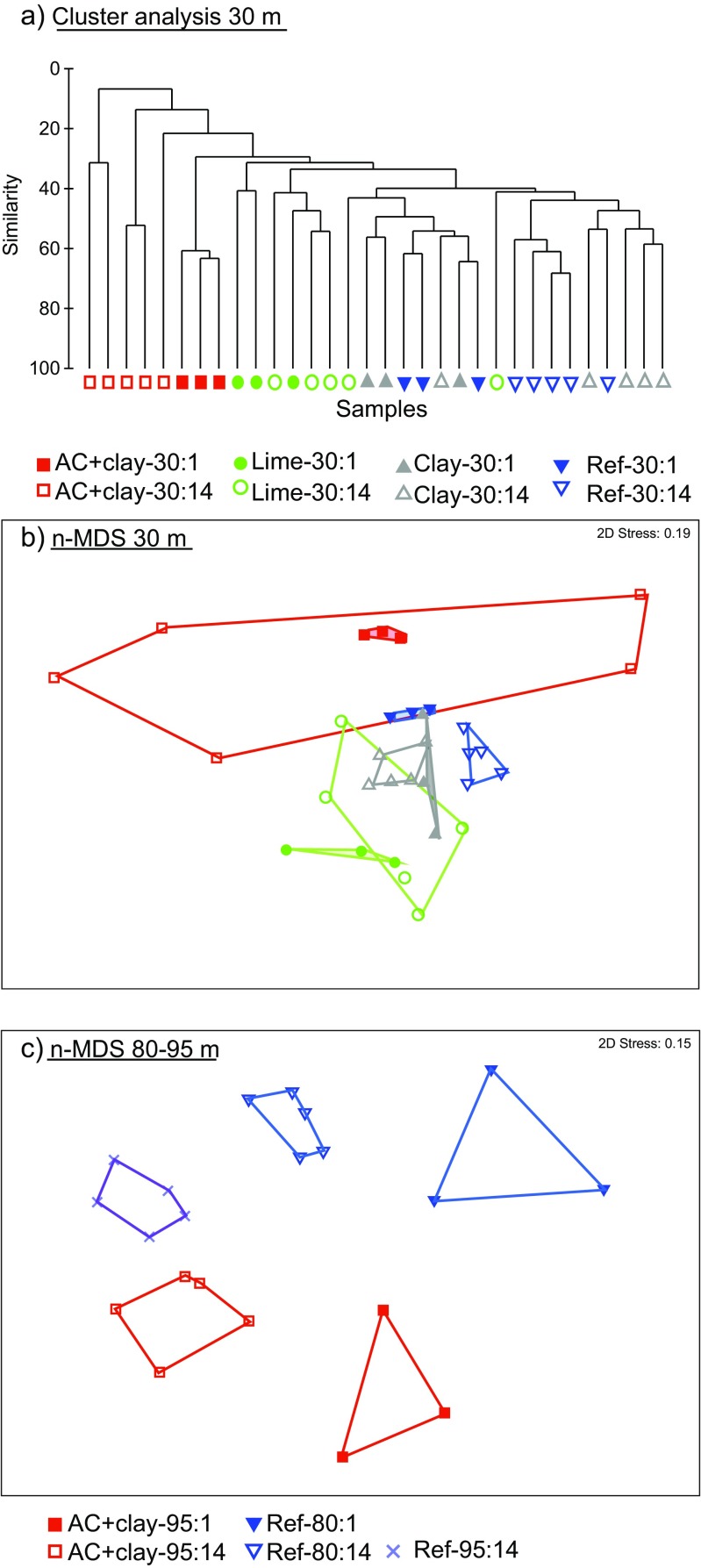
Similarities and dissimilarities in the macrobenthic community in **a** dendrogram for hierarchical cluster analysis at 30 m depth (group average linking), **b** ordination plot (n-MDS) at 30 m depth, and **c** ordination plot (n-MDS) at 80–95 m depth

The multivariate analyses of benthic community structure revealed significant interactions between time and treatment in both fjords (PERMANOVA, see Table 3 for *p*-values). The significant interactions imply that the structure of the benthic communities in the different treatments had changed in dissimilar ways between the two sampling occasions 1 and 14 months after capping. In the 30-m-deep fields, differences in the benthic community structure were essentially driven by reduced abundance and number of species in the AC+clay-30 treatment between 1 and 14 months, compared to stable (or even improved) conditions for the Ref-30, Clay-30, and Lime-30 fields. The differences between the treatments and how these differences change with time are illustrated in the cluster analysis (Fig. 2a), which shows that the community in AC+clay-30 after 14 months is only 7–21% similar to (i.e., 79–93% dissimilar from) the other communities. At 80–95 m depth, the significant interaction was due to differences in development among species. Besides, there were a smaller subset of overlapping species between AC+clay-95 and Ref-80 (37–41%) compared to the larger overlap of species between Ref-80 and Ref-95 (56%).

**Table 3:** Results from PERMANOVA analyses. Compilation of relevant *p*-values, for all statistical analyses of three univariate community metrics (number of species, organism abundance, and total biomass) and the multivariate benthic community structure. Significant *p*-values are shown in bold numbers, α = 0.05. *Df* degrees of freedom, *num* numerator, *den* denominator, *PsF* pseudo-*F* value, *t t*-value, *P*(*perm*) *p*-value by permutation, *P*(*MC*) *p*-value from Monte Carlo sampling

a. Two-factor PERMANOVA analyses											
Depth	Factor		*Df* (num, den)	Number of species		Abundance		Biomass		Community structure	
				*PsF*	P(perm)	*PsF*	P(perm)	*PsF*	P(perm)	*PsF*	P(perm)
30 m	Month (*n* = 2)		1, 24	*6.45*	**0.019**	*0.10*	0.748	*1.11*	0.305	*3.24*	**0.001**
	Treatment (*n* = 4)		3, 24	*14.5*	**0.001**	*27.5*	**0.001**	*3.78*	**0.023**	*3.46*	**0.001**
	Month × Treatment		3, 24	*7.80*	**0.002**	*5.69*	**0.005**	*3.64*	**0.028**	*2.16*	**0.001**
80–95 m	Month (*n* = 2)		1, 16	*1.42*	0.245	*2.49*	0.130	*1.54*	0.875	*4.75*	**0.001**
	Treatment (*n* = 3)		2, 16	*31.8*	**0.001**	*5.99*	**0.011**	*8.88*	**0.003**	*4.03*	**0.001**
	Month × Treatment		1, 16	*0.44*	0.515	*0.09*	0.759	*1.54*	0.229	*2.43*	**0.010**
b. Pairwise comparisons^b^											
Depth	Pairwise comparison		*Df* (num, den)	Number of species		Abundance		Biomass		Community structure	
				*t*	P(perm)	*t*	P(perm)	*t*	P(perm)	*t*	P(perm)
30 m	**Lime-30** vs **Ref-30**		1, 12	*1.13*	0.303	*2.03*	0.173	*1.97*	0.190	*4.11*	**0.001**
	**Clay-30** vs **Ref-30**		1, 12	*2.10*	0.168	*11.2*	**0.007**	*2.03*	0.180	*2.12*	0,231
	**AC+clay-30** vs **Clay-30**		1, 12	*34.4*	**0.001**	*150*	**0.001**	*7.97*	**0.018**	*3.74*	**0.001**
	**AC+clay-30** vs **Ref-30**		1, 12	*38.6*	**0.001**	*48.2*	**0.001**	*2.69*	0.123	*3.66*	**0.001**
80–95 m	**AC+clay-95** vs **Ref-80**		1, 12	*56.1*	**0.001**	*8.46*	**0.015**	*22.1*	**0.001**	*2.31*	**0.001**
	**AC+clay-95** vs **Ref-95**		1, 10	*6.37*	**0.034**	*7.15*	**0.028**	*0.41*	0.532	*1.51*	**0.046**
	**Ref-80** vs **Ref-95**		1, 10	*3.64*	**0.005**	*0.04*	0.838	*3.00*	0.116	*2.05*	**0.006**
c. Post hoc pairwise tests (one-factorial)											
Depth	Pairwise comparison		*Df* (num, den)	Number of species		Abundance		Biomass		Community structure	
				*t*	P(MC)	*t*	P(MC)	*t*	P(MC)	*t*	P(MC)
30 m	**Lime-30** vs **Ref-30**	1 month	1, 4	*0.86*	0.444	*0.32*	0.767	*2.84*	**0.046**	*1.89*	**0.040**
	**Lime-30** vs **Ref-30**	14 months	1, 8	*0.60*	0.562	*2.05*	0.077	*0.34*	0.742	*1.90*	**0.013**
	**Clay-30** vs **Ref-30**	1 month	1, 4	*1.93*	0.119	*4.53*	**0.010**	*1.02*	0.362	*1.23*	0.243
	**Clay-30** vs **Ref-30**	14 months	1, 8	*0.06*	0.952	*1.55*	0.159	*1.25*	0.247	*1.69*	**0.025**
	**AC+clay-30** vs **Ref-30**	1 month	1, 4	*0.20*	0.843	*2.82*	**0.047**	*0.06*	0.959	*2.24*	**0.020**
	**AC+clay-30** vs **Ref-30**	14 months	1, 8	*9.65*	**0.001**	*7.78*	**0.001**	*2.34*	**0.048**	*1.86*	**0.018**
	**AC+clay-30** vs **Clay-30**	1 month	1, 4	*1.72*	0.160	*7.62*	**0.002**	*0.89*	0.426	*2.41*	**0.016**
	**AC+clay-30** vs **Clay-30**	14 months	1, 8	*7.26*	**0.001**	*11.5*	**0.001**	*3.40*	**0.009**	*1.64*	**0.043**
	1 vs 14 months	**AC+clay-30**	1, 6	*5.59*	**0.002**	*4.17*	**0.007**	*2.70*	**0.035**	*1.55*	0.079
	1 vs 14 months	**Clay-30**	1, 6	*0.84*	0.434	*0.75*	0.485	*0.27*	0.802	*1.38*	0.113
	1 vs 14 months	**Lime-30**	1, 6	*0.20*	0.848	*1.46*	0.197	*1.82*	0.121	*1.34*	0.129
	1 vs 14 months	**Ref-30**	1, 6	*1.94*	0.099	*0.94*	0.382	*0.78*	0.458	*2.10*	**0.011**
80–95 m	**AC+clay-95** vs **Ref-80**	1 month	1, 4	*3.98*	**0.018**	*5.01*	**0.007**	*7.32*	**0.002**	*1.51*	0.102
	**AC+clay-95** vs **Ref-80**	14 months	1, 8	*6.85*	**0.001**	*2.17*	0.064	*2.31*	**0.046**	*2.46*	**0.002**
	**AC+clay-95** vs **Ref-95**	14 months	1, 8	*3.19*	**0.011**	*2.39*	**0.044**	*0.58*	0.575	*1.62*	**0.034**
	**Ref-80** vs **Ref-95**	14 months	1, 8	*3.94*	**0.004**	*0.19*	0.853	*1.58*	0.149	*2.22*	**0.002**
	1 vs 14 months	**AC+clay-95**	1, 6	*0.36*	0.734	*0.78*	0.471	*0.94*	0.380	*1.86*	**0.022**
	1 vs 14 months	**Ref-80**	1, 6	*1.20*	0.282	*1.53*	0.181	*1.13*	0.301	*1.82*	**0.022**

^a^Ref-95 not included in the interaction analysis since this field was introduced after 14 months

^b^Pairwise results derived from a planned contrast design

### Reference fields

**Fig. 3:**
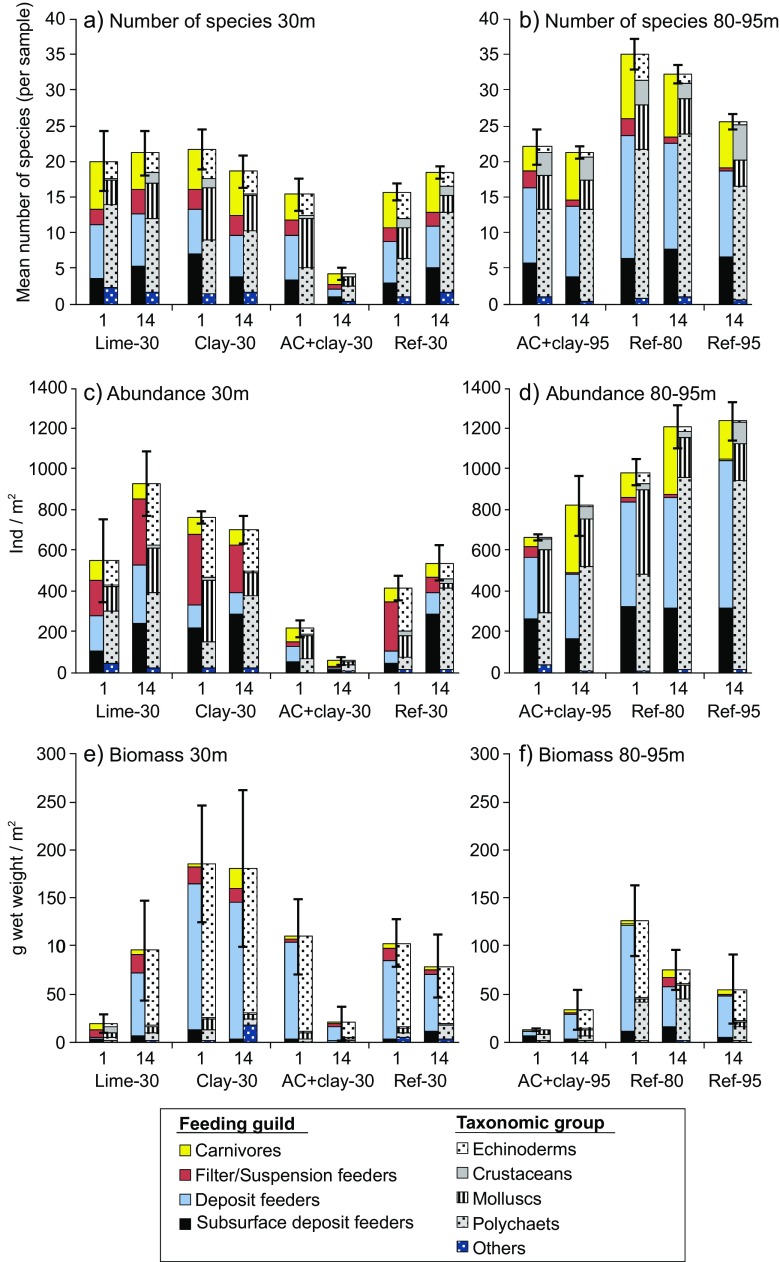
Organisms sorted in feeding guilds and taxonomic groups in: **a** number of species per sample at 30 m, **b** number of species per sample at 80–95 m, **c** organism abundance per square meter at 30 m, **d** organism abundance per square meter at 80–95 m, **e** biomass (g wet weight) per square meter at 30 m, **f** biomass (g wet weight) per square meter at 80–95 m. Mean ± SE, month 1 *n* = 3, month 14 *n* = 5

Ref-30 had no significant differences over time in number of species, organism abundance, or total biomass (Fig. 3, Table 3). The benthic community in Ref-30 was dominated by the abundant suspension feeding brittle star *A. filiformis*. After 1 month, the filter feeding bivalve *C. gibba* was the second most abundant species. After 14 months, there had also been a very strong recruitment of the subsurface deposit feeding polychaete *Scalibregma inflatum*. These three species constituted more than half of the total abundance (Table S1 Species list and Table S3 SIMPER). In biomass, the deposit feeding sea urchins *B. lyrifera* and *E. cordatum* were the dominant species together with *A. filiformis*.

In the 80–95 m deep area, there was a significant difference in community structure between the Ref-95 and the Ref-80 reference fields (Fig. 3). The two reference fields differed in number of species, but not in total abundance or biomass (Table 3). The dissimilarity in species composition between Ref-80 and the deeper Ref-95 was 46% (Table S4 SIMPER), and was mainly due to fluctuations in intermediary abundant species rather than in the dominant species. The top 12 species in the SIMPER analysis contributed to 36% of the dissimilarities, but only constituted 9–16% of the total abundance. The six most abundant species, i.e., the subsurface feeding bivalve *T. equalis* and the polychaete *Heteromastus filiformis*, the deposit feeding polychaetes *Chaetozone setosa*, *S. kroeyeri*, *Aphelochaeta marioni*, as well as the carnivorous polychaete *Paramphinome jeffreysii*, constituted 50–67% of the total abundance but only contributed to 7% of the dissimilarities between the two fields (Table S4 SIMPER). The similarity between the fields is also illustrated by the comparable pattern in feeding guilds and taxonomic groups (Fig. 3d).

### Clay capping

The community in Clay-30 showed a significant difference in abundance compared to the uncapped Ref-30 (Table 3). However, the abundance was only significantly higher after 1 month and not after 14 months. Further, no differences in number of species or total biomass as well as no significant differences over time were observed (Table 3). The relative composition in feeding guilds and taxonomic groups in Clay-30 was similar to Ref-30, and the communities were dominated by the same species (*A. filiformis*, *C. gibba*, and *S. inflatum*). Besides, there were rather similar communities in Clay-30 and Ref-30 (around 50% dissimilarity, Table S3 SIMPER). The cluster analysis also confirmed the similarities between the Ref-30 and the Clay-30 fields (Fig. 2a). Since the Clay-30 field lacked negative effects of capping, in combination with the similarity to the Ref-30 field, it was used as a capping control to isolate the capping effects of AC in AC+clay-30.

### Limestone capping

The benthic community exposed to the Lime-30 treatment was significantly different from the community in Ref-30 (Table 3b). The dissimilarities were highly influenced by large dispersion among samples in Lime-30 (PERM-DISP, *p* < 0.5), and no significant differences were observed for the number of species or abundance. Similar to the Ref-30 and Clay-30 fields, Lime-30 was dominated by the suspension feeding brittle star *A. filiformis*, and also had high abundance of the filter feeding bivalve *C. gibba*. Nonetheless, the relative composition of the taxonomic groups differed after 1 month, with lower abundances of echinoderms (mainly suspension feeders) and higher abundances of polychaetes (mainly deposit feeders) compared to Ref-30 and Clay-30. Lime-30 showed higher abundances of opportunistic species e.g., the subsurface deposit feeding bivalve *Thyasira flexuosa*, together with the small deposit feeding polychaetes *Prionospio fallax* after 1 month and *Polydora* spp. after 14 months. *A. filiformis*, *T. flexuosa* and *Polydora* spp. contributed strongly to the increase in abundance, although not significant, between month 1 and 14 in Lime-30. In general, Lime-30 had a higher number of unique species, with 27 out of 73 species only found in this field compared to 4–13 unique species in the other treated fields. Further, the significantly lower biomass observed 1 month after capping was connected to lower records of large species, especially sea urchins. Remains of recently crushed sea urchins in the samples indicate that these initially suffered from the limestone treatment. However, their subsequent recovery reestablished a high biomass 14 months after capping with limestone (Fig. 3c).

### Activated carbon capping

#### AC+clay at 30 m

The benthic community was seriously disturbed in the AC+clay field at 30 m depth. One month after capping, the benthic community in AC+clay-30 had a 48% lower abundance compared to Ref-30, and a 72% lower abundance compared to the capping control treatment Clay-30 (Fig. 3c). After 14 months, the benthic community in the AC+clay-30 treatment showed an even more severe deterioration. Compared to the records in Ref-30 and Clay-30, the abundance was 90–92% lower, the number of species 77–78% lower and the total biomass 76–89% lower. The dissimilarities in community structure were 83–86% compared to Ref-30 and Clay-30 after 14 months (Table S3 SIMPER). The reduced abundance and number of species in AC+clay-30 after 14 months was also apparent in the high dispersion among samples (PERM-DISP, *p* = 0.001). This is illustrated by the widely spread data points in the n-MDS-plot (Fig. 2b) as well as high dissimilarity among samples (88%, Table S3 SIMPER).

The initial degradation of the benthic community in the AC+clay-30 field was principally due to a lower abundance of suspension and filter feeders e.g., the brittle star *A. filiformis* and the bivalve *C. gibba*. Already after 1 month, *A. filiformis* was up to 98% less abundant in AC+clay-30 compared to Ref-30 and Clay-30. The decrease in abundances after 1 month was followed by a dramatic loss in the number of species within all functional groups after 14 months (Fig. 3a). The diversity poor community in the AC+clay-30 field was after 14 month maintained by tolerant or opportunistic subsurface carnivores, e.g., the polychaetes *Abyssoninoe hibernica* and *Nephtys incisa* together with the mollusk *Philine scabra*. After 14 months, AC+clay-30 had four filter feeding or suspension feeding species compared to seven and nine species in Ref-30 and Clay-30 respectively.

Crustaceans were generally scarce in all four fields at 30 m depth, and in the AC+clay-30 field only one single crustacean specimen was found 1 month after capping (the deep burrowing deposit feeding ghost shrimp *Callianassa subterranea*). Species considered generally as more stress-tolerant e.g., the opportunistic subsurface deposit feeding polychaete *S. inflatum* and the tolerant filter feeding bivalve *C. gibba* were abundant in all four fields at 30 m depth. However, they were found at much lower densities in the AC+clay-30 field. In the SIMPER analysis, the brittle stars *A. filiformis* together with the polychaete *S. inflatum* constituted the most prominent reduction in AC+clay-30 compared to Ref-30 and Clay-30 (Table S3 SIMPER). The biomass after 1 month in AC+clay-30 was generally kept high by large specimens of the deposit feeding sea urchin *E. cordatum*. However, nearly all burrowing sea urchins had disappeared from the AC+clay-30 field after 14 months (only one *B. lyrifera* and no *E. cordatum* were found). The reduction in large species like the sea urchins together with the absence or low frequency of intermediate sized and normally abundant species in the area (such as the brittle star *A. filiformis*, the carnivorous nemertean worm *Cerebratulus* spp. and the subsurface deposit feeding mussel *Pectinaria belgica*) represented almost the entire difference in biomass in AC+clay-30 compared to Ref-30 and Clay-30.

The analysis regarding changes in average individual weight was possible for 25 species at 30 m depth. After 14 months, 7 out of 9 worms (i.e., polychaete, nemertean and sipuncoloid species) had lower average individual weight in AC+clay-30 compared to Ref-30 and Clay-30. Five of the worms with lower individual weight were also classified as carnivores, the only feeding guild with a majority of the species (5 out of 7) having a lower average individual weight in AC+clay-30. In contrast, 4 out of 5 molluscs had higher individual weight in AC+clay-30 after 14 months.

#### AC+clay at 95 m

Capping with AC+clay at 95 m depth also caused negative effects on the benthic community. The largest effects were observed 1 month after capping, with decreases in total abundance (33%), number of species (37%) and biomass (91%) compared to the Ref-80 field (Fig. 3). After 14 months, the number of species remained low compared to both Ref-80 (34% lower) and Ref-95 (17% lower). There was a small increase in abundance between 1 and 14 months in AC+clay-95, but since this was a general increase in the area, AC+clay-95 still had 32 and 34% lower abundance compared to Ref-80 and Ref-95, respectively (although only significantly compared to Ref-95). The biomass in AC+clay-95 had increased until month 14 but were still significantly lower compared to Ref-80, but not to Ref-95. The community structure in AC+clay-95 showed higher dissimilarity to Ref-80 (51–57%) compared to Ref-95 (42%) (Fig. 2c, Table S4 SIMPER).

The difference in abundance in AC+clay-95 compared to Ref-80 were mainly attributed to lower records of deposit feeders and carnivores after 1 month and to deposit and subsurface deposit feeders after 14 months. The number of subsurface deposit feeding species was reduced (from ten to five) between 1 and 14 months, whereas carnivore species more than doubled (from eight to 18). However, most carnivore species in AC+clay-95 were only represented by one or a few specimens. Polychaetes were in general negatively affected in the AC+clay-95. All fields at 80–95 m depth had high abundances of the deposit feeding polychaete *S. kroeyeri*, but their abundances were lower in the AC+clay-95 field at both sampling occasions compared to the references. The deposit feeding cirratulid polychaetes *A. marioni* and *C. setosa* had strong recruitments in the reference fields after 14 months, while they had similar abundance as after 1 month in the AC+clay-95 field. On the other hand, the rather tolerant carnivorous polychaete *P. jeffreysii* had a strong recruitment after 14 months at 80–95 m depth, also in the AC+clay-95 field. The deposit feeding bivalve *Abra nitida* also had a strong recruitment but only in the AC+clay-95 field. The subsurface bivalve *T. equalis*, relatively common at 80–95 m depth, was also generally less affected by the AC.

The large difference in biomass between AC+clay-95 and Ref-80 after 1 month was due to an initial loss of large organisms such as the sea urchin *B. lyrifera*, but also intermediate sized but often more abundant polychaete species like the deposit feeding *Streblosoma bairdi* and the subsurface deposit feeding *Lipobranchius jeffreysii*. Smaller and abundant deposit feeding polychaetes like *Pista cristata*, *A. marioni* and *C. setosa* as well as less frequent carnivores, deposit and subsurface deposit feeders also contributed, but to a lesser extent, to the difference compared to the references. The lower biomass of deposit feeders alone constituted 90% of the difference in biomass between AC+clay-95 and Ref-80 after 1 month. The increased biomass in AC+clay-95 after 14 months was mainly due to one large sea urchin (*B. lyrifera*), which constituted 63% of the total weight in the AC+clay-95 samples. Excluding the weight of all sea urchins (*B. lyrifera* and *Echinocardium flavescens*) from the evaluation, the biomass increase in AC+clay-95 was only 5% between month 1 and 14, compared to a 33% increase in Ref-80 after exclusion of sea urchins.

## Discussion

### Reference fields

The sediment conditions and TOC content at 30 m depth resemble a typical transport bottom, and is therefore representative for a widespread and common habitat in the area. There was a general pattern in functional group composition in Ref-30 and the capping control Clay-30, as well as in the Lime-30 field, and the dominating species are among the most frequently occurring in the North Sea region. In habitats with greater water movements and low sediment TOC content, abundant filter and suspension feeders like *A. filiformis* play an important role in the benthic-pelagic coupling by their filtering activity and subsequent biodeposition of organic material (Loo et al. 1996; Rosenberg 2001; Solan and Kennedy 2002). Their deposition of fecal pellets contributes with a vital food source to many species in the benthic community (Frankenberg and Smith 1967).

The sediment conditions at 80–95 m depth was typical for a less exposed environment with a higher TOC input (compared to at 30 m depth), making this location representative for another common benthic habitat. Compared to 30 m, the benthic community at 80–95 m depth was more dominated by deposit feeders e.g., the polychaetes *S. kroeyeri*, *H. filiformis* and species of Cirratulidae together with the bivalves *A. nitida* and *T. equalis*. The analysis of the reference fields at 80–95 m depth showed general similarities between the two reference fields. This indicates that both the Ref-80 and the Ref-95 fields can be used as valid references for effect comparisons with the AC+clay-95 field in the univariate and the SIMPER analyses.

### Clay capping

Capping with clay in Clay-30 showed no negative effects on organism abundance, number of species and total biomass. This result confirms previous observations of no negative effects from thin deposits of clay on macrofauna (Näslund et al. 2012; Trannum et al. 2010) as well as on meiofauna and bacterial communities (Näslund et al. 2012). The high abundances of the suspension feeder *A. filiformis* and the filter feeder *C. gibba* suggest that the pulse of clean clay had no smothering effects on suspension and filter feeders. The higher biomass, although not significant, in Clay-30 at both sampling occasions indicates that the addition of clean clay may have attracted large deposit feeders such as the sea urchins *B. lyrifera* and *E. cordatum* and the sea cucumber *Mesothuria intestinalis*. Sedimenting clay particles can adsorb and aggregate with the dissolved and particulate organic material in the water layers above the bottom, resulting in an extra pulse of organic matter, i.e., more food, to the benthic community. Nevertheless, the benthic community in the Clay-30 field showed similar population dynamics with time as Ref-30, e.g., strong recruitment of the burrowing polychaete *S. inflatum*. The similarities between Clay-30 and Ref-30 confirm that the clay treatment can serve as a complement to the reference field in the analyses, primarily as a control in order to isolate the effect of activated carbon in AC+clay-30 but also in the evaluation of capping with limestone. The lack of negative effects from the clay treatment also suggests that clay can be used as a carrier material for an active sorbent such as AC.

### Limestone capping

The addition of limestone initially harmed large species such as the burrowing sea urchins. Some of the sea urchins may have moved away from the field after addition of the limestone material, but some of them appeared to have been trapped or crushed by the coarser particles of the limestone material since remains of sea urchins were found in the samples. However, a recovery had occurred after 14 months and at that time point no significant differences were found compared to Ref-30 in abundance, number of species, or biomass. The large number of unique species together with high dispersion (i.e., large variety in species composition) can be linked to the higher variability in capping thickness and a wider range in particle size in Lime-30 compared to the other fields. The mosaic-like pattern in the limestone treatment probably promoted a heterogeneous species composition. This is also illustrated by the scattered distribution in the n-MDS plot. Recruitments of many unique species suggest that the field had been altered, but not at the expense of the original macrofauna species. Altogether, this suggests that limestone had only short-term negative ecological effects on benthic organisms.

### Capping with activated carbon

The lower records in number of species, abundance, and biomass in the AC+clay fields compared to the references and to the clay field clearly demonstrate the deleterious effects from the AC+clay treatment on the benthic communities at both 30 and 95 m depth. The effects were most severe on the benthic community exposed to AC+clay at 30 m depth, with reductions up to 90% in abundance, number of species, and biomass. The low numbers of species and the few remaining individuals after capping with AC+clay resulted in dissimilar samples with a widely dispersed pattern in the n-MDS (Fig. 2b), which is characteristic for highly disturbed communities (Warwick and Clarke 1993). In general, most species were affected, which was reflected by an overall reduction in all feeding guilds and in all taxonomic groups. Though the negative effects of AC+clay were broad and general, the suspension feeder *A. filiformis* was particularly affected. The fact that the community in AC+clay-30 was more affected after 14 months than after 1 month suggests that the treatment with powdered AC is likely to have a long-term negative impact on this benthic community.

At the deeper location (80–95 m), the AC+clay treatment led to an initial 91% decrease in biomass due to the loss of, or reduction in, several large- and medium-sized organisms. Moreover, a multiple number of species were associated with a decrease in the abundance. Since the reductions in abundance and biomass were observed for nearly 80 out of 112 species at 80–95 m, the AC+clay treatment obviously had a negative influence on a major part of the community. Although the effects were not as severe as for the community at 30 m depth, no sign of recovery were observed after 14 months in the AC+clay-95 community.

#### Responses of benthic species to activated carbon

The negative effects on benthic fauna documented in the AC+clay-30 field were not observed in the Clay-30 field, despite the thinner cap layer in AC+clay. The negative effects from AC+clay would thereby be linked to the activated carbon rather than to the thickness of the capping layer. Benthic invertebrates have shown negative effects from AC treatments in 18% out of 82 tests with 18 species (Janssen and Beckingham 2013), including negative effects on lipid content, growth, behavior, reproduction, survival, and damage of gut microvilli (Jonker et al. 2009; Lillicrap et al. 2015; Macleod et al. 2008; McLeod et al. 2008; Millward et al. 2005; Nybom et al. 2012). However, the effects are not general and seem to be rather species-specific, although most of the negative effects from AC so far have been connected to worms (oligochaetes and polychaetes). On the other hand, worms are also somewhat overrepresented in the literature. The present study with 158 species clearly demonstrates that other organisms than worms can suffer from activated carbon. The eradication of the otherwise dominant suspension feeding brittle stars *A. filiformis* contributed largely to that the total abundance in AC+clay-30 was only 8–10% compared to Ref-30 and Clay-30 after 14 months.

Carnivores appeared to be less disturbed by the AC, especially at the deeper location. However, carnivores showed a reduction in mean individual weight after 14 months at 30 m depth. All of these carnivores were worms (four polychaete species and one nemertean). Moreover, worms (polychaetes) at the deeper AC-field also showed an overall reduction in mean individual weight, and as mentioned above, worms have often been reported to show reduced growth in AC treatment. The carnivores may initially have benefited by easy prey suffering from the AC treatment. A subsequent lack in prey in the AC+clay-30 field could then have affected growth which was reflected after 14 months in the reduction in number of carnivore species, their abundance, and biomass. At 30 m depth, no recruitment of opportunistic or tolerant species had occurred 14 months after capping with AC+clay. The normally greatly abundant and relative tolerant brittle stars *A. filiformis* were practically eliminated, and the opportunistic worm *S. inflatum* which had a strong recruitment in the other fields had very low densities in the AC+clay-30 treatment. Even the filter-feeding bivalve *C. gibba*, documented as an opportunist and tolerant to several different stressors (heavy metals, organic enrichment, deoxygenation; MarLIN 2006), was negatively affected by the powdered AC in this study. Since no other opportunistic or tolerant species compensated for the apparent reductions, it appears that no benthic species in the fjord area were fit to cope with the AC-altered habitat at 30 m still 14 months following the capping.

The benthic community at 95 m depth showed less negative effects of AC compared to the community at 30 m depth. The deeper community consisted of a higher proportion of burrowing deposit and subsurface deposit feeders and a lower proportion of suspension and filter feeders. The abundances of suspension and filter feeders commonly decrease with larger water depths (Rosenberg 2001), and accordingly the benthic community in AC+clay-95, with naturally lower proportions of AC susceptible suspension and filter feeders, showed less negative effects compared to the AC exposed community at the 30-m-deep location. Since the species community was less affected by AC, a higher proportion of species could bioturbate the AC capping i.e., mix AC from the surface into a deeper zone in the sediment. Thus, the subsurface deposit feeders would be more exposed to the AC by 14 months, which could explain why this group decreased 14 months after AC+clay capping in the field at 95 m depth. The increase of carnivores contributed to that the AC+clay-95 community had shifted from a deposit and subsurface deposit feeder-dominated community to a carnivore and deposit feeder-dominated community after 14 months. The large abundance and low biomass for carnivores indicate that small specimen, either new recruits or small species, characterized this group after 14 months. A lowered AC concentration in the surface layer may have allowed the recruitment of species. In addition, the deeper area receives approximately three times more sedimented material than the shallower area. Hence, after 14 months the concentration of AC in the upper zone of the sediment was probably higher in the 30-m-deep field compared to the 95 m, and the higher AC concentration in the surface layer has probably prevented recruitment to a larger extent at 30 m than at 95 m.

The general negative impact on the majority of the deposit feeding and subsurface deposit feeding species was contrasted by the apparent resistance to the AC cap by the bivalves *T. equalis* and *A. nitida*. The subsurface deposit feeding *T. equalis* lives deep burrowed in the sediment where AC particles probably were more or less absent, and the deposit feeding *A. nitida* is able to sort feeding particles. Molluscs were also the only group showing higher mean individual weight, both at 30 and 80–95 m. The protection inside a shell, especially for the bivalves, probably helps the animal to reduce negative effects from direct contact to AC particles. In contrast, sediment-dwelling organisms without protection, such as polychaetes, have shown physical interference from AC particles sticking onto epidermal tissues (Lillicrap et al. 2015).

#### Particle size and concentration of AC

In the present study, powdered AC with 80% of particles smaller than 45 μm was used. Both AC concentration and particle size are important factors for if, and how much, the benthic organisms are affected. In general, higher concentrations of AC (>5%) and smaller particle sizes (<200 μm) have been associated with negative impact on benthic invertebrates (Jonker et al. 2004, 2009; Kupryianchyk et al. 2013a; Nybom et al. 2012, 2015; Rakowska et al. 2012). Milder effects can be expected if AC is mixed into a non-active carrier such as clay (Cornelissen et al. 2011; Jonker et al. 2009; Nybom et al. 2012), but this study demonstrates that effects from powdered AC was not eliminated in this habitat, although AC was mixed with clay. AC concentration may be less important for granular AC if the negative effects from AC mostly are connected to the small particle sizes (Nybom et al. 2012). Powdered AC interferes with the preferable particle size range in feeding activities for many of the exposed organisms. Besides, fine AC particles seem to have sharp edges that can harm the gut of benthic organisms when ingested (Nybom et al. 2015). Species with activities at the sediment-water interface would be the ones most exposed to the fine particles of AC used in this study and accordingly, it is primarily the suspension and surface deposit feeding species that were the most affected in our study. Mixing of the AC in clay together with loss of AC particles and a further mixing and dilution in the bottom sediment resulted in a final 2% AC dry weight concentration in the AC+clay treatments (Cornelissen et al. 2012). Thus, the small particle size of the AC, rather than the concentration, may explain the severity of the deleterious effects on benthic communities in this study. A slightly larger particle size of AC is probably more benign to the benthic community. However, coarse AC may be less effective for contaminant sequestration. Therefore, it is crucial to find an intermediate particle size between powdered AC and coarse AC that still meets the goals in contaminant sequestration without being disruptive to benthic communities.

#### Reduced amount of available organic carbon for benthos

Negative effects from AC have also been suggested to be due to the sorption of available organic carbon to the AC particles (Jonker et al. 2004), since AC binds all types of organic carbon, including carbohydrates, fat, and proteins (Aitcheson et al. 2000, 2001). The TOC content was lower at 30 m depth in the Ormerfjord compared to 95 m in the Eidangerfjord (Table 2). The effect of AC competing for the already limited amount of TOC would lead to less available nutritive organic carbon for the benthic organisms and can thus be a complementary explanation to the negative effects, especially at 30 m. Moreover, a vital part of the carbon input to the benthic community is administered by biodepositing organisms (e.g., filter and suspension feeders) capturing organic matter from the water column and their subsequent deposition of fecal pellets in the sediment, thus increasing the available food for the benthic organisms (Frankenberg and Smith 1967). The loss of filter and suspension feeders, mostly at 30 m, may therefore have led to a negative feedback loop with reduced carbon input to the benthic ecosystem. On the other hand, species able of extracting energy from alternative sources would be less dependent of available organic material and could consequently be less affected, given that they are not negatively affected by contact with AC. Thyasirid bivalves like *T. equalis* hosts symbiotic sulfidic bacteria which can contribute to more than 50% of their energy demand (Spiro et al. 1986), and the carnivorous polychaete *P. jeffreysii* is suggested to feed on shell-protected foraminifers. These two species were among the few species occurring at high numbers in AC+clay.

The differences in TOC content may explain the contradictory results among AC capping studies. The negative effects to the marine communities documented in Trondheim Harbor (Cornelissen et al. 2011) may, e.g., have been facilitated by a multiple stressor situation from small particle-sized AC (<45 μm), contaminants in the sediment, and relatively low levels of organic carbon (TOC 2.5%). The marine benthic community in a boxcore study exposed to powdered AC (<40 μm) and with similar levels of organic carbon (TOC 2.7%) showed only moderate effects of AC (Näslund et al. 2012), but more severe effects were probably avoided by the monthly addition of food in that study. Other studies on community responses to AC have often showed less negative effects, where causes for less susceptibility may be attributed to factors keeping up the resilience in these systems. For example, the larger AC particles (100–200 μm) and relative organic carbon-rich river sediment (TOC 4–6%) may explain the limited negative effects to a fresh water community in Grasse River (Beckingham et al. 2013). Moreover, the mild initial effects and full recovery of the benthic community 1 year after exposure to powdered AC in an un-contaminated fresh water experimental ditch may also be attributed to less stress due to organic carbon rich (TOC 8%) sediment (Kupryianchyk et al. 2012). Hence, the negative response of the benthic community by powdered AC at 30 m depth in the present study may have been determined by a multitude of variables, e.g., poor available organic carbon (TOC only 1.1–1.6%), in concert with organic carbon binding by AC, reduced biodeposition, and physiologically negative effects by the fine, and possibly sharp, AC particles.

#### Consequences for ecosystem functioning

The degree of stress already present in the system can have influenced how the benthic community responded to the additional stress from AC. The high levels of contaminants (dioxins, furans and mercury) may already have affected the benthic community and reduced its resilience to withstand additional stress from AC. For example, the low numbers of crustaceans especially at 30 m may be a consequence of a contaminant-related stress prevalent in the system already before the AC capping. Additional stress from AC and its sequestration of the limited organic carbon in marine environments will probably reduce the overall carrying capacity of benthic ecosystems and lower the capacity to harbor a specific level of biomass or complexity in a community. In AC+clay-95, there was also a general shift in the community towards smaller organisms. The original community seems to have been replaced by a community with fewer and generally smaller and more tolerant species. However, it appears not to be a successional stage with a clear shift towards opportunistic species since also many of these were still reduced in abundance after 14 months. This means that 14 months after capping, practically all species were suffering from the AC+clay treatment and no species had yet been able to exploit the void in space and niche. This can also indicate a reduction in overall carrying capacity. A consequence of a decreased biomass in the benthic system may have negative trophic effects on demersal fish and other organisms higher up in the food web. The benthic organisms are the most exposed to AC capping, and consequently most studies have focused on the direct effect on the benthos. However, cascading effects at higher trophic levels, e.g., on fish, can occur as shown by Kupryianchyk et al. (2013b), who observed a reduced weight in fish (*Leuciscus idus melanotus*) after capping with powdered AC. Moreover, fish and lobsters grazing on arms of the brittle star *A. filiformis* (Baden et al. 1990; Duineveld and van Noort 1986; Mattson 1992) would probably be affected by the disappearance of an important part of their diet.

A loss of bioturbating organisms, like the affected brittle stars and sea urchins in this study, may also lead to less mixing of the AC into the contaminated sediment (and increased time for contaminant-AC association) thus counteracting the efficiency of the remediation. In addition, a loss of bioturbating fauna may also affect the important regeneration and circulation of nutrients (Rhoads and Germano 1986; Snelgrove et al. 1997). As an example, the reduction in biomass e.g., associated to the loss of the sea urchins *E. cordatum* and *B. lyrifera* in the AC+clay-30 treatments may have large negative effect on several ecosystem functions, since the loss of large and late successional bioturbators like sea urchins has been linked to changes in nutrient circulation and reduced ecosystem productivity (Lohrer et al. 2004). Moreover, the brittle star *A. filiformis* has been found to account for up to 80% of the total flux of oxygen into the sediment (Vopel et al. 2003). Thus, the loss of the brittle star *A. filiformis* in AC+clay-30 may have rendered a less oxygenated sediment. Hence, loss of the bioturbating brittle stars and sea urchins may lead to lower sediment oxygen levels, as well as a degradation of ecosystem services (Worm et al. 2006).

### Considerations

Capping with powdered activated carbon appears to have altered the habitat profoundly and rendered it less suitable for the macrofauna species. However, these negative effects may be temporary and it is important to follow the capped communities over a longer time span in order to study their potential recovery. If the benthic community recovers after a few more years, then thin-layer capping with AC may be a promising remediation technique since the AC treatment has showed promising results regarding contaminant sequestration (Cornelissen et al. 2015). On the other hand, if the negative effects on the benthic community are long-lasting, then this remediation option needs to be modified and tested to be less disruptive before it could be recommended for remediation in this type of ecosystem. In confined highly polluted hotspots, it may be rational to accept a degradation of the benthic fauna after capping with AC, especially if the benthic community already is severely impoverished and the ecosystem services is degraded. In such cases, it is important to have untreated interconnected areas from which organisms can recolonize the capped area. After a completed remediation, these organisms could eventually revitalize the ecosystem services. However, in cases as in the Grenland fjord, with large areas of polluted sediments that function as large and diffuse sources for contaminant dispersion and transfer to higher trophic layers, hotspot treatments are not sufficient. An advantage of thin-layer capping with an active sorbent, such as AC, is that this technique requires a relatively small amount of material, making it a viable option for treating larger areas. Therefore, it is crucial to modify the technique to be less disruptive to ecosystem components. When larger areas are intended for remediation, the negative effects of AC on benthic species should be carefully considered. The information from the community analysis in this study highlights that more research is necessary to reveal the underlying mechanisms on how AC affects e.g., filter and suspension feeders on one hand and polychaetes on the other hand. It is important to make considerations concerning AC particle size, AC concentration, available organic carbon, and the benthic community structure and function before thin-layer capping with AC can be recommended as a remediation option.

## Conclusions

The present study shows that thin-layer capping with only clay was not harmful to the benthic macrofauna communities. Capping with crushed limestone had initial negative effects, especially on the biomass, but the community had recovered after 14 months. Although capping with powdered AC mixed with clay (AC+clay) has the potential to effectively sequester sediment contaminants, it was here found to severely reduce the abundance, number of species, and biomass up to 14 months after the capping operation. In general, all feeding guilds were negatively affected by AC, but the filter and suspension feeders appear particularly sensitive. The reasons behind the negative response of AC+clay can be due to less food availability in combination with ecotoxicological effects. More research on the ecological effects of AC materials is needed before capping with AC can be advocated for sediment remediation, since this study emphasizes stronger disturbances to the benthic community than previously has been reported.

## Electronic supplementary material

ESM 1 (PDF 1.52 mb)
