# Supplementary material for: Response of marine benthic fauna to thin-layer capping with activated carbon in a large-scale field experiment in the Grenland fjords, Norway
Source: Environ Sci Pollut Res Int. 2017 Apr 18;24(16):14218–33. doi: 10.1007/s11356-017-8851-6 (PMC5486621; doi:10.1007/s11356-017-8851-6)
Supplement: Supplementary file 1 — (PDF 1.52 mb) [file 11356_2017_8851_MOESM1_ESM.pdf]

Supplementary material

Response of marine benthic fauna to thin-layer capping with activated carbon in a large-scale field experiment in the Grenland fjords, Norway

Göran S. Samuelsson[1,2*], Caroline Raymond[1*], Stefan Agrenius[3], Morten Schaanning[4], Gerard Cornelissen[5,6], Jonas S. Gunnarsson[1]

[1] Department of Ecology, Environment and Plant Sciences (DEEP), Stockholm University, 106 91 Stockholm, Sweden.
[2] Present address: Svensk Ekologikonsult, Vegagatan 3, 113 29 Stockholm, Sweden.
[3] Department of Marine Sciences-Kristineberg, University of Gothenburg, 451 78 Fiskebäckskil, Sweden.
[4] Norwegian Institute for Water Research (NIVA), 0349 Oslo, Norway.
[5] Norwegian Geotechnical Institute (NGI), P.O. Box 3930 Ullevål Stadium, 0806 Oslo, Norway.
[6] Faculty of Environmental Science and Natural Resource Management, Norwegian University of Life Sciences (NMBU), 1432 Ås, Norway.

* Corresponding authors. E-mail address: goran@systemsecology.se, caroline.raymond@su.se

---

Table S1 (a-g). Species list with abundance and biomass per grab

Table S2. Feeding guild and taxonomic classification

Table S3. SIMPER-analysis from 30 m

Table S4. SIMPER-analysis from 80-95 m

Number of Pages: 33

Table S1. Species list. Abundance (Abu) per grab and Biomass (Bio) g wet weight per grab.

a. Lime-30

Phylum	Species	Lime-30:1A		Lime-30:1B		Lime-30:1C		Lime-30:14A		Lime-30:14B		Lime-30:14C		Lime-30:14D		Lime-30:14E	
		Abu	Bio	Abu	Bio	Abu	Bio	Abu	Bio	Abu	Bio	Abu	Bio	Abu	Bio	Abu	Bio
Annelida	*Abyssoninoe hibernica*					1	0.005									1	0.005
Annelida	*Ampharete baltica*															3	0.020
Annelida	*Anobothrus gracilis*									1	0.030	1	0.030	4	0.005		
Annelida	*Brada villosa*									1	0.005	1	0.005				
Annelida	*Chaetopterus norvegicus*													1	2.450		
Annelida	*Chaetozone setosa*	1	0.005			3	0.005			1	0.010	2	0.020				
Annelida	*Diplocirrus glaucus*	1	0.010			3	0.010	2	0.010					9	0.020		
Annelida	*Eclysippe eliasoni*							4	0.005								
Annelida	*Euchone papillosa*							1	0.005			3	0.005				
Annelida	*Galathowenia oculata*					5	0.010	14	0.150	16	0.130	1	0.005				
Annelida	*Glycera alba*	2	0.010	2	0.010	5	0.060	1	0.060	1	0.010			2	0.090		
Annelida	*Goniada maculata*			1	0.050							2	0.060				
Annelida	*Heteromastus filiformis*	1	0.005			9	0.010			1	0.005						
Annelida	*Laonice bahusiensis*					1	0.020										
Annelida	*Lipobranchius jeffreysii*							1	0.020							1	0.050
Annelida	*Lumbriclymene minor*							1	0.005								
Annelida	*Magelona minuta*	1	0.005														
Annelida	*Maldane sarsi*					4	0.230			1	0.040						
Annelida	*Nephtys incisa*											1	0.040				
Annelida	*Ophiodromus flexuosus*					1	0.005	1	0.020								
Annelida	*Owenia fusiformis*					1	0.190			1	0.010						
Annelida	*Pectinaria auricoma*											4	0.010	1	0.005	1	0.005
Annelida	*Pectinaria koreni*											2	0.070			2	0.570
Annelida	*Pholoe baltica*			1	0.005	1	0.010					1	0.005	6	0.010		
Annelida	*Pholoe pallida*	1	0.005														
Annelida	*Pilargis verrucosa*			1	0.030												
Annelida	*Podarkeopsis helgolandicus*					1	0.010										
Annelida	*Polydora* spp.					1	0.005	29	0.010	31	0.010	4	0.005				
Annelida	*Praxillella praetermissa*	1	0.005	1	0.005	2	0.005			3	0.080						
Annelida	*Prionospio cirrifera*							1	0.005								
Annelida	*Prionospio fallax*	9	0.010	3	0.005	11	0.010										
Annelida	*Prionospio multibranchiata*	1	0.005														
Annelida	*Rhodine loveni*									1	0.020						
Annelida	Sabellidae									1	0.005						
Annelida	*Scalibregma inflatum*	2	0.400			5	0.410			4	0.020	17	0.360	7	0.080	7	0.030
Annelida	*Spiophanes kroeyeri*											2	0.020				
Annelida	*Terebellides stroemi*													1	0.005		
Annelida	*Tharyx killariensis*	2	0.005	2	0.005												
Annelida	*Trichobranchus roseus*					3	0.030	6	0.020	4	0.030						
Arthropoda	*Ampelisca gibba*									1	0.005						
Arthropoda	*Ampelisca macrocephala*							1	0.005								
Arthropoda	*Callianassa subterranea*															3	0.070
Arthropoda	*Diastylis boecki*							1	0.010	1	0.010					1	0.005
Arthropoda	*Eriopisa elongata*															1	0.005
Arthropoda	*Pagurus bernhardus*					1	2.080										
Cnidaria	Anthozoa							1	0.005			1	0.030				
Cnidaria	*Cerianthus lloydii*									1	0.005						
Cnidaria	Edwardsiidae	1	0.005					2	0.060	3	0.005	1	0.005				
Echinodermata	*Amphiura chiajei*	2	0.086			5	0.017	3	0.162	5	0.141	1	0.227			2	0.549
Echinodermata	*Amphiura filiformis*	17	0.804	2	0.020	16	0.083	24	0.568	22	0.759	50	2.493	14	0.270	42	4.191
Echinodermata	*Brissopsis lyrifera*									1	5.820					5	28.510
Echinodermata	*Echinocardium cordatum*									1	2.210						
Echinodermata	*Labidoplax buskii*					1	0.010	5	0.010								
Echinodermata	*Luidia sarsi*	1	0.010														
Echinodermata	*Ophiocten affinis*															2	0.005
Mollusca	*Antalis entalis*											1	0.640			1	0.540
Mollusca	*Chaetoderma nitidulum*							1	0.100								
Mollusca	*Corbula gibba*	14	0.800	2	0.005	7	0.630	3	0.170	11	0.510	7	0.290	4	0.040	1	0.020
Mollusca	*Cylichna cylindracea*	1	0.020									1	0.010				
Mollusca	*Ennucula tenuis*							5	0.430	2	0.240						
Mollusca	*Hyala vitrea*			3	0.010	2	0.005			1	0.005	9	0.030			3	0.005

Phylum	Species	Abu	Bio	Abu	Bio	Abu	Bio	Abu	Bio	Abu	Bio	Abu	Bio	Abu	Bio	Abu	Bio
Mollusca	*Mysella bidentata*											5	0.005				
Mollusca	*Nucula nitidosa*							2	0.100								
Mollusca	*Parvicardium minimum*									1	0.005						
Mollusca	*Parvicardium pinnulatum*							1	0.050								
Mollusca	*Philine scabra*							1	0.010	3	0.050	1	0.005	3	0.070		
Mollusca	*Polinices montagui*	1	0.080														
Mollusca	*Polinices pulchella*	2	0.050														
Mollusca	*Thyasira flexuosa*	7	0.100			3	0.040	33	0.490	24	0.420	2	0.030				
Nemertea	*Cerebratulus* spp.	1	0.005					2	0.150								
Nemertea	Nemertea			1	0.005	9	0.010									1	0.090
Sipuncula	*Phascolion strombus*					1	0.020										
Sipuncula	*Thysanocardia procera*	1	0.010	1	0.070												

b. Clay-30

Phylum	Species	Clay-30:1A		Clay-30:1B		Clay-30:1C		Clay-30:14A		Clay-30:14B		Clay-30:14C		Clay-30:14D		Clay-30:14E	
		Abu	Bio	Abu	Bio	Abu	Bio	Abu	Bio	Abu	Bio	Abu	Bio	Abu	Bio	Abu	Bio
Annelida	*Abyssoninoe hibernica*	3	0.060	1	0.010	8	0.210	1	0.030	1	0.020	1	0.040			1	0.005
Annelida	*Ampharete finmarchica*									4	0.010						
Annelida	*Chaetopterus norvegicus*															1	0.120
Annelida	*Chaetozone setosa*															1	0.005
Annelida	*Diplocirrus glaucus*	4	0.030			3	0.020	1	0.010	3	0.005	1	0.005	2	0.020		
Annelida	*Eunoe nodosa*											1	0.005				
Annelida	*Gattyana cirrhosa*											2	0.005				
Annelida	*Glycera alba*			1	0.010			3	0.070			1	0.005	1	0.020	1	0.020
Annelida	*Glycera rouxii*	1	0.080							1	0.220						
Annelida	*Goniada maculata*													2	0.120		
Annelida	*Heteromastus filiformis*	1	0.005													1	0.005
Annelida	*Laonice bahusiensis*	1	0.010														
Annelida	*Nephtys incisa*					1	0.070	3	0.380	2	0.290	2	0.240	2	0.350	1	0.180
Annelida	*Ophiodromus flexuosus*													1	0.005		
Annelida	*Pectinaria auricoma*	1	0.010	3	0.160												
Annelida	*Pectinaria belgica*	2	0.005			2	1.530							1	0.440		
Annelida	*Pectinaria koreni*											2	0.130				
Annelida	*Phyllodoce groenlandica*							1	0.005								
Annelida	*Polydora* spp.	1	0.005					9	0.005			2	0.005				
Annelida	*Polyphysia crassa*			3	0.840	1	0.900										
Annelida	*Praxillella praetermissa*	1	0.030														
Annelida	*Prionospio fallax*									3	0.005						
Annelida	*Psamathe fusca*													1	0.005		
Annelida	*Scalibregma inflatum*	1	0.005					53	0.280	18	0.100	21	0.080	6	0.030	39	0.140
Annelida	*Spiophanes kroeyeri*	1	0.040	2	0.010	1	0.010			2	0.040	1	0.050				
Annelida	*Terebellides stroemi*	3	0.030							1	0.010					4	0.020
Annelida	*Trichobranchus roseus*											1	0.020				
Arthropoda	*Callianassa subterranea*	1	0.005	1	0.010	1	0.010										
Arthropoda	*Eriopisa elongata*					1	0.005			1	0.005						
Cnidaria	Edwardsiidae			1	0.010	1	0.005			4	0.060	1	0.020	1	0.005		
Echinodermata	*Amphiura chiajei*	2	0.390	4	0.596	1	0.303			4	0.877			4	1.205		
Echinodermata	*Amphiura filiformis*	23	0.660	41	1.944	24	1.348	18	0.320	21	1.523	27	1.150	29	2.145	8	0.330
Echinodermata	*Brissopsis lyrifera*	2	25.510			2	17.490	1	4.710	1	6.070	1	7.520	1	6.480	1	2.850
Echinodermata	*Echinocardium cordatum*					2	5.300					3	9.820	2	7.950		
Echinodermata	*Echinocardium flavescens*			1	2.990												
Echinodermata	*Leptopentacta elongata*			1	0.030					1	0.490						
Echinodermata	*Luidia sarsi*	1	0.005														
Echinodermata	*Mesothuria intestinalis*											1	34.700				
Mollusca	*Abra nitida*					1	0.010					1	0.060				
Mollusca	*Antalis entalis*					1	0.680										
Mollusca	*Chaetoderma nitidulum*	1	0.030	1	0.060							2	0.140				
Mollusca	*Corbula gibba*	7	1.000	13	0.500	10	0.630	6	0.540	6	0.430	9	0.500	5	0.050	2	0.330
Mollusca	*Cuspidaria obesa*					1	0.080										
Mollusca	*Cylichna cylindracea*					3	0.050			1	0.020						
Mollusca	*Ennucula tenuis*													1	0.080		
Mollusca	*Hyala vitrea*	19	0.050	2	0.010	4	0.010			3	0.005	1	0.005			1	0.005
Mollusca	*Myrtea spinifera*											1	0.030				
Mollusca	*Mysella bidentata*					2	0.005										

Phylum	Species	Abu	Bio	Abu	Bio	Abu	Bio	Abu	Bio	Abu	Bio	Abu	Bio	Abu	Bio	Abu	Bio
Mollusca	*Nucula nitidosa*	13	0.350	8	0.270	2	0.090					2	0.030	3	0.170	2	0.140
Mollusca	*Phaxas pellucida*											2	0.030				
Mollusca	*Philine scabra*	1	0.010			3	0.050	1	0.060	1	0.010	1	0.005				
Mollusca	*Polinices pulchella*					1	0.150					1	0.140			1	0.070
Mollusca	*Tellimya tenella*					3	0.005										
Mollusca	*Thyasira flexuosa*	4	0.030	5	0.020			7	0.210			3	0.080				
Mollusca	*Thyasira sarsii*													1	0.005		
Nemertea	*Cerebratulus* spp.													3	9.780		
Sipuncula	*Golfingia vulgaris*									1	0.170						
Sipuncula	*Thysanocardia procera*	1	0.060			4	0.240	1	0.150	1	0.050					1	0.060

c. AC+clay-30

Phylum	Species	AC+clay-30:1A		AC+clay-30:1B		AC+clay-30:1C		AC+clay-30:14A		AC+clay-30:14B		AC+clay-30:14C		AC+clay-30:14D		AC+clay-30:14E	
		Abu	Bio	Abu	Bio	Abu	Bio	Abu	Bio	Abu	Bio	Abu	Bio	Abu	Bio	Abu	Bio
Annelida	*Abyssoninoe hibernica*	1	0.020													5	0.120
Annelida	*Chaetopterus norvegicus*															1	1.110
Annelida	*Cossura longocirrata*	1	0.005														
Annelida	*Diplocirrus glaucus*	1	0.010														
Annelida	*Glycera alba*															1	0.005
Annelida	*Goniada maculata*															1	0.020
Annelida	*Heteromastus filiformis*	1	0.010	1	0.005												
Annelida	*Nephtys incisa*	3	0.250	4	0.330			2	0.060	2	0.190						
Annelida	*Notomastus latericeus*									1	0.140						
Annelida	*Polycirrus* spp.	2	0.010			1	0.010										
Annelida	*Scalibregma inflatum*	1	0.010	1	0.030	2	0.180							2	0.030		
Annelida	*Spiophanes kroeyeri*							1	0.010	1	0.030						
Annelida	*Trichobranchus roseus*	1	0.010	2	0.030	1	0.030										
Arthropoda	*Callianassa subterranea*					1	0.020										
Echinodermata	*Amphiura filiformis*			1	0.005	1	0.005	1	0.020								
Echinodermata	*Brissopsis lyrifera*	1	8.790			1	9.250									1	8.840
Echinodermata	*Echinocardium cordatum*	2	8.580	1	3.240	1	5.300										
Echinodermata	*Luidia sarsi*	2	0.010			1	0.005										
Mollusca	*Abra nitida*	1	0.005	2	0.040							1	0.080				
Mollusca	*Corbula gibba*	3	0.730	1	0.005	1	0.330									4	0.310
Mollusca	*Cylichna cylindracea*	2	0.020	1	0.010	5	0.050			1	0.020						
Mollusca	*Hyala vitrea*	5	0.020	2	0.010	2	0.010										
Mollusca	*Montacuta ferruginosa*	3	0.005	1	0.005	1	0.005										
Mollusca	*Nucula sulcata*	1	0.790														
Mollusca	*Parvicardium minimum*	1	0.005														
Mollusca	*Philine scabra*			1	0.010									2	0.050		
Mollusca	*Polinices pulchella*	1	0.030	1	0.005												
Mollusca	*Tellimya tenella*	2	0.010			3	0.010										
Mollusca	*Thyasira sarsii*											1	0.010	1	0.010	1	0.010
Nemertea	*Cerebratulus spp.*											1	0.020				
Sipuncula	*Golfingia vulgaris*									1	0.010						

d. Ref-30

Phylum	Species	Ref-30:1A		Ref-30:1B		Ref-30:1C		Ref-30:14A		Ref-30:14B		Ref-30:14C		Ref-30:14D		Ref-30:14E	
		Abu	Bio	Abu	Bio	Abu	Bio	Abu	Bio	Abu	Bio	Abu	Bio	Abu	Bio	Abu	Bio
Annelida	*Abyssoninoe hibernica*			4	0.080	1	0.030	3	0.100	3	0.070			2	0.090		
Annelida	*Chaetopterus norvegicus*									1	1.370						
Annelida	*Chaetozone setosa*							1	0.005								
Annelida	*Diplocirrus glaucus*	1	0.010	1	0.010			4	0.040	5	0.040	2	0.005	2	0.005	2	0.005
Annelida	*Glycera alba*							1	0.050	1	0.010			2	0.070		
Annelida	*Goniada maculata*			1	0.060	1	0.070	1	0.020								
Annelida	*Heteromastus filiformis*									1	0.020	2	0.010				
Annelida	*Lipobranchius jeffreysii*	1	0.820														
Annelida	Maldanidae									1	0.020						
Annelida	*Nephtys incisa*	1	0.090	1	0.020	1	0.090	1	0.070	1	0.005	2	0.170	1	0.160	1	0.080
Annelida	*Ophiodromus flexuosus*							1	0.040	2	0.030	1	0.020	2	0.060	2	0.040
Annelida	*Pectinaria auricoma*									1	0.005	2	0.010				

Phylum	Species	Abu	Bio	Abu	Bio	Abu	Bio	Abu	Bio	Abu	Bio	Abu	Bio	Abu	Bio	Abu	Bio
Annelida	*Pectinaria belgica*							4	4.290			1	0.005				
Annelida	*Pholoe baltica*											1	0.005	1	0.005	1	0.010
Annelida	*Podarkeopsis helgolandicus*					1	0.010										
Annelida	*Polycirrus* spp.	1	0.020														
Annelida	*Polydora* spp.							1	0.005								
Annelida	*Praxillella affinis*													2	0.070		
Annelida	*Praxillella praetermissa*	2	0.070					3	0.090							2	0.070
Annelida	*Scalibregma inflatum*							23	0.090	56	0.210	10	0.120	35	0.530	8	0.050
Annelida	*Scolelepis tridentata*									1	0.005						
Annelida	*Spiophanes kroeyeri*	2	0.050			2	0.030	2	0.030	2	0.060	2	0.030	2	0.030	3	0.060
Annelida	*Streblosoma bairdi*									1	0.050	1	0.050	1	0.070	2	0.010
Annelida	*Terebellides stroemi*			1	0.010					6	0.030			8	0.090	1	0.020
Arthropoda	*Callianassa subterranea*	1	0.120	2	0.040	1	0.020			1	0.010	3	0.120	3	0.080	3	0.130
Arthropoda	*Diastylis laevis*															1	0.020
Arthropoda	*Eriopisa elongata*	3	0.010							1	0.005						
Arthropoda	Oedicerotidae									1	0.005						
Cnidaria	Edwardsiidae											1	0.005			1	0.005
Echinodermata	*Amphiura chiajei*	1	0.063	1	0.136	1	0.024										
Echinodermata	*Amphiura filiformis*	24	0.907	24	1.634	17	0.496	8	0.240	7	0.100	3	0.090	9	0.590	14	0.500
Echinodermata	*Brissopsis lyrifera*	1	3.180	1	7.890	2	11.070							1	7.690	1	14.720
Echinodermata	*Echinocardium cordatum*			1	5.360			1	3.950							2	7.120
Echinodermata	*Luidia sarsi*	1	0.010														
Mollusca	*Abra nitida*	1	0.040	1	0.060												
Mollusca	*Chaetoderma nitidulum*			1	0.070	1	0.050										
Mollusca	*Corbula gibba*	12	0.610	3	0.580	3	0.190			1	0.005						
Mollusca	*Cylichna cylindracea*	4	0.050			3	0.040									2	0.030
Mollusca	*Hyala vitrea*	2	0.005			2	0.010	1	0.005							1	0.005
Mollusca	*Mysia undata*							1	0.120								
Mollusca	*Nucula nitidosa*	2	0.020					1	0.070			1	0.080				
Mollusca	*Parvicardium pinnulatum*													1	0.050	1	0.070
Mollusca	*Philine scabra*					2	0.010			1	0.010					1	0.040
Mollusca	*Thyasira flexuosa*											1	0.005				
Nemertea	Nemertea					1	1.420			1	0.720	1	0.020	1	0.090		
Sipuncula	*Thysanocardia procera*	2	0.220	1	0.090							1	0.120	2	0.170	1	0.100

e. AC+clay-95

Phylum	Species	AC+clay-95:1A		AC+clay-95:1B		AC+clay-95:1C		AC+clay-95:14A		AC+clay-95:14B		AC+clay-95:14C		AC+clay-95:14D		AC+clay-95:14E	
		Abu	Bio	Abu	Bio	Abu	Bio	Abu	Bio	Abu	Bio	Abu	Bio	Abu	Bio	Abu	Bio
Annelida	*Abyssoninoe hibernica*			1	0.090												
Annelida	*Aphelochaeta marioni*	2	0.020	4	0.030	1	0.020	3	0.020	4	0.040	3	0.020	6	0.160	4	0.040
Annelida	*Aphelochaeta sp.*							1	0.005	1	0.005						
Annelida	*Bylgides elegans*											2	0.020				
Annelida	*Ceratocephale loveni*	2	0.050	5	0.120	1	0.010	4	0.050	3	0.060	3	0.020	1	0.005	2	0.030
Annelida	*Chaetoparia nilssoni*	1	0.005									1	0.010				
Annelida	*Chaetozone setosa*	2	0.010	10	0.080			1	0.005	5	0.005	4	0.020	2	0.020	4	0.020
Annelida	*Diplocirrus glaucus*			1	0.010	1	0.010			1	0.005					1	0.005
Annelida	*Euchone papillosa*					2	0.020	1	0.005	2	0.010					1	0.005
Annelida	*Galathowenia oculata*									1	0.005			1	0.005		
Annelida	*Gattyana amondseni*											1	0.010				
Annelida	*Glycera alba*			1	0.020			2	0.090	2	0.060	3	0.180	1	0.005	4	0.290
Annelida	*Glycera rouxii*											1	0.530				
Annelida	*Glycinde nordmanni*											1	0.020				
Annelida	*Goniada maculata*									1	0.005			1	0.030	1	0.020
Annelida	*Harmothoe* spp.											1	0.010				
Annelida	*Heteromastus filiformis*	2	0.010			2	0.005	4	0.010	4	0.005	4	0.010	1	0.005	3	0.005
Annelida	*Melinna cristata*	2	0.030														
Annelida	*Neoamphitrite affinis*	1	0.010														
Annelida	*Neoamphitrite grayi*	1	0.610														
Annelida	*Nephtys incisa*															1	0.005
Annelida	*Ophiodromus flexuosus*							1	0.040								
Annelida	*Paramphinome jeffreysii*	1	0.010	6	0.030	3	0.020			56	0.210	24	0.070	9	0.030	55	0.200
Annelida	*Phyllodoce rosea*					1	0.010										
Annelida	*Phylo norvegica*					1	0.370										
Annelida	*Pista cristata*	1	0.070	2	0.280			1	0.030								

Phylum	Species	Abu	Bio	Abu	Bio	Abu	Bio	Abu	Bio	Abu	Bio	Abu	Bio	Abu	Bio	Abu	Bio
Annelida	*Polydora* spp.													1	0.005		
Annelida	Polynoidae									2	0.005			1	0.005	1	0.005
Annelida	*Praxillella affinis*	1	0.005														
Annelida	*Prionospia dubia*	1	0.010	1	0.005												
Annelida	*Rhodine loveni*	2	0.180			1	0.030	2	0.090			1	0.020			2	0.040
Annelida	*Scalibregma inflatum*			2	0.060	1	0.020					1	0.010				
Annelida	*Scoletoma fragilis*													1	0.240		
Annelida	*Spiophanes kroeyeri*	7	0.160	7	0.100	10	0.130	5	0.050	14	0.090	9	0.040	2	0.020	15	0.160
Annelida	*Terebellides stroemi*					1	0.005										
Arthropoda	*Arrhis phyllonyx*							1	0.020			3	0.070	6	0.140	3	0.060
Arthropoda	*Campylaspis costata*					1	0.005										
Arthropoda	*Diastylis boecki*			1	0.010												
Arthropoda	*Diastyloides serratus*					6	0.010					1	0.005				
Arthropoda	*Eudorella emarginata*							1	0.005	3	0.005			3	0.020	2	0.005
Arthropoda	*Gnathia oxyurea*			1	0.005												
Arthropoda	*Leptostylis longimana*					1	0.005										
Arthropoda	*Leucon nasica*					1	0.005	1	0.005	2	0.005			2	0.010	2	0.005
Arthropoda	Lysianassidae									1	0.005						
Arthropoda	*Monoculodes carinatus*									1	0.005						
Arthropoda	*Philomedes brenda*	2	0.005			4	0.010					1	0.005				
Arthropoda	Tanaidacea	2	0.005			1	0.005										
Echinodermata	*Amphiura chiajei*							1	0.008								
Echinodermata	*Amphiura filiformis*					1	0.005	1	0.008								
Echinodermata	*Brissopsis lyrifera*							1	12.110								
Echinodermata	*Ophiocten affinis*					1	0.080										
Mollusca	*Abra nitida*	1	0.005	1	0.010	3	0.005	13	0.550	2	0.080	9	0.230	13	0.380	13	0.370
Mollusca	*Corbula gibba*	1	0.010														
Mollusca	*Ennucula tenuis*	1	0.005														
Mollusca	*Hyala vitrea*	3	0.010														
Mollusca	*Mysella bidentata*	1	0.005														
Mollusca	Nudibranchia									1	0.020						
Mollusca	*Parvicardium pinnulatum*							1	0.020								
Mollusca	*Philine scabra*							3	0.050	3	0.030	4	0.060	1	0.020	1	0.030
Mollusca	*Thyasira equalis*	22	0.430	28	0.340	18	0.180	1	0.050	17	0.500	27	0.540	3	0.090	12	0.390
Mollusca	*Thyasira sarsii*					1	0.005										
Mollusca	*Yoldiella philippiana*	10	0.130	5	0.040	12	0.170	1	0.020	4	0.060	4	0.030	2	0.050		
Nemertea	*Cerebratulus* spp.							1	0.040								
Platyhelminthes	Turbellaria											1	0.030				
Sipuncula	*Phascolion strombus*	6	0.005	5	0.005	1	0.005										

f. Ref-80

Phylum	Species	Ref-80:1A		Ref-80:1B		Ref-80:1C		Ref-80:14A		Ref-80:14B		Ref-80:14C		Ref-80:14D		Ref-80:14E	
		Abu	Bio	Abu	Bio	Abu	Bio	Abu	Bio	Abu	Bio	Abu	Bio	Abu	Bio	Abu	Bio
Annelida	*Abyssoninoe hibernica*	3	0.120	4	0.130	2	0.070	3	0.070	2	0.030	5	0.160	6	0.420	2	0.080
Annelida	*Anobothrus gracilis*											1	0.010			1	0.005
Annelida	*Aphelochaeta marioni*	4	0.040	2	0.020	4	0.040	2	0.010	9	0.090	15	0.130	20	0.250	11	0.150
Annelida	*Brada villosa*									2	0.010	1	0.005	2	0.070	6	0.180
Annelida	*Bylgides elegans*									1	0.005						
Annelida	*Ceratocephale loveni*					3	0.030	4	0.050	2	0.040	4	0.040	3	0.040	2	0.005
Annelida	*Chaetozone setosa*	1	0.010			1	0.005	13	0.070	1	0.005	12	0.050	7	0.040	11	0.050
Annelida	*Diplocirrus glaucus*	2	0.020	3	0.010	1	0.010	1	0.010	5	0.030	1	0.010	1	0.010	3	0.010
Annelida	*Eclysippe eliasoni*	2	0.005			1	0.010	1	0.005	2	0.010	3	0.005	1	0.005	1	0.005
Annelida	*Euchone papillosa*			1	0.005	1	0.020	2	0.005							1	0.005
Annelida	*Eumida bahusiensis*													1	0.005		
Annelida	*Galathowenia oculata*					1	0.005					3	0.020				
Annelida	*Gattyana amondseni*			1	0.005												
Annelida	*Glycera alba*	1	0.020			4	0.160	2	0.030	3	0.080	4	0.120	3	0.060	3	0.020
Annelida	*Glycera rouxii*	2	0.380							2	0.470	1	1.080				
Annelida	*Glycinde nordmanni*					1	0.010										
Annelida	*Glyphohesione klatti*			1	0.010												
Annelida	*Goniada maculata*							1	0.040	1	0.050			2	0.070	1	0.010
Annelida	*Harmothoe* spp.							1	0.005								
Annelida	*Heteromastus filiformis*	4	0.020	3	0.010	1	0.005	7	0.020	11	0.030	9	0.020	5	0.010	8	0.020
Annelida	*Iphitime hartmanae*	1	0.080														

Phylum	Species	Abu	Bio	Abu	Bio	Abu	Bio	Abu	Bio	Abu	Bio	Abu	Bio	Abu	Bio	Abu	Bio
Annelida	*Lipobranchius jeffreysii*			2	1.080			6	1.640	2	0.530	5	1.400	3	1.620	5	1.200
Annelida	*Maldane sarsi*			3	0.010												
Annelida	*Melinna cristata*	3	0.030					1	0.030								
Annelida	*Neoamphitrite affinis*					1	2.360							4	4.320		
Annelida	*Nereiphylla lutea*	1	0.010									1	0.005				
Annelida	*Notomastus latericeus*	1	0.060	1	0.030												
Annelida	*Ophelina norvegica*															1	0.020
Annelida	*Ophiodromus flexuosus*									1	0.005	2	0.040			1	0.010
Annelida	*Paramphinome jeffreysii*			3	0.020	3	0.010	13	0.040	33	0.130	14	0.050	46	0.150	20	0.060
Annelida	*Paramphitrite tetrabranchia*	1	0.030	1	0.010	1	0.040					3	0.090			3	0.050
Annelida	*Pectinaria belgica*			1	1.670												
Annelida	*Pectinaria koreni*									1	0.010			1	0.040		
Annelida	*Pholoe baltica*							1	0.005	1	0.005						
Annelida	*Pholoe pallida*							1	0.005								
Annelida	*Phyllodoce rosea*			1	0.005												
Annelida	Phyllodocidae											1	0.005				
Annelida	*Pista cristata*	3	1.040			3	0.030							1	0.120	1	0.030
Annelida	*Polycirrus* spp.	1	0.100														
Annelida	*Polydora* spp.															1	0.005
Annelida	Polynoidae	1	0.005	1	0.005			2	0.010			1	0.005				
Annelida	*Praxillella affinis*	1	0.005	1	0.005												
Annelida	*Prionospia dubia*			2	0.020												
Annelida	*Prionospio cirrifera*							1	0.005	1	0.005	1	0.005				
Annelida	*Prionospio fallax*							4	0.020								
Annelida	*Proclea graffii*	1	0.005														
Annelida	*Rhodine loveni*			1	0.090	1	0.010	3	0.240	2	0.380	4	0.080			1	0.060
Annelida	*Scalibregma inflatum*							2	0.120	3	0.010	5	0.030	4	0.010	2	0.020
Annelida	*Scoletoma fragilis*			1	0.520												
Annelida	*Sosane sulcata*	1	0.010			1	0.005										
Annelida	*Spiophanes kroeyeri*	17	0.100	9	0.070	22	0.140	11	0.050	3	0.040	12	0.050	17	0.060	25	0.080
Annelida	*Streblosoma bairdi*	8	2.600	6	2.340	5	0.730	6	3.350	3	0.370	13	3.070	4	0.280	5	0.880
Annelida	*Terebellides stroemi*			1	0.005			1	0.040			1	0.020				
Annelida	*Tharyx killariensis*			1	0.010												
Annelida	*Trichobranchus roseus*															1	0.010
Arthropoda	*Ampelisca gibba*	1	0.010	2	0.005	1	0.005										
Arthropoda	*Ampelisca macrocephala*									3	0.010						
Arthropoda	*Aora gracilis*															2	0.005
Arthropoda	*Arrhis phyllonyx*													2	0.050		
Arthropoda	*Callianassa subterranea*											1	0.005				
Arthropoda	*Campylaspis costata*			1	0.005												
Arthropoda	*Diastylis boecki*							1	0.005							1	0.005
Arthropoda	*Diastyloides biplicatus*													1	0.005		
Arthropoda	*Diastyloides serratus*															1	0.005
Arthropoda	*Eriopisa elongata*									2	0.030						
Arthropoda	*Gnathia oxyurea*			2	0.005												
Arthropoda	*Harpinia antennaria*					1	0.005										
Arthropoda	*Harpinia crenulata*	1	0.005														
Arthropoda	*Leucothoe lilljeborgii*					1	0.005							2	0.005		
Arthropoda	Lysianassidae			1	0.020												
Arthropoda	*Westwoodilla caecula*					1	0.010					1	0.010				
Echinodermata	*Amphiura chiajei*	3	0.010	1	0.005			2	0.130	1	0.010			4	0.170	5	0.360
Echinodermata	*Amphiura filiformis*	2	0.010	1	0.005	2	0.005										
Echinodermata	*Brissopsis lyrifera*	4	16.080			1	12.460	1	3.980			1	4.450				
Echinodermata	*Echinocardium flavescens*			2	0.005												
Echinodermata	*Luidia sarsi*	1	0.005	1	0.005												
Echinodermata	Spatangidae					1	0.005										
Mollusca	*Abra nitida*	2	0.010	13	0.020	1	0.010			4	0.080	1	0.010	3	0.090	1	0.005
Mollusca	*Chaetoderma nitidulum*					1	0.005			1	0.010						
Mollusca	*Cuspidaria* spp.			1	0.010												
Mollusca	*Ennucula tenuis*	7	0.230	3	0.210	1	0.010	2	0.130			1	0.190	1	0.005	3	0.210
Mollusca	*Hyala vitrea*			1	0.005												
Mollusca	Nudibranchia							1	0.010								
Mollusca	*Philine aperta*			2	0.010												
Mollusca	*Philine scabra*	1	0.010					1	0.090			1	0.080			1	0.010
Mollusca	*Pseudamussium peslutrae*	1	0.020					1	2.560			2	3.000				
Mollusca	*Tellimya tenella*	2	0.010														

Phylum	Species	Abu	Bio	Abu	Bio	Abu	Bio	Abu	Bio	Abu	Bio	Abu	Bio	Abu	Bio	Abu	Bio
Mollusca	*Thyasira equalis*	21	0.180	36	0.150	23	0.110	10	0.130	8	0.090	13	0.300	21	0.330	18	0.260
Mollusca	*Yoldiella philippiana*	4	0.010	11	0.030	14	0.100	5	0.040	1	0.005	1	0.005	5	0.040	8	0.070
Nemertea	*Cerebratulus* spp.							1	0.020			2	0.410	2	0.460	2	0.080
Nemertea	Nemertea			1	0.005												
Platyhelminthes	Turbellaria													1	0.010		
Sipuncula	*Phascolion strombus*					1	0.005										

g. Ref-95

Phylum	Species	Ref-95:14A		Ref-95:14B		Ref-95:14C		Ref-95:14D		Ref-95:14E	
		Abu	Bio	Abu	Bio	Abu	Bio	Abu	Bio	Abu	Bio
Annelida	*Anobothrus gracilis*					1	0.005				
Annelida	*Aphelochaeta marioni*	11	0.15	12	0.19	9	0.16	20	0.25	17	0.36
Annelida	*Bylgides elegans*	1	0.03					2	0.01		
Annelida	*Ceratocephale loveni*	2	0.05	14	0.28	2	0.04	8	0.06	5	0.04
Annelida	*Chaetoparia nilssoni*	1	0.005								
Annelida	*Chaetozone setosa*	30	0.2	31	0.2	20	0.17	48	0.25	28	0.23
Annelida	*Diplocirrus glaucus*			1	0.01						
Annelida	*Euchone papillosa*							1	0.005		
Annelida	*Galathowenia oculata*							1	0.005	4	0.01
Annelida	*Glycera alba*	3	0.05					2	0.02	3	0.24
Annelida	*Glycera rouxii*	1	0.18					1	0.13	1	1.46
Annelida	*Goniada maculata*	2	0.05	2	0.11	2	0.05	1	0.005	1	0.005
Annelida	*Heteromastus filiformis*	9	0.04	3	0.01	17	0.04	3	0.01	11	0.03
Annelida	*Lipobranchius jeffreysii*					1	0.09				
Annelida	*Melinna cristata*	1	0.005	1	0.07			1	0.02	1	0.005
Annelida	*Nereimyra punctata*							1	0.005		
Annelida	*Ophelina norvegica*					1	0.06				
Annelida	*Paramphinome jeffreysii*	27	0.08	13	0.03	2	0.005	5	0.01	20	0.06
Annelida	*Pectinaria koreni*	1	0.005	2	0.01						
Annelida	*Phylo norvegica*			1	0.06						
Annelida	*Pista cristata*							3	0.56		
Annelida	*Polydora* spp.									1	0.005
Annelida	Polynoidae			1	0.01					2	0.005
Annelida	*Prionospio cirrifera*			2	0.01	4	0.02	7	0.01	2	0.005
Annelida	*Rhodine loveni*					1	0.06	2	0.17	2	0.15
Annelida	*Scalibregma inflatum*	1	0.03	5	0.2	8	0.16	4	0.13	5	0.17
Annelida	*Sige fusigera*			1	0.01			2	0.005		
Annelida	*Spiophanes kroeyeri*	35	0.33	16	0.31	10	0.15	7	0.13	8	0.12
Annelida	*Streblosoma bairdi*							2	0.22		
Annelida	*Terebellides stroemi*	2	0.03							1	0.04
Arthropoda	*Arrhis phyllonyx*	7	0.14	1	0.01	5	0.11	3	0.08	1	0.01
Arthropoda	*Callianassa subterranea*			1	0.01						
Arthropoda	*Campylaspis costata*			1	0.01						
Arthropoda	*Diastyloides serratus*	2	0.005	1	0.01						
Arthropoda	*Eriopisa elongata*			1	0.01			2	0.01	1	0.005
Arthropoda	*Eudorella emarginata*	6	0.005	2	0.01			2	0.005	3	0.005
Arthropoda	*Leucon nasica*	5	0.005	3	0.01	3	0.01	5	0.02	2	0.005
Arthropoda	*Leucothoe lilljeborgii*			1	0.01						
Arthropoda	Lysianassidae					1	0.005				
Arthropoda	*Phoxocephalidae*					1	0.005				
Arthropoda	*Westwoodilla caecula*	1	0.005								
Echinodermata	*Brissopsis lyrifera*							1	19.84		
Echinodermata	*Ophiocten affinis*					2	0.03				
Mollusca	*Abra nitida*	2	0.04	5	0.17	3	0.05	3	0.05	9	0.19
Mollusca	*Ennucula tenuis*	1	0.04								
Mollusca	*Philine scabra*					1	0.01	3	0.16	2	0.11
Mollusca	*Pseudamussium peslutrae*							1	0.72		
Mollusca	*Thyasira equalis*	3	0.08	23	0.18	1	0.03	18	0.27	23	0.46
Mollusca	*Yoldiella philippiana*	1	0.005			2	0.03			4	0.05
Nemertea	*Cerebratulus* spp.			2	0.08	3	0.09			1	0.05

Table S2. Species classification of feeding guild and taxonomic group

Feeding guild classification	Species	Phylum	Class	Order	Family
Carnivore	*Abyssoninoe hibernica*	Annelida	Polychaeta	Eunicida	Lumbrineriidae
Carnivore	*Antalis entalis*	Mollusca	Scaphopoda	Dentaliida	Dentaliidae
Carnivore	Anthozoa	Cnidaria			
Carnivore	*Bylgides elegans*	Annelida	Polychaeta	Phyllodocida	Polynoidae
Carnivore	*Campylaspis costata*	Arthropoda	Malacostraca	Cumacea	Nannastacidae
Carnivore	*Cerebratulus* spp.	Nemertea	Anopla	Heteronemertea	Lineidae
Carnivore	*Cerianthus lloydii*	Cnidaria	Hexacorallia	Ceriantharia	Cerianthidae
Carnivore	*Chaetoderma nitidulum*	Mollusca	Caudofoveata	Chaetodermatida	Chaetodermatidae
Carnivore	*Chaetoparia nilssoni*	Annelida	Polychaeta	Phyllodocida	Phyllodocidae
Carnivore	*Cuspidaria obesa*	Mollusca	Bivalvia	Anomalodesmata	Cuspidariidae
Carnivore	*Cuspidaria* spp.	Mollusca	Bivalvia	Anomalodesmata	Cuspidariidae
Carnivore	*Cylichna cylindracea*	Mollusca	Gastropoda	Cephalaspida	Cylichnidae
Carnivore	Edwardsiidae	Cnidaria	Hexacorallia	Actiniarida	Edwardsiidae
Carnivore	*Eumida bahusiensis*	Annelida	Polychaeta	Phyllodocida	Phyllodocidae
Carnivore	*Eunoe nodosa*	Annelida	Polychaeta	Phyllodocida	Polynoidae
Carnivore	*Gattyana amondseni*	Annelida	Polychaeta	Phyllodocida	Polynoidae
Carnivore	*Gattyana cirrhosa*	Annelida	Polychaeta	Phyllodocida	Polynoidae
Carnivore	*Glycera alba*	Annelida	Polychaeta	Phyllodocida	Glyceridae
Carnivore	*Glycera rouxii*	Annelida	Polychaeta	Phyllodocida	Glyceridae
Carnivore	*Glycinde nordmanni*	Annelida	Polychaeta	Phyllodocida	Goniadidae
Carnivore	*Glyphohesione klatti*	Annelida	Polychaeta	Phyllodocida	Pilargiidae
Carnivore	*Gnathia oxyurea*	Arthropoda	Malacostraca	Isopoda	Gnathiidae
Carnivore	*Goniada maculata*	Annelida	Polychaeta	Phyllodocida	Goniadidae
Carnivore	*Harmothoe* spp.	Annelida	Polychaeta	Phyllodocida	Polynoidae
Carnivore	*Iphitime hartmanae*	Annelida	Polychaeta	Eunicida	Dorvilleidae
Carnivore	*Luidia sarsi*	Echinodermata	Asteroidea	Paxillosida	Luidiidae
Carnivore	Lysianassidae	Arthropoda	Malacostraca	Amphipoda	Lysianassidae
Carnivore	Nemertea	Nemertea			
Carnivore	*Nephtys incisa*	Annelida	Polychaeta	Phyllodocida	Nephtyidae
Carnivore	*Nereimyra punctata*	Annelida	Polychaeta	Phyllodocida	Hesionidae
Carnivore	*Nereiphylla lutea*	Annelida	Polychaeta	Phyllodocida	Phyllodocidae
Carnivore	Nudibranchia	Mollusca	Gastropoda	Nudibranchia	
Carnivore	*Ophiocten affinis*	Echinodermata	Ophiuroidea	Ophiurida	Ophiuridae
Carnivore	*Ophiodromus flexuosus*	Annelida	Polychaeta	Phyllodocida	Hesionidae
Carnivore	*Pagurus bernhardus*	Arthropoda	Malacostraca	Decapoda	Paguridae
Carnivore	*Paramphinome jeffreysii*	Annelida	Polychaeta	Eunicida	Amphinomidae
Carnivore	*Philine aperta*	Mollusca	Gastropoda	Cephalaspida	Philinidae
Carnivore	*Philine scabra*	Mollusca	Gastropoda	Cephalaspida	Philinidae
Carnivore	*Pholoe baltica*	Annelida	Polychaeta	Phyllodocida	Pholoidae
Carnivore	*Pholoe pallida*	Annelida	Polychaeta	Phyllodocida	Pholoidae
Carnivore	*Phyllodoce groenlandica*	Annelida	Polychaeta	Phyllodocida	Phyllodocidae
Carnivore	*Phyllodoce rosea*	Annelida	Polychaeta	Phyllodocida	Phyllodocidae
Carnivore	Phyllodocidae	Annelida	Polychaeta	Phyllodocida	Phyllodocidae
Carnivore	*Pilargis verrucosa*	Annelida	Polychaeta	Phyllodocida	Pilargiidae
Carnivore	*Podarkeopsis helgolandicus*	Annelida	Polychaeta	Phyllodocida	Hesionidae
Carnivore	*Polinices montagui*	Mollusca	Gastropoda	Mesogastropoda	Naticidae
Carnivore	*Polinices pulchella*	Mollusca	Gastropoda	Mesogastropoda	Naticidae
Carnivore	Polynoidae	Annelida	Polychaeta	Phyllodocida	Polynoidae
Carnivore	*Psamathe fusca*	Annelida	Polychaeta	Phyllodocida	Hesionidae
Carnivore	*Scoletoma fragilis*	Annelida	Polychaeta	Eunicida	Lumbrineriidae
Carnivore	*Sige fusigera*	Annelida	Polychaeta	Phyllodocida	Phyllodocidae
Carnivore	Turbellaria	Platyhelminthes			
Filter feeder	*Chaetopterus norvegicus*	Annelida	Polychaeta	Spionida	Chaetopteridae
Filter feeder	*Corbula gibba*	Mollusca	Bivalvia	Heterodonta	Corbulidae
Filter feeder	*Myrtea spinifera*	Mollusca	Bivalvia	Heterodonta	Lucinidae
Filter feeder	*Mysia undata*	Mollusca	Bivalvia	Heterodonta	Petricolidae
Filter feeder	*Parvicardium minimum*	Mollusca	Bivalvia	Heterodonta	Cardiidae
Filter feeder	*Parvicardium pinnulatum*	Mollusca	Bivalvia	Heterodonta	Cardiidae
Filter feeder	*Phaxas pellucida*	Mollusca	Bivalvia	Heterodonta	Pharidae
Filter feeder	*Pseudamussium peslutrae*	Mollusca	Bivalvia	Pteriomorpha	Pectinidae
Suspension feeder	*Amphiura filiformis*	Echinodermata	Ophiuroidea	Ophiurida	Amphiuridae
Suspension feeder	*Euchone papillosa*	Annelida	Polychaeta	Sabellida	Sabellariidae
Suspension feeder	*Leptopentacta elongata*	Echinodermata	Holothuroidea	Dendrochirotida	Cucumariidae
Suspension feeder	*Mysella bidentata*	Mollusca	Bivalvia	Heterodonta	Montacutidae

Feeding guild classification	Species	Phylum	Class	Order	Family
Suspension feeder	*Phascolion strombus*	Sipuncula	Sipunculidea	Golfingiiformes	Phascolionidae
Suspension feeder	Sabellidae	Annelida	Polychaeta	Sabellida	Sabellidae
Deposit feeder	*Abra nitida*	Mollusca	Bivalvia	Heterodonta	Semelidae
Deposit feeder	*Ampelisca gibba*	Arthropoda	Malacostraca	Amphipoda	Ampeliscidae
Deposit feeder	*Ampelisca macrocephala*	Arthropoda	Malacostraca	Amphipoda	Ampeliscidae
Deposit feeder	*Ampharete baltica*	Annelida	Polychaeta	Terebellida	Ampharetidae
Deposit feeder	*Ampharete finmarchica*	Annelida	Polychaeta	Terebellida	Ampharetidae
Deposit feeder	*Amphiura chiajei*	Echinodermata	Ophiuroidea	Ophiurida	Amphiuridae
Deposit feeder	*Anobothrus gracilis*	Annelida	Polychaeta	Terebellida	Ampharetidae
Deposit feeder	*Aora gracilis*	Arthropoda	Malacostraca	Amphipoda	Aoridae
Deposit feeder	*Aphelochaeta marioni*	Annelida	Polychaeta	Terebellida	Cirratulidae
Deposit feeder	*Aphelochaeta* sp.	Annelida	Polychaeta	Terebellida	Cirratulidae
Deposit feeder	*Arrhis phyllonyx*	Arthropoda	Malacostraca	Amphipoda	Oedicerotidae
Deposit feeder	*Brada villosa*	Annelida	Polychaeta	Terebellida	Flabelligeridae
Deposit feeder	*Brissopsis lyrifera*	Echinodermata	Echinoidea	Spatangoida	Brissidae
Deposit feeder	*Chaetozone setosa*	Annelida	Polychaeta	Terebellida	Cirratulidae
Deposit feeder	*Cossura longocirrata*	Annelida	Polychaeta		Cossuridae
Deposit feeder	*Diastylis boecki*	Arthropoda	Malacostraca	Cumacea	Diastylidae
Deposit feeder	*Diastylis laevis*	Arthropoda	Malacostraca	Cumacea	Diastylidae
Deposit feeder	*Diastyloides biplicatus*	Arthropoda	Malacostraca	Cumacea	Diastylidae
Deposit feeder	*Diastyloides serratus*	Arthropoda	Malacostraca	Cumacea	Diastylidae
Deposit feeder	*Diplocirrus glaucus*	Annelida	Polychaeta	Terebellida	Flabelligeridae
Deposit feeder	*Echinocardium cordatum*	Echinodermata	Echinoidea	Spatangoida	Loveniidae
Deposit feeder	*Echinocardium flavescens*	Echinodermata	Echinoidea	Spatangoida	Loveniidae
Deposit feeder	*Eclysippe eliasoni*	Annelida	Polychaeta	Terebellida	Ampharetidae
Deposit feeder	*Eudorella emarginata*	Arthropoda	Malacostraca	Cumacea	Leuconidae
Deposit feeder	*Galathowenia oculata*	Annelida	Polychaeta	Sabellida	Oweniidae
Deposit feeder	*Golfingia vulgaris*	Sipuncula	Sipunculidea	Golfingiiformes	Golfingiidae
Deposit feeder	*Harpinia antennaria*	Arthropoda	Malacostraca	Amphipoda	Phoxocephalidae
Deposit feeder	*Harpinia crenulata*	Arthropoda	Malacostraca	Amphipoda	Phoxocephalidae
Deposit feeder	*Labidoplax buskii*	Echinodermata	Holothuroidea	Apodida	Synaptidae
Deposit feeder	*Laonice bahusiensis*	Annelida	Polychaeta	Spionida	Spionidae
Deposit feeder	*Leptostylis longimana*	Arthropoda	Malacostraca	Cumacea	Diastylidae
Deposit feeder	*Leucon nasica*	Arthropoda	Malacostraca	Cumacea	Leuconidae
Deposit feeder	*Leucothoe lilljeborgii*	Arthropoda	Malacostraca	Amphipoda	Leucothoidae
Deposit feeder	*Magelona minuta*	Annelida	Polychaeta	Spionida	Magelonidae
Deposit feeder	*Maldane sarsi*	Annelida	Polychaeta		Maldanidae
Deposit feeder	*Melinna cristata*	Annelida	Polychaeta	Terebellida	Ampharetidae
Deposit feeder	*Mesothuria intestinalis*	Echinodermata	Holothuroidea	Aspidochirotida	Synallactidae
Deposit feeder	*Monoculodes carinatus*	Arthropoda	Malacostraca	Amphipoda	Oedicerotidae
Deposit feeder	*Montacuta ferruginosa*	Mollusca	Bivalvia	Heterodonta	Montacutidae
Deposit feeder	*Neoamphitrite affinis*	Annelida	Polychaeta	Terebellida	Terebellidae
Deposit feeder	*Neoamphitrite grayi*	Annelida	Polychaeta	Terebellida	Terebellidae
Deposit feeder	Oedicerotidae	Arthropoda	Malacostraca	Amphipoda	Oedicerotidae
Deposit feeder	*Owenia fusiformis*	Annelida	Polychaeta	Sabellida	Oweniidae
Deposit feeder	*Paramphitrite tetrabranchia*	Annelida	Polychaeta	Terebellida	Terebellidae
Deposit feeder	*Philomedes brenda*	Arthropoda	Ostracoda	Myodocopida	Philomedidae
Deposit feeder	Phoxocephalidae	Arthropoda	Malacostraca	Amphipoda	Phoxocephalidae
Deposit feeder	*Pista cristata*	Annelida	Polychaeta	Terebellida	Terebellidae
Deposit feeder	*Polycirrus* spp.	Annelida	Polychaeta	Terebellida	Terebellidae
Deposit feeder	*Polydora* spp.	Annelida	Polychaeta	Spionida	Spionidae
Deposit feeder	*Prionospia dubia*	Annelida	Polychaeta	Spionida	Spionidae
Deposit feeder	*Prionospio cirrifera*	Annelida	Polychaeta	Spionida	Spionidae
Deposit feeder	*Prionospio fallax*	Annelida	Polychaeta	Spionida	Spionidae
Deposit feeder	*Prionospio multibranchiata*	Annelida	Polychaeta	Spionida	Spionidae
Deposit feeder	*Proclea graffii*	Annelida	Polychaeta	Terebellida	Terebellidae
Deposit feeder	*Scolelepis tridentata*	Annelida	Polychaeta	Spionida	Spionidae
Deposit feeder	*Sosane sulcata*	Annelida	Polychaeta	Terebellida	Ampharetidae
Deposit feeder	Spatangidae	Echinodermata	Echinoidea	Spatangoida	Spatangidae
Deposit feeder	*Spiophanes kroeyeri*	Annelida	Polychaeta	Spionida	Spionidae
Deposit feeder	*Streblosoma bairdi*	Annelida	Polychaeta	Terebellida	Terebellidae
Deposit feeder	Tanaidacea	Arthropoda	Malacostraca	Tanaidacea	
Deposit feeder	*Tellimya tenella*	Mollusca	Bivalvia	Heterodonta	Montacutidae
Deposit feeder	*Terebellides stroemi*	Annelida	Polychaeta	Terebellida	Terebellidae
Deposit feeder	*Tharyx killariensis*	Annelida	Polychaeta	Terebellida	Cirratulidae
Deposit feeder	*Thysanocardia procera*	Sipuncula	Sipunculidea	Golfingiiformes	Golfingiidae

Feeding guild classification	Species	Phylum	Class	Order	Family
Deposit feeder	*Trichobranchus roseus*	Annelida	Polychaeta	Terebellida	Terebellidae
Deposit feeder	*Westwoodilla caecula*	Arthropoda	Malacostraca	Amphipoda	Oedicerotidae
Deposit feeder	*Yoldiella philippiana*	Mollusca	Bivalvia	Nuculoida	Yoldiidae
Subsurface deposit feeder	*Callianassa subterranea*	Arthropoda	Malacostraca	Decapoda	Callianassidae
Subsurface deposit feeder	*Ceratocephale loveni*	Annelida	Polychaeta	Phyllodocida	Nereididae
Subsurface deposit feeder	*Ennucula tenuis*	Mollusca	Bivalvia	Nuculoida	Nuculidae
Subsurface deposit feeder	*Eriopisa elongata*	Arthropoda	Malacostraca	Amphipoda	Melitidae
Subsurface deposit feeder	*Heteromastus filiformis*	Annelida	Polychaeta		Capitellidae
Subsurface deposit feeder	*Hyala vitrea*	Mollusca	Gastropoda	Mesogastropoda	Iravadiidae
Subsurface deposit feeder	*Lipobranchius jeffreysii*	Annelida	Polychaeta		Scalibregmatidae
Subsurface deposit feeder	*Lumbriclymene minor*	Annelida	Polychaeta		Maldanidae
Subsurface deposit feeder	Maldanidae	Annelida	Polychaeta		Maldanidae
Subsurface deposit feeder	*Notomastus latericeus*	Annelida	Polychaeta		Capitellidae
Subsurface deposit feeder	*Nucula nitidosa*	Mollusca	Bivalvia	Nuculoida	Nuculidae
Subsurface deposit feeder	*Nucula sulcata*	Mollusca	Bivalvia	Nuculoida	Nuculidae
Subsurface deposit feeder	*Ophelina norvegica*	Annelida	Polychaeta		Opheliidae
Subsurface deposit feeder	*Pectinaria auricoma*	Annelida	Polychaeta	Terebellida	Pectinaridae
Subsurface deposit feeder	*Pectinaria belgica*	Annelida	Polychaeta	Terebellida	Pectinaridae
Subsurface deposit feeder	*Pectinaria koreni*	Annelida	Polychaeta	Terebellida	Pectinaridae
Subsurface deposit feeder	*Phylo norvegica*	Annelida	Polychaeta		Orbiniidae
Subsurface deposit feeder	*Polyphysia crassa*	Annelida	Polychaeta		Scalibregmatidae
Subsurface deposit feeder	*Praxillella affinis*	Annelida	Polychaeta		Maldanidae
Subsurface deposit feeder	*Praxillella praetermissa*	Annelida	Polychaeta		Maldanidae
Subsurface deposit feeder	*Rhodine loveni*	Annelida	Polychaeta		Maldanidae
Subsurface deposit feeder	*Scalibregma inflatum*	Annelida	Polychaeta		Scalibregmatidae
Subsurface deposit feeder	*Thyasira equalis*	Mollusca	Bivalvia	Heterodonta	Thyasiridae
Subsurface deposit feeder	*Thyasira flexuosa*	Mollusca	Bivalvia	Heterodonta	Thyasiridae
Subsurface deposit feeder	*Thyasira sarsii*	Mollusca	Bivalvia	Heterodonta	Thyasiridae

Table S3. SIMPER-analysis from 30 m.

SIMPER Similarity Percentages - species contributions

One-Way Analysis

Data type: Abundance
Variable selection: All

Parameters
Transform: Fourth root
Resemblance: S17 Bray Curtis similarity
Cut off for low contributions: 90%

Factor Groups

Sample	sample unit
AC+clay-30:1A	AC+clay-30:1
AC+clay-30:1B	AC+clay-30:1
AC+clay-30:1C	AC+clay-30:1
Clay-30:1A	Clay-30:1
Clay-30:1B	Clay-30:1
Clay-30:1C	Clay-30:1
Lime-30:1A	Lime-30:1
Lime-30:1B	Lime-30:1
Lime-30:1C	Lime-30:1
Ref-30:1A	Ref-30:1
Ref-30:1B	Ref-30:1
Ref-30:1C	Ref-30:1
AC+clay-30:14A	AC+clay-30:14
AC+clay-30:14B	AC+clay-30:14
AC+clay-30:14C	AC+clay-30:14
AC+clay-30:14D	AC+clay-30:14
AC+clay-30:14E	AC+clay-30:14
Clay-30:14A	Clay-30:14
Clay-30:14B	Clay-30:14
Clay-30:14C	Clay-30:14
Clay-30:14D	Clay-30:14
Clay-30:14E	Clay-30:14
Lime-30:14A	Lime-30:14
Lime-30:14B	Lime-30:14
Lime-30:14C	Lime-30:14
Lime-30:14D	Lime-30:14
Lime-30:14E	Lime-30:14
Ref-30:14A	Ref-30:14
Ref-30:14B	Ref-30:14
Ref-30:14C	Ref-30:14
Ref-30:14D	Ref-30:14
Ref-30:14E	Ref-30:14

Group AC+clay-30:1
Average similarity: 61.55%

Species	Av. Abund	Av. Sim	Sim/SD	Contrib. %	Cumul. %
Hyala vitrea	2.21	7.16	6.45	11.63	11.63
Cylichna cylindracea	2.10	6.37	7.87	10.34	21.98
Corbula gibba	1.89	6.02	6.45	9.78	31.76
Echinocardium cordatum	1.82	6.02	6.45	9.78	41.55
Montacuta ferruginosa	1.89	6.02	6.45	9.78	51.33
Scalibregma inflatum	1.82	6.02	6.45	9.78	61.11
Trichobranchus roseus	1.82	6.02	6.45	9.78	70.90
Nephtys incisa	1.56	2.41	0.58	3.92	74.82
Amphiura filiformis	1.14	2.37	0.58	3.84	78.66
Montacuta tenella	1.43	2.17	0.58	3.52	82.18
Abra nitida	1.25	1.83	0.58	2.98	85.16
Heteromastus filiformis	1.14	1.83	0.58	2.98	88.14
Polinices pulchella	1.14	1.83	0.58	2.98	91.12

Group Clay-30:1
Average similarity: 52.76%

Species	Av. Abund	Av. Sim	Sim/SD	Contrib. %	Cumul. %
Amphiura filiformis	3.96	8.13	10.44	15.42	15.42
Corbula gibba	3.03	6.21	8.11	11.78	27.19
Nucula nitidosa	2.72	5.05	3.61	9.56	36.76
Hyala vitrea	2.68	4.65	103.91	8.81	45.57
Abyssoninoe hibernica	2.28	4.05	15.49	7.67	53.24
Amphiura chiajei	2.06	3.95	5.72	7.48	60.72
Callianassa subterranea	1.71	3.70	10.72	7.01	67.74
Spiophanes kroeyeri	1.82	3.70	10.72	7.01	74.75
Thyasira flexuosa	1.66	1.85	0.58	3.51	78.26
Diplocirrus glaucus	1.56	1.45	0.58	2.75	81.01
Chaetoderma nitidulum	1.14	1.31	0.58	2.48	83.49
Pectinaria auricoma	1.32	1.31	0.58	2.48	85.98
Brissopsis lyrifera	1.36	1.31	0.58	2.48	88.46
Pectinaria belgica	1.36	1.31	0.58	2.48	90.94

Group Lime-30:1
Average similarity: 42.11%

Species	Av. Abund	Av. Sim	Sim/SD	Contrib. %	Cumul. %
Prionospio fallax	2.78	5.98	10.42	14.21	14.21
Amphiura filiformis	2.98	5.89	7.23	13.98	28.19
Corbula gibba	2.71	5.47	11.16	13.00	41.19
Glycera alba	2.21	4.99	4.87	11.85	53.04
Praxillella praetermissa	1.82	4.20	4.87	9.96	63.00
Tharyx killariensis	1.36	2.00	0.58	4.75	67.75
Thysanocardia procera	1.14	1.68	0.58	3.99	71.74
Hyala vitrea	1.43	1.67	0.58	3.98	75.71
Thyasira flexuosa	1.68	1.46	0.58	3.46	79.17
Nemertea	1.56	1.41	0.58	3.34	82.52
Pholoe baltica	1.14	1.41	0.58	3.34	85.86
Amphiura chiajei	1.53	1.32	0.58	3.13	88.99
Scalibregma inflatum	1.53	1.32	0.58	3.13	92.11

Group Ref-30:1
Average similarity: 57.88%

Species	Av. Abund	Av. Sim	Sim/SD	Contrib. %	Cumul. %
Amphiura filiformis	3.68	11.39	12.57	19.67	19.67
Corbula gibba	2.56	7.17	12.06	12.39	32.06
Amphiura chiajei	1.71	5.45	12.06	9.41	41.47
Brissopsis lyrifera	1.82	5.45	12.06	9.41	50.89
Callianassa subterranea	1.82	5.45	12.06	9.41	60.30
Nephtys incisa	1.71	5.45	12.06	9.41	69.72
Cylichna cylindracea	1.56	2.24	0.58	3.87	73.59
Hyala vitrea	1.36	2.02	0.58	3.50	77.08
Spiophanes kroeyeri	1.36	2.02	0.58	3.50	80.58
Abyssoninoe hibernica	1.38	1.99	0.58	3.43	84.01
Chaetoderma nitidulum	1.14	1.99	0.58	3.43	87.45
Goniada maculata	1.14	1.99	0.58	3.43	90.88

Group AC+clay-30:14
Average similarity: 11.97%

Species	Av. Abund	Av. Sim	Sim/SD	Contrib. %	Cumul. %
Thyasira sarsii	1.03	6.74	0.59	56.33	56.33
Nephtys incisa	0.81	2.84	0.32	23.72	80.05
Spiophanes kroeyeri	0.69	2.39	0.32	19.95	100.00

Group Clay-30:14
Average similarity: 49.92%

Species	Av. Abund	Av. Sim	Sim/SD	Contrib. %	Cumul. %
Scalibregma inflatum	3.76	8.47	3.88	16.97	16.97
Amphiura filiformis	3.59	8.47	8.62	16.96	33.93
Corbula gibba	2.59	6.06	7.73	12.15	46.08
Nephtys incisa	2.02	4.86	8.28	9.74	55.81
Brissopsis lyrifera	1.71	4.39	7.09	8.79	64.61
Glycera alba	1.48	2.73	1.14	5.47	70.08
Abyssoninoe hibernica	1.37	2.61	1.13	5.23	75.31
Diplocirrus glaucus	1.54	2.56	1.14	5.13	80.43
Nucula nitidosa	1.27	1.52	0.61	3.05	83.49
Thysanocardia procera	1.03	1.45	0.62	2.91	86.39
Hyala vitrea	1.14	1.21	0.62	2.42	88.81
Philine scabra	1.03	1.20	0.62	2.41	91.21

Group Lime-30:14
Average similarity: 36.15%

Species	Av. Abund	Av. Sim	Sim/SD	Contrib. %	Cumul. %
Amphiura filiformis	3.95	7.93	5.64	21.95	21.95
Corbula gibba	2.46	4.62	4.75	12.77	34.72
Scalibregma inflatum	2.29	3.70	1.08	10.24	44.97
Philine scabra	1.59	2.25	1.10	6.22	51.19
Amphiura chiajei	1.71	2.21	1.14	6.13	57.31
Polydora spp	2.09	1.52	0.60	4.21	61.52
Pectinaria auricoma	1.17	1.37	0.61	3.80	65.32
Thyasira flexuosa	1.99	1.35	0.59	3.75	69.06
Galathowenia oculata	1.69	1.16	0.58	3.21	72.27
Hyala vitrea	1.39	1.16	0.60	3.20	75.47
Anobothrus gracilis	1.17	1.13	0.61	3.12	78.59
Glycera alba	1.09	1.08	0.61	2.99	81.58
Diastylis boecki	1.03	1.00	0.62	2.75	84.33
Edwardsiidae	1.20	0.95	0.62	2.62	86.95
Diplocirrus glaucus	1.00	0.48	0.32	1.33	88.28
Pectinaria koreni	0.81	0.46	0.32	1.27	89.55
Pholoe baltica	0.88	0.44	0.32	1.21	90.76

Group Ref-30:14
Average similarity: 55.53%

Species	Av. Abund	Av. Sim	Sim/SD	Contrib. %	Cumul. %
Scalibregma inflatum	3.71	8.60	7.00	15.49	15.49
Amphiura filiformis	2.84	6.93	9.95	12.48	27.98
Diplocirrus glaucus	2.22	5.53	13.96	9.96	37.93
Spiophanes kroeyeri	2.08	5.43	16.57	9.78	47.71
Ophiodromus flexuosus	1.91	4.81	16.65	8.66	56.37
Nephtys incisa	1.78	4.57	16.57	8.22	64.59
Callianassa subterranea	1.70	3.14	1.12	5.65	70.25
Streblosoma bairdi	1.44	2.69	1.16	4.85	75.10
Abyssoninoe hibernica	1.31	1.65	0.62	2.98	78.08
Terebellides stroemi	1.45	1.53	0.60	2.75	80.83
Pholoe baltica	1.03	1.40	0.62	2.53	83.36
Thysanocardia procera	1.09	1.40	0.62	2.53	85.88
Nemertea	1.03	1.37	0.62	2.46	88.35
Glycera alba	1.09	1.34	0.62	2.42	90.77

Groups AC+clay-30:1 & Clay-30:1
Average dissimilarity = 68.94%

Species	Group AC+clay-30:1 Av. Abund	Group Clay-30:1 Av. Abund	Av. Diss	Diss/SD	Contrib. %	Cumul. %
Amphiura filiformis	1.14	3.96	3.75	3.39	5.44	5.44
Nucula nitidosa	0.00	2.72	3.70	3.82	5.36	10.80
Amphiura chiajei	0.00	2.06	2.82	3.57	4.09	14.89
Montacuta ferruginosa	1.89	0.00	2.53	8.19	3.67	18.55
Spiophanes kroeyeri	0.00	1.82	2.48	4.36	3.60	22.15
Trichobranchus roseus	1.82	0.00	2.47	5.11	3.58	25.73
Thyasira flexuosa	0.00	1.66	2.35	1.28	3.40	29.13
Abyssoninoe hibernica	0.57	2.28	2.34	1.71	3.40	32.53
Cylichna cylindracea	2.10	0.75	2.13	1.52	3.09	35.62
Pectinaria auricoma	0.00	1.32	1.89	1.22	2.74	38.36
Polyphysia crassa	0.00	1.32	1.87	1.21	2.72	41.08
Nephtys incisa	1.56	0.57	1.84	1.23	2.67	43.75
Echinocardium cordatum	1.82	0.68	1.79	1.54	2.60	46.34
Diplocirrus glaucus	0.57	1.56	1.78	1.26	2.58	48.92
Scalibregma inflatum	1.82	0.57	1.75	1.37	2.54	51.46
Thysanocardia procera	0.00	1.38	1.72	1.27	2.49	53.95
Pectinaria belgica	0.00	1.36	1.69	1.32	2.46	56.41
Montacuta tenella	1.43	0.75	1.66	1.06	2.41	58.81
Polycirrus spp	1.25	0.00	1.62	1.31	2.35	61.16
Chaetoderma nitidulum	0.00	1.14	1.61	1.30	2.33	63.50
Edwardsiidae	0.00	1.14	1.59	1.29	2.31	65.81
Corbula gibba	1.89	3.03	1.58	2.38	2.30	68.10
Callianassa subterranea	0.57	1.71	1.50	1.29	2.17	70.28
Philine scabra	0.57	1.32	1.46	1.15	2.12	72.40
Abra nitida	1.25	0.57	1.44	1.08	2.09	74.49
Luidia sarsi	1.25	0.57	1.41	1.11	2.05	76.53
Brissopsis lyrifera	1.14	1.36	1.34	1.09	1.95	78.48
Polinices pulchella	1.14	0.57	1.29	1.03	1.87	80.35
Heteromastus filiformis	1.14	0.57	1.28	1.03	1.86	82.21
Terebellides stroemi	0.00	0.75	0.95	0.66	1.37	83.58
Echinocardium flavescens	0.00	0.57	0.89	0.66	1.29	84.87
Glycera alba	0.00	0.57	0.89	0.66	1.29	86.16
Trachythyone elongata	0.00	0.57	0.89	0.66	1.29	87.45
Mysella bidentata	0.00	0.68	0.84	0.66	1.22	88.67
Hyala vitrea	2.21	2.68	0.79	0.99	1.14	89.81
Glycera rouxii	0.00	0.57	0.72	0.66	1.04	90.85

Groups Clay-30:1 & Lime-30:1
Average dissimilarity = 67.28%

Species	Group Clay-30:1 Av. Abund	Group Lime-30:1 Av. Abund	Av. Diss	Diss/SD	Contrib. %	Cumul. %
Nucula nitidosa	2.72	0.00	3.21	3.24	4.77	4.77
Prionospio fallax	0.00	2.78	3.18	9.06	4.73	9.50
Spiophanes kroeyeri	1.82	0.00	2.15	3.59	3.20	12.70
Abyssoninoe hibernica	2.28	0.57	2.08	1.61	3.09	15.79
Callianassa subterranea	1.71	0.00	2.01	4.58	2.98	18.77
Glycera alba	0.57	2.21	1.80	1.95	2.67	21.44
Nemertea	0.00	1.56	1.78	1.31	2.65	24.09
Tharyx killariensis	0.00	1.36	1.74	1.28	2.58	26.67
Pectinaria auricoma	1.32	0.00	1.63	1.18	2.42	29.09
Polyphysia crassa	1.32	0.00	1.62	1.18	2.41	31.50
Thyasira flexuosa	1.66	1.68	1.52	0.92	2.25	33.75
Brissopsis lyrifera	1.36	0.00	1.48	1.28	2.21	35.96
Pectinaria belgica	1.36	0.00	1.48	1.28	2.21	38.16
Praxillella praetermissa	0.57	1.82	1.48	1.36	2.20	40.37
Heteromastus filiformis	0.57	1.56	1.46	1.19	2.17	42.54
Scalibregma inflatum	0.57	1.53	1.45	1.26	2.16	44.70
Philine scabra	1.32	0.00	1.44	1.25	2.15	46.84
Hyala vitrea	2.68	1.43	1.43	1.10	2.12	48.97
Chaetoderma nitidulum	1.14	0.00	1.39	1.25	2.06	51.03

Pholoe baltica	0.00	1.14	1.38	1.24	2.05	53.08
Diplocirrus glaucus	1.56	1.32	1.36	1.02	2.02	55.10
Chaetozone setosa	0.00	1.32	1.35	1.31	2.01	57.10
Amphiura filiformis	3.96	2.98	1.31	1.03	1.95	59.05
Amphiura chiajei	2.06	1.53	1.26	0.88	1.87	60.92
Thysanocardia procera	1.38	1.14	1.16	1.07	1.72	62.65
Edwardsiidae	1.14	0.57	1.15	1.00	1.70	64.35
Cylichna cylindracea	0.75	0.57	1.05	0.90	1.56	65.91
Polinices pulchella	0.57	0.68	0.98	0.88	1.45	67.37
Luidia sarsi	0.57	0.57	0.86	0.83	1.28	68.65
Goniada maculata	0.00	0.57	0.83	0.66	1.24	69.88
Pilargis verrucosa	0.00	0.57	0.83	0.66	1.24	71.12
Laonice bahusiensis	0.57	0.57	0.83	0.83	1.23	72.35
Polydora spp	0.57	0.57	0.83	0.83	1.23	73.58
Terebellides stroemi	0.75	0.00	0.83	0.65	1.23	74.81
Galathowenia oculata	0.00	0.85	0.82	0.66	1.22	76.03
Montacuta tenella	0.75	0.00	0.81	0.65	1.21	77.24
Maldane sarsi	0.00	0.81	0.78	0.66	1.15	78.39
Echinocardium flavescens	0.57	0.00	0.76	0.65	1.13	79.52
Trachythyone elongata	0.57	0.00	0.76	0.65	1.13	80.65
Echinocardium cordatum	0.68	0.00	0.74	0.65	1.09	81.75
Mysella bidentata	0.68	0.00	0.74	0.65	1.09	82.84
Trichobranchus roseus	0.00	0.75	0.72	0.66	1.07	83.91
Corbula gibba	3.03	2.71	0.67	0.99	1.00	84.91
Glycera rouxii	0.57	0.00	0.63	0.65	0.94	85.85
Cerebratulus spp	0.00	0.57	0.63	0.66	0.93	86.78
Magelona minuta	0.00	0.57	0.63	0.66	0.93	87.71
Pholoe pallida	0.00	0.57	0.63	0.66	0.93	88.65
Polinices montagui	0.00	0.57	0.63	0.66	0.93	89.58
Prionospio multibranchiata	0.00	0.57	0.63	0.66	0.93	90.51

Groups AC+clay-30:1 & Ref-30:1
Average dissimilarity = 61.69%

Species	Group AC+clay-30:1 Av. Abund	Group Ref-30:1 Av. Abund	Av. Diss	Diss/SD	Contrib. %	Cumul. %
Amphiura filiformis	1.14	3.68	4.13	4.44	6.70	6.70
Montacuta ferruginosa	1.89	0.00	3.14	13.48	5.09	11.79
Trichobranchus roseus	1.82	0.00	3.08	5.31	4.99	16.78
Scalibregma inflatum	1.82	0.00	3.07	5.40	4.98	21.76
Amphiura chiajei	0.00	1.71	2.88	7.59	4.67	26.43
Montacuta tenella	1.43	0.00	2.33	1.28	3.77	30.20
Spiophanes kroeyeri	0.00	1.36	2.21	1.31	3.58	33.78
Abyssoninoe hibernica	0.57	1.38	2.12	1.09	3.43	37.21
Thysanocardia procera	0.00	1.25	2.06	1.31	3.34	40.55
Callianassa subterranea	0.57	1.82	2.05	1.39	3.32	43.87
Echinocardium cordatum	1.82	0.57	2.01	1.43	3.26	47.13
Chaetoderma nitidulum	0.00	1.14	2.01	1.31	3.26	50.38
Goniada maculata	0.00	1.14	2.01	1.31	3.26	53.64
Heteromastus filiformis	1.14	0.00	1.85	1.30	3.00	56.64
Polinices pulchella	1.14	0.00	1.85	1.30	3.00	59.65
Luidia sarsi	1.25	0.57	1.75	1.11	2.84	62.49
Polycirrus spp	1.25	0.57	1.75	1.11	2.84	65.33
Nephtys incisa	1.56	1.71	1.71	1.62	2.78	68.11
Cylichna cylindracea	2.10	1.56	1.68	1.00	2.72	70.83
Diplocirrus glaucus	0.57	1.14	1.63	1.04	2.65	73.48
Abra nitida	1.25	1.14	1.50	0.96	2.44	75.92
Philine scabra	0.57	0.68	1.50	0.90	2.43	78.34
Hyala vitrea	2.21	1.36	1.47	0.79	2.38	80.73
Brissopsis lyrifera	1.14	1.82	1.22	0.76	1.98	82.70
Eriopisa elongata	0.00	0.75	1.15	0.66	1.86	84.56
Corbula gibba	1.89	2.56	1.13	1.32	1.83	86.39
Nucula nitidosa	0.00	0.68	1.04	0.66	1.68	88.07
Praxillella praetermissa	0.00	0.68	1.04	0.66	1.68	89.75
Terebellides stroemi	0.00	0.57	1.02	0.66	1.66	91.41

Groups Clay-30:1 & Ref-30:1
Average dissimilarity = 48.13%

Species	Group Clay-30:1 Av. Abund	Group Ref-30:1 Av. Abund	Av. Diss	Diss/SD	Contrib. %	Cumul. %
Nucula nitidosa	2.72	0.68	2.73	1.67	5.68	5.68
Thyasira flexuosa	1.66	0.00	2.25	1.30	4.67	10.34
Pectinaria auricoma	1.32	0.00	1.81	1.23	3.76	14.10
Polyphysia crassa	1.32	0.00	1.80	1.22	3.73	17.83
Hyala vitrea	2.68	1.36	1.70	1.05	3.53	21.36
Cylichna cylindracea	0.75	1.56	1.69	1.07	3.52	24.88
Pectinaria belgica	1.36	0.00	1.63	1.33	3.39	28.28
Nephtys incisa	0.57	1.71	1.54	1.31	3.20	31.48
Goniada maculata	0.00	1.14	1.53	1.31	3.19	34.66
Edwardsiidae	1.14	0.00	1.53	1.30	3.17	37.83
Diplocirrus glaucus	1.56	1.14	1.53	1.34	3.17	41.01
Philine scabra	1.32	0.68	1.47	1.14	3.04	44.05
Thysanocardia procera	1.38	1.25	1.36	1.08	2.82	46.87
Abyssoninoe hibernica	2.28	1.38	1.34	1.19	2.79	49.66
Abra nitida	0.57	1.14	1.25	1.04	2.59	52.25
Terebellides stroemi	0.75	0.57	1.21	0.93	2.50	54.75
Eriopisa elongata	0.57	0.75	1.16	0.91	2.41	57.16
Echinocardium cordatum	0.68	0.57	1.11	0.90	2.31	59.47
Praxillella praetermissa	0.57	0.68	1.08	0.89	2.24	61.71
Brissopsis lyrifera	1.36	1.82	1.07	0.86	2.23	63.95
Spiophanes kroeyeri	1.82	1.36	1.00	0.85	2.09	66.03
Luidia sarsi	0.57	0.57	0.95	0.84	1.97	68.00
Chaetoderma nitidulum	1.14	1.14	0.94	0.84	1.96	69.96
Montacuta tenella	0.75	0.00	0.89	0.67	1.86	71.82
Echinocardium flavescens	0.57	0.00	0.85	0.66	1.76	73.58
Glycera alba	0.57	0.00	0.85	0.66	1.76	75.34
Trachythyone elongata	0.57	0.00	0.85	0.66	1.76	77.10
Mysella bidentata	0.68	0.00	0.81	0.67	1.68	78.78
Corbula gibba	3.03	2.56	0.78	1.49	1.63	80.41
Nemertea	0.00	0.57	0.75	0.66	1.57	81.98
Podarkeopsis helgolandicus	0.00	0.57	0.75	0.66	1.57	83.55
Glycera rouxii	0.57	0.00	0.69	0.67	1.44	84.99
Heteromastus filiformis	0.57	0.00	0.69	0.67	1.44	86.43
Laonice bahusiensis	0.57	0.00	0.69	0.67	1.44	87.87
Polydora spp	0.57	0.00	0.69	0.67	1.44	89.31
Scalibregma inflatum	0.57	0.00	0.69	0.67	1.44	90.74

Groups Lime-30:1 & Ref-30:1
Average dissimilarity = 68.87%

Species	Group Lime-30:1 Av. Abund	Group Ref-30:1 Av. Abund	Av. Diss	Diss/SD	Contrib. %	Cumul. %
Prionospio fallax	2.78	0.00	3.84	10.09	5.58	5.58
Glycera alba	2.21	0.00	3.10	5.70	4.49	10.07
Callianassa subterranea	0.00	1.82	2.60	3.66	3.78	13.85
Brissopsis lyrifera	0.00	1.82	2.60	3.82	3.77	17.62
Nephtys incisa	0.00	1.71	2.44	4.18	3.54	21.16
Tharyx killariensis	1.36	0.00	2.14	1.27	3.11	24.27
Thyasira flexuosa	1.68	0.00	2.06	1.27	2.98	27.26
Cylichna cylindracea	0.57	1.56	1.93	1.19	2.80	30.05
Praxillella praetermissa	1.82	0.68	1.88	1.50	2.73	32.78
Spiophanes kroeyeri	0.00	1.36	1.88	1.25	2.73	35.51
Heteromastus filiformis	1.56	0.00	1.85	1.26	2.69	38.20
Scalibregma inflatum	1.53	0.00	1.84	1.33	2.68	40.87
Nemertea	1.56	0.57	1.83	1.22	2.66	43.54
Abyssoninoe hibernica	0.57	1.38	1.81	1.04	2.63	46.16
Chaetoderma nitidulum	0.00	1.14	1.69	1.24	2.46	48.62
Pholoe baltica	1.14	0.00	1.69	1.22	2.46	51.07
Abra nitida	0.00	1.14	1.61	1.24	2.33	53.41
Chaetozone setosa	1.32	0.00	1.59	1.32	2.30	55.71

Amphiura chiajei	1.53	1.71	1.51	1.19	2.19	57.91
Hyala vitrea	1.43	1.36	1.42	0.88	2.07	59.97
Diplocirrus glaucus	1.32	1.14	1.36	0.99	1.98	61.95
Goniada maculata	0.57	1.14	1.27	1.03	1.85	63.80
Amphiura filiformis	2.98	3.68	1.20	0.86	1.74	65.54
Thysanocardia procera	1.14	1.25	1.18	0.95	1.71	67.25
Pilargis verrucosa	0.57	0.00	1.05	0.66	1.53	68.78
Podarkeopsis helgolandicus	0.57	0.57	1.04	0.80	1.50	70.28
Luidia sarsi	0.57	0.57	1.03	0.82	1.50	71.78
Philine scabra	0.00	0.68	0.99	0.64	1.44	73.22
Eriopisa elongata	0.00	0.75	0.98	0.65	1.43	74.65
Galathowenia oculata	0.85	0.00	0.95	0.67	1.38	76.03
Maldane sarsi	0.81	0.00	0.90	0.67	1.31	77.34
Polinices pulchella	0.68	0.00	0.89	0.67	1.29	78.63
Nucula nitidosa	0.00	0.68	0.89	0.65	1.29	79.92
Echinocardium cordatum	0.00	0.57	0.86	0.64	1.25	81.17
Terebellides stroemi	0.00	0.57	0.86	0.64	1.25	82.42
Trichobranchus roseus	0.75	0.00	0.84	0.67	1.22	83.64
Corbula gibba	2.71	2.56	0.83	1.36	1.20	84.83
Cerebratulus spp	0.57	0.00	0.75	0.67	1.09	85.92
Edwardsiidae	0.57	0.00	0.75	0.67	1.09	87.01
Magelona minuta	0.57	0.00	0.75	0.67	1.09	88.09
Pholoe pallida	0.57	0.00	0.75	0.67	1.09	89.18
Polinices montagui	0.57	0.00	0.75	0.67	1.09	90.26

Groups AC+clay-30:1 & AC+clay-30:14
Average dissimilarity = 86.10%

Species	Group AC+clay-30:1 Av. Abund	Group AC+clay-30:14 Av. Abund	Av. Diss	Diss/SD	Contrib. %	Cumul. %
Hyala vitrea	2.21	0.00	6.16	8.92	7.15	7.15
Montacuta ferruginosa	1.89	0.00	5.25	10.00	6.10	13.25
Trichobranchus roseus	1.82	0.00	5.20	4.10	6.04	19.29
Echinocardium cordatum	1.82	0.00	5.09	7.70	5.91	25.20
Cylichna cylindracea	2.10	0.34	5.05	1.93	5.86	31.06
Corbula gibba	1.89	0.48	4.63	2.64	5.37	36.44
Scalibregma inflatum	1.82	0.41	4.23	1.94	4.91	41.35
Montacuta tenella	1.43	0.00	3.84	1.26	4.46	45.81
Nephtys incisa	1.56	0.81	3.60	1.13	4.18	49.99
Luidia sarsi	1.25	0.00	3.28	1.33	3.80	53.79
Polycirrus spp	1.25	0.00	3.28	1.33	3.80	57.59
Abra nitida	1.25	0.34	3.14	1.15	3.64	61.24
Amphiura filiformis	1.14	0.34	3.10	1.16	3.60	64.84
Heteromastus filiformis	1.14	0.00	3.06	1.29	3.55	68.40
Polinices pulchella	1.14	0.00	3.06	1.29	3.55	71.95
Thyasira sarsii	0.00	1.03	2.89	1.13	3.36	75.31
Brissopsis lyrifera	1.14	0.34	2.82	1.14	3.28	78.59
Philine scabra	0.57	0.41	2.27	0.82	2.63	81.22
Abyssoninoe hibernica	0.57	0.51	2.04	0.80	2.37	83.59
Spiophanes kroeyeri	0.00	0.69	1.97	0.77	2.28	85.87
Callianassa subterranea	0.57	0.00	1.80	0.68	2.09	87.96
Cossura longocirrata	0.57	0.00	1.24	0.68	1.44	89.40
Diplocirrus glaucus	0.57	0.00	1.24	0.68	1.44	90.85

Groups Clay-30:1 & Clay-30:14
Average dissimilarity = 54.36%

Species	Group Clay-30:1 Av. Abund	Group Clay-30:14 Av. Abund	Av. Diss	Diss/SD	Contrib. %	Cumul. %
Scalibregma inflatum	0.57	3.76	3.84	2.35	7.06	7.06
Callianassa subterranea	1.71	0.00	2.02	7.07	3.72	10.77
Hyala vitrea	2.68	1.14	1.86	1.34	3.42	14.20
Nucula nitidosa	2.72	1.27	1.78	1.25	3.27	17.47
Nephtys incisa	0.57	2.02	1.75	1.59	3.22	20.69
Thyasira flexuosa	1.66	1.01	1.69	1.12	3.11	23.81

Amphiura chiajei	2.06	0.97	1.66	1.37	3.05	26.86
Pectinaria auricoma	1.32	0.00	1.64	1.25	3.01	29.87
Polyphysia crassa	1.32	0.00	1.63	1.25	2.99	32.87
Spiophanes kroeyeri	1.82	0.75	1.46	1.27	2.69	35.56
Pectinaria belgica	1.36	0.34	1.40	1.27	2.57	38.12
Chaetoderma nitidulum	1.14	0.41	1.33	1.26	2.45	40.57
Polydora spp	0.57	1.00	1.32	0.93	2.42	43.00
Glycera alba	0.57	1.48	1.30	1.24	2.40	45.40
Diplocirrus glaucus	1.56	1.54	1.28	1.09	2.35	47.75
Terebellides stroemi	0.75	0.83	1.24	0.93	2.28	50.03
Thysanocardia procera	1.38	1.03	1.21	1.12	2.23	52.26
Philine scabra	1.32	1.03	1.17	1.07	2.14	54.40
Edwardsiidae	1.14	1.17	1.13	1.04	2.07	56.47
Echinocardium cordatum	0.68	0.86	1.13	0.92	2.07	58.54
Abyssoninoe hibernica	2.28	1.37	1.03	1.04	1.90	60.44
Brissopsis lyrifera	1.36	1.71	1.00	1.05	1.84	62.28
Cylichna cylindracea	0.75	0.34	0.96	0.82	1.77	64.06
Polinices pulchella	0.57	0.69	0.93	0.89	1.70	65.76
Trachythyone elongata	0.57	0.34	0.86	0.77	1.58	67.34
Montacuta tenella	0.75	0.00	0.82	0.68	1.51	68.85
Heteromastus filiformis	0.57	0.34	0.80	0.78	1.48	70.33
Glycera rouxii	0.57	0.34	0.77	0.78	1.42	71.75
Eriopisa elongata	0.57	0.34	0.77	0.78	1.41	73.16
Echinocardium flavescens	0.57	0.00	0.76	0.68	1.40	74.56
Abra nitida	0.57	0.34	0.75	0.78	1.39	75.95
Mysella bidentata	0.68	0.00	0.74	0.68	1.36	77.31
Laonice bahusiensis	0.57	0.00	0.63	0.68	1.17	78.48
Luidia sarsi	0.57	0.00	0.63	0.68	1.17	79.64
Praxillella praetermissa	0.57	0.00	0.63	0.68	1.17	80.81
Antalis entalis	0.57	0.00	0.62	0.68	1.15	81.95
Cuspidaria obesa	0.57	0.00	0.62	0.68	1.15	83.10
Corbula gibba	3.03	2.59	0.59	1.20	1.08	84.18
Amphiura filiformis	3.96	3.59	0.59	1.01	1.08	85.26
Cerebratulus spp	0.00	0.45	0.54	0.48	0.99	86.25
Ampharete finmarchica	0.00	0.48	0.53	0.48	0.98	87.23
Prionospio fallax	0.00	0.45	0.50	0.48	0.91	88.14
Goniada maculata	0.00	0.41	0.49	0.48	0.89	89.03
Chaetopterus norvegicus	0.00	0.34	0.45	0.48	0.82	89.85
Chaetozone setosa	0.00	0.34	0.45	0.48	0.82	90.68

Groups AC+clay-30:14 & Clay-30:14
Average dissimilarity = 83.49%

Species	Group AC+clay-30:14 Av. Abund	Group Clay-30:14 Av. Abund	Av. Diss	Diss/SD	Contrib. %	Cumul. %
Scalibregma inflatum	0.41	3.76	7.41	2.36	8.87	8.87
Amphiura filiformis	0.34	3.59	6.95	3.90	8.33	17.20
Corbula gibba	0.48	2.59	4.70	2.12	5.62	22.82
Diplocirrus glaucus	0.00	1.54	3.19	1.81	3.82	26.65
Brissopsis lyrifera	0.34	1.71	3.08	1.82	3.69	30.34
Glycera alba	0.34	1.48	2.94	1.34	3.52	33.86
Abyssoninoe hibernica	0.51	1.37	2.92	1.73	3.50	37.36
Nephtys incisa	0.81	2.02	2.73	1.26	3.27	40.63
Nucula nitidosa	0.00	1.27	2.72	1.14	3.26	43.89
Thysanocardia procera	0.00	1.03	2.43	1.16	2.90	46.79
Hyala vitrea	0.00	1.14	2.31	1.14	2.77	49.56
Edwardsiidae	0.00	1.17	2.23	1.14	2.68	52.24
Polydora spp	0.00	1.00	2.19	0.71	2.63	54.87
Thyasira flexuosa	0.00	1.01	2.17	0.74	2.60	57.47
Philine scabra	0.41	1.03	2.15	1.12	2.57	60.05
Thyasira sarsii	1.03	0.34	2.08	1.06	2.50	62.54
Amphiura chiajei	0.00	0.97	1.98	0.79	2.38	64.92
Terebellides stroemi	0.00	0.83	1.91	0.74	2.29	67.21
Spiophanes kroeyeri	0.69	0.75	1.87	0.96	2.24	69.45
Echinocardium cordatum	0.00	0.86	1.63	0.79	1.96	71.41
Cerebratulus spp	0.34	0.45	1.46	0.69	1.75	73.16

Polinices pulchella	0.00	0.69	1.46	0.76	1.74	74.91
Goniada maculata	0.34	0.41	1.29	0.67	1.54	76.45
Chaetopterus norvegicus	0.34	0.34	1.24	0.65	1.49	77.94
Cylichna cylindracea	0.34	0.34	1.12	0.66	1.34	79.28
Golfingia vulgaris	0.34	0.34	1.12	0.66	1.34	80.63
Abra nitida	0.34	0.34	1.12	0.65	1.34	81.97
Ampharete finmarchica	0.00	0.48	0.92	0.49	1.10	83.07
Chaetozone setosa	0.00	0.34	0.89	0.49	1.07	84.14
Heteromastus filiformis	0.00	0.34	0.89	0.49	1.07	85.21
Phyllodoce groenlandica	0.00	0.34	0.88	0.49	1.05	86.27
Prionospio fallax	0.00	0.45	0.86	0.49	1.02	87.29
Ennucula tenuis	0.00	0.34	0.75	0.49	0.90	88.19
Ophiodromus flexuosus	0.00	0.34	0.75	0.49	0.90	89.09
Pectinaria belgica	0.00	0.34	0.75	0.49	0.90	89.99
Psamathe fusca	0.00	0.34	0.75	0.49	0.90	90.89

Groups Lime-30:1 & Lime-30:14
Average dissimilarity = 67.64%

Species	Group Lime-30:1<br>Av. Abund	Group Lime-30:14<br>Av. Abund	Av. Diss	Diss/SD	Contrib. %	Cumul. %
Prionospio fallax	2.78	0.00	3.23	5.21	4.77	4.77
Polydora spp	0.57	2.09	2.09	1.21	3.09	7.86
Thyasira flexuosa	1.68	1.99	2.05	1.25	3.02	10.89
Praxillella praetermissa	1.82	0.45	1.88	2.13	2.78	13.66
Philine scabra	0.00	1.59	1.86	1.50	2.75	16.42
Scalibregma inflatum	1.53	2.29	1.82	0.98	2.69	19.11
Tharyx killariensis	1.36	0.00	1.77	1.25	2.62	21.72
Galathowenia oculata	0.85	1.69	1.76	1.14	2.61	24.33
Nemertea	1.56	0.34	1.64	1.25	2.42	26.75
Diplocirrus glaucus	1.32	1.00	1.56	1.01	2.31	29.06
Heteromastus filiformis	1.56	0.34	1.53	1.21	2.26	31.32
Pectinaria auricoma	0.00	1.17	1.51	1.12	2.23	33.55
Thysanocardia procera	1.14	0.00	1.49	1.25	2.20	35.75
Hyala vitrea	1.43	1.39	1.46	1.04	2.16	37.91
Anobothrus gracilis	0.00	1.17	1.46	0.97	2.16	40.06
Pholoe baltica	1.14	0.88	1.36	1.16	2.01	42.07
Amphiura chiajei	1.53	1.71	1.34	0.98	1.97	44.05
Amphiura filiformis	2.98	3.95	1.31	1.07	1.94	45.99
Glycera alba	2.21	1.09	1.28	1.08	1.89	47.88
Chaetozone setosa	1.32	0.75	1.26	1.12	1.86	49.74
Trichobranchus roseus	0.75	1.02	1.24	0.93	1.84	51.57
Edwardsiidae	0.57	1.20	1.20	1.10	1.77	53.34
Diastylis boecki	0.00	1.03	1.14	1.11	1.69	55.03
Brissopsis lyrifera	0.00	0.85	1.02	0.70	1.50	56.53
Goniada maculata	0.57	0.41	0.99	0.79	1.47	58.00
Pectinaria koreni	0.00	0.81	0.98	0.76	1.45	59.45
Maldane sarsi	0.81	0.34	0.93	0.82	1.37	60.82
Ennucula tenuis	0.00	0.92	0.92	0.76	1.36	62.19
Labidoplax buskii	0.57	0.51	0.88	0.80	1.30	63.48
Pilargis verrucosa	0.57	0.00	0.85	0.66	1.26	64.74
Euchone papillosa	0.00	0.79	0.84	0.76	1.24	65.98
Cerebratulus spp	0.57	0.41	0.83	0.80	1.23	67.21
Antalis entalis	0.00	0.69	0.83	0.76	1.22	68.43
Lipobranchius jeffreysii	0.00	0.69	0.80	0.76	1.19	69.62
Cylichna cylindracea	0.57	0.34	0.77	0.77	1.14	70.76
Abyssoninoe hibernica	0.57	0.34	0.77	0.75	1.13	71.89
Polinices pulchella	0.68	0.00	0.76	0.67	1.12	73.01
Corbula gibba	2.71	2.46	0.74	1.40	1.10	74.11
Anthozoa	0.00	0.69	0.72	0.77	1.06	75.17
Brada villosa	0.00	0.69	0.71	0.77	1.05	76.22
Ophiodromus flexuosus	0.57	0.34	0.71	0.78	1.04	77.26
Owenia fusiformis	0.57	0.34	0.70	0.78	1.03	78.30
Parvicardium pinnulatum	0.00	0.69	0.69	0.77	1.01	79.31
Luidia sarsi	0.57	0.00	0.64	0.67	0.94	80.25
Magelona minuta	0.57	0.00	0.64	0.67	0.94	81.19

Pholoe pallida	0.57	0.00	0.64	0.67	0.94	82.13
Polinices montagui	0.57	0.00	0.64	0.67	0.94	83.07
Prionospio multibranchiata	0.57	0.00	0.64	0.67	0.94	84.01
Ampharete baltica	0.00	0.45	0.60	0.47	0.88	84.89
Callianassa subterranea	0.00	0.45	0.60	0.47	0.88	85.77
Mysella bidentata	0.00	0.51	0.56	0.48	0.82	86.60
Laonice bahusiensis	0.57	0.00	0.55	0.67	0.82	87.41
Pagurus bernhardus	0.57	0.00	0.55	0.67	0.82	88.23
Phascolion strombus	0.57	0.00	0.55	0.67	0.82	89.05
Podarkeopsis helgolandicus	0.57	0.00	0.55	0.67	0.82	89.87
Ophiuridae	0.00	0.41	0.54	0.47	0.80	90.66

Groups Clay-30:14 & Lime-30:14
Average dissimilarity = 61.80%

	Group Clay-30:14	Group Lime-30:14				
**Species**	**Av. Abund**	**Av. Abund**	**Av. Diss**	**Diss/SD**	**Contrib. %**	**Cumul. %**
Polydora spp	1.00	2.09	2.15	1.23	3.48	3.48
Thyasira flexuosa	1.01	1.99	2.08	1.22	3.37	6.85
Nephtys incisa	2.02	0.34	2.04	2.00	3.30	10.15
Scalibregma inflatum	3.76	2.29	1.79	1.26	2.89	13.04
Galathowenia oculata	0.00	1.69	1.73	1.11	2.81	15.84
Diplocirrus glaucus	1.54	1.00	1.61	1.33	2.60	18.45
Amphiura chiajei	0.97	1.71	1.57	1.25	2.53	20.98
Pectinaria auricoma	0.00	1.17	1.52	1.18	2.45	23.43
Brissopsis lyrifera	1.71	0.85	1.48	1.61	2.40	25.83
Anobothrus gracilis	0.00	1.17	1.46	1.02	2.37	28.20
Hyala vitrea	1.14	1.39	1.44	1.15	2.33	30.53
Nucula nitidosa	1.27	0.41	1.42	1.06	2.30	32.83
Abyssoninoe hibernica	1.37	0.34	1.37	1.33	2.21	35.04
Thysanocardia procera	1.03	0.00	1.29	1.14	2.09	37.13
Edwardsiidae	1.17	1.20	1.27	1.09	2.06	39.19
Philine scabra	1.03	1.59	1.21	1.01	1.96	41.15
Pholoe baltica	0.00	0.88	1.20	0.71	1.95	43.10
Diastylis boecki	0.00	1.03	1.15	1.16	1.86	44.96
Trichobranchus roseus	0.34	1.02	1.14	0.92	1.84	46.80
Terebellides stroemi	0.83	0.34	1.12	0.88	1.81	48.61
Glycera alba	1.48	1.09	1.10	1.00	1.78	50.39
Pectinaria koreni	0.41	0.81	1.07	0.85	1.73	52.12
Ennucula tenuis	0.34	0.92	1.06	0.91	1.72	53.84
Echinocardium cordatum	0.86	0.34	1.04	0.88	1.68	55.52
Spiophanes kroeyeri	0.75	0.41	0.95	0.86	1.54	57.05
Chaetozone setosa	0.34	0.75	0.93	0.86	1.50	58.56
Euchone papillosa	0.00	0.79	0.84	0.78	1.37	59.92
Antalis entalis	0.00	0.69	0.83	0.79	1.34	61.27
Lipobranchius jeffreysii	0.00	0.69	0.81	0.78	1.31	62.57
Polinices pulchella	0.69	0.00	0.80	0.77	1.30	63.88
Cerebratulus spp	0.45	0.41	0.79	0.66	1.29	65.16
Goniada maculata	0.41	0.41	0.76	0.66	1.23	66.39
Chaetopterus norvegicus	0.34	0.34	0.74	0.66	1.20	67.59
Anthozoa	0.00	0.69	0.73	0.79	1.17	68.77
Brada villosa	0.00	0.69	0.71	0.79	1.16	69.92
Amphiura filiformis	3.59	3.95	0.70	1.26	1.13	71.05
Parvicardium pinnulatum	0.00	0.69	0.69	0.80	1.12	72.17
Eriopisa elongata	0.34	0.34	0.67	0.66	1.08	73.25
Heteromastus filiformis	0.34	0.34	0.65	0.65	1.05	74.30
Chaetoderma nitidulum	0.41	0.34	0.64	0.68	1.04	75.34
Ophiodromus flexuosus	0.34	0.34	0.62	0.66	1.01	76.35
Cylichna cylindracea	0.34	0.34	0.62	0.66	1.00	77.34
Ampharete baltica	0.00	0.45	0.60	0.49	0.97	78.32
Callianassa subterranea	0.00	0.45	0.60	0.49	0.97	79.29
Corbula gibba	2.59	2.46	0.58	1.43	0.93	80.22
Mysella bidentata	0.00	0.51	0.56	0.49	0.91	81.13
Ophiuridae	0.00	0.41	0.54	0.49	0.88	82.01
Ampharete finmarchica	0.48	0.00	0.54	0.48	0.87	82.88
Labidoplax buskii	0.00	0.51	0.52	0.49	0.85	83.73

Prionospio fallax	0.45	0.00	0.50	0.48	0.81	84.54
Eclysippe eliasoni	0.00	0.48	0.50	0.49	0.80	85.34
Nemertea	0.00	0.34	0.46	0.49	0.74	86.08
Phyllodoce groenlandica	0.34	0.00	0.45	0.48	0.73	86.81
Praxillella praetermissa	0.00	0.45	0.45	0.49	0.72	87.54
Pectinaria belgica	0.34	0.00	0.41	0.48	0.67	88.21
Psamathe fusca	0.34	0.00	0.41	0.48	0.67	88.88
Thyasira sarsii	0.34	0.00	0.41	0.48	0.67	89.55
Gattyana cirrhosa	0.41	0.00	0.41	0.48	0.67	90.22

Groups Ref-30:1 & Ref-30:14
Average dissimilarity = 60.49%

Species	Group Ref-30:1 Av. Abund	Group Ref-30:14 Av. Abund	Av. Diss	Diss/SD	Contrib. %	Cumul. %
Scalibregma inflatum	0.00	3.71	5.37	5.67	8.87	8.87
Corbula gibba	2.56	0.34	3.23	2.71	5.34	14.21
Ophiodromus flexuosus	0.00	1.91	2.76	12.35	4.57	18.78
Amphiura chiajei	1.71	0.00	2.49	12.54	4.11	22.90
Streblosoma bairdi	0.00	1.44	2.07	1.89	3.42	26.31
Cylichna cylindracea	1.56	0.41	2.03	1.26	3.35	29.66
Terebellides stroemi	0.57	1.45	1.91	1.20	3.16	32.82
Chaetoderma nitidulum	1.14	0.00	1.72	1.36	2.85	35.67
Brissopsis lyrifera	1.82	0.69	1.67	1.27	2.76	38.43
Abra nitida	1.14	0.00	1.64	1.35	2.71	41.14
Abyssoninoe hibernica	1.38	1.31	1.64	1.14	2.71	43.85
Hyala vitrea	1.36	0.69	1.62	1.21	2.68	46.53
Diplocirrus glaucus	1.14	2.22	1.58	1.22	2.61	49.14
Glycera alba	0.00	1.09	1.57	1.16	2.60	51.74
Goniada maculata	1.14	0.34	1.53	1.18	2.52	54.26
Pholoe baltica	0.00	1.03	1.51	1.18	2.50	56.77
Praxillella praetermissa	0.68	0.86	1.43	0.92	2.37	59.13
Thysanocardia procera	1.25	1.09	1.34	1.02	2.22	61.35
Nemertea	0.57	1.03	1.33	1.03	2.19	63.54
Nucula nitidosa	0.68	0.69	1.32	0.98	2.17	65.72
Philine scabra	0.68	0.69	1.31	0.96	2.16	67.88
Echinocardium cordatum	0.57	0.75	1.26	0.94	2.09	69.97
Pectinaria belgica	0.00	0.83	1.26	0.77	2.08	72.05
Amphiura filiformis	3.68	2.84	1.24	2.02	2.05	74.10
Eriopisa elongata	0.75	0.34	1.18	0.83	1.95	76.05
Spiophanes kroeyeri	1.36	2.08	1.11	0.72	1.83	77.88
Heteromastus filiformis	0.00	0.75	1.09	0.77	1.81	79.69
Pectinaria auricoma	0.00	0.75	1.09	0.77	1.81	81.49
Edwardsiidae	0.00	0.69	1.01	0.78	1.67	83.16
Parvicardium pinnulatum	0.00	0.69	0.98	0.79	1.63	84.79
Callianassa subterranea	1.82	1.70	0.96	0.98	1.59	86.38
Podarkeopsis helgolandicus	0.57	0.00	0.85	0.68	1.40	87.78
Lipobranchius jeffreysii	0.57	0.00	0.76	0.68	1.26	89.04
Luidia sarsi	0.57	0.00	0.76	0.68	1.26	90.30

Groups AC+clay-30:14 & Ref-30:14
Average dissimilarity = 86.26%

Species	Group AC+clay-30:14 Av. Abund	Group Ref-30:14 Av. Abund	Av. Diss	Diss/SD	Contrib. %	Cumul. %
Scalibregma inflatum	0.41	3.71	7.25	3.22	8.41	8.41
Amphiura filiformis	0.34	2.84	5.49	3.30	6.36	14.77
Diplocirrus glaucus	0.00	2.22	4.92	8.37	5.71	20.47
Ophiodromus flexuosus	0.00	1.91	4.22	11.76	4.90	25.37
Callianassa subterranea	0.00	1.70	3.75	1.81	4.35	29.72
Streblosoma bairdi	0.00	1.44	3.15	1.90	3.65	33.36
Spiophanes kroeyeri	0.69	2.08	3.09	1.60	3.58	36.94
Terebellides stroemi	0.00	1.45	3.08	1.12	3.57	40.51
Abyssoninoe hibernica	0.51	1.31	2.86	1.17	3.31	43.82
Nephtys incisa	0.81	1.78	2.59	1.46	3.01	46.83

Thysanocardia procera	0.00	1.09	2.48	1.18	2.87	49.71
Pholoe baltica	0.00	1.03	2.33	1.19	2.71	52.41
Nemertea	0.00	1.03	2.29	1.18	2.66	55.07
Glycera alba	0.34	1.09	2.27	1.11	2.64	57.71
Thyasira sarsii	1.03	0.00	2.27	1.18	2.63	60.34
Pectinaria belgica	0.00	0.83	1.98	0.78	2.29	62.63
Praxillella praetermissa	0.00	0.86	1.91	0.79	2.22	64.85
Philine scabra	0.41	0.69	1.76	0.89	2.04	66.89
Heteromastus filiformis	0.00	0.75	1.68	0.77	1.95	68.83
Pectinaria auricoma	0.00	0.75	1.68	0.77	1.95	70.78
Echinocardium cordatum	0.00	0.75	1.66	0.80	1.92	72.70
Nucula nitidosa	0.00	0.69	1.64	0.80	1.91	74.61
Brissopsis lyrifera	0.34	0.69	1.64	0.86	1.90	76.51
Edwardsiidae	0.00	0.69	1.56	0.79	1.81	78.32
Hyala vitrea	0.00	0.69	1.52	0.80	1.76	80.08
Parvicardium pinnulatum	0.00	0.69	1.49	0.80	1.73	81.81
Corbula gibba	0.48	0.34	1.39	0.69	1.61	83.42
Cylichna cylindracea	0.34	0.41	1.32	0.69	1.53	84.94
Goniada maculata	0.34	0.34	1.19	0.67	1.38	86.33
Chaetopterus norvegicus	0.34	0.34	1.11	0.67	1.28	87.61
Praxillella affinis	0.00	0.41	0.92	0.49	1.06	88.67
Thyasira flexuosa	0.00	0.34	0.84	0.49	0.98	89.65
Abra nitida	0.34	0.00	0.81	0.49	0.93	90.58

Groups Clay-30:14 & Ref-30:14
Average dissimilarity = 55.55%

Species	Group Clay-30:14 Av. Abund	Group Ref-30:14 Av. Abund	Av. Diss	Diss/SD	Contrib. %	Cumul. %
Corbula gibba	2.59	0.34	2.94	2.87	5.29	5.29
Callianassa subterranea	0.00	1.70	2.21	1.82	3.98	9.27
Ophiodromus flexuosus	0.34	1.91	2.04	2.11	3.67	12.94
Streblosoma bairdi	0.00	1.44	1.86	1.88	3.36	16.30
Spiophanes kroeyeri	0.75	2.08	1.86	1.35	3.34	19.64
Terebellides stroemi	0.83	1.45	1.75	1.17	3.15	22.79
Nucula nitidosa	1.27	0.69	1.47	1.21	2.64	25.43
Polydora spp	1.00	0.34	1.41	0.87	2.54	27.97
Thyasira flexuosa	1.01	0.34	1.41	0.90	2.54	30.52
Pholoe baltica	0.00	1.03	1.36	1.18	2.46	32.97
Abyssoninoe hibernica	1.37	1.31	1.36	1.33	2.45	35.42
Brissopsis lyrifera	1.71	0.69	1.36	1.18	2.44	37.86
Nemertea	0.00	1.03	1.35	1.18	2.43	40.29
Edwardsiidae	1.17	0.69	1.32	1.09	2.37	42.67
Echinocardium cordatum	0.86	0.75	1.29	1.01	2.32	44.99
Hyala vitrea	1.14	0.69	1.29	1.08	2.32	47.31
Amphiura chiajei	0.97	0.00	1.23	0.80	2.21	49.52
Pectinaria belgica	0.34	0.83	1.21	0.88	2.17	51.69
Glycera alba	1.48	1.09	1.19	1.01	2.15	53.84
Philine scabra	1.03	0.69	1.16	1.01	2.09	55.93
Thysanocardia procera	1.03	1.09	1.15	1.01	2.06	57.99
Praxillella praetermissa	0.00	0.86	1.13	0.79	2.03	60.02
Heteromastus filiformis	0.34	0.75	1.09	0.88	1.96	61.97
Scalibregma inflatum	3.76	3.71	1.04	1.36	1.88	63.85
Amphiura filiformis	3.59	2.84	1.02	1.68	1.83	65.68
Diplocirrus glaucus	1.54	2.22	1.01	0.85	1.81	67.49
Pectinaria auricoma	0.00	0.75	0.98	0.78	1.77	69.27
Parvicardium pinnulatum	0.00	0.69	0.89	0.79	1.60	70.87
Polinices pulchella	0.69	0.00	0.88	0.79	1.58	72.45
Goniada maculata	0.41	0.34	0.82	0.69	1.47	73.92
Cylichna cylindracea	0.34	0.41	0.77	0.68	1.39	75.30
Chaetozone setosa	0.34	0.34	0.76	0.67	1.36	76.67
Chaetopterus norvegicus	0.34	0.34	0.73	0.66	1.32	77.99
Eriopisa elongata	0.34	0.34	0.68	0.67	1.22	79.21
Cerebratulus spp	0.45	0.00	0.60	0.49	1.08	80.28
Ampharete finmarchica	0.48	0.00	0.59	0.49	1.06	81.34
Prionospio fallax	0.45	0.00	0.55	0.49	0.98	82.32

Praxillella affinis	0.00	0.41	0.54	0.49	0.97	83.29
Phyllodoce groenlandica	0.34	0.00	0.50	0.49	0.90	84.19
Mysia undata	0.00	0.34	0.46	0.49	0.83	85.02
Ennucula tenuis	0.34	0.00	0.45	0.49	0.82	85.84
Psamathe fusca	0.34	0.00	0.45	0.49	0.82	86.66
Thyasira sarsii	0.34	0.00	0.45	0.49	0.82	87.47
Chaetoderma nitidulum	0.41	0.00	0.45	0.49	0.81	88.28
Gattyana cirrhosa	0.41	0.00	0.45	0.49	0.81	89.09
Pectinaria koreni	0.41	0.00	0.45	0.49	0.81	89.90
Phaxas pellucida	0.41	0.00	0.45	0.49	0.81	90.70

Groups Lime-30:14 & Ref-30:14
Average dissimilarity = 68.25%

	Group Lime-30:14	Group Ref-30:14				
**Species**	**Av. Abund**	**Av. Abund**	**Av. Diss**	**Diss/SD**	**Contrib. %**	**Cumul. %**
Corbula gibba	2.46	0.34	2.55	2.41	3.74	3.74
Polydora spp	2.09	0.34	2.17	1.26	3.17	6.92
Spiophanes kroeyeri	0.41	2.08	2.08	1.83	3.05	9.96
Thyasira flexuosa	1.99	0.34	2.06	1.22	3.02	12.98
Ophiodromus flexuosus	0.34	1.91	1.96	1.99	2.87	15.85
Amphiura chiajei	1.71	0.00	1.92	1.87	2.81	18.66
Diplocirrus glaucus	1.00	2.22	1.81	1.71	2.66	21.32
Nephtys incisa	0.34	1.78	1.79	1.82	2.62	23.94
Galathowenia oculata	1.69	0.00	1.76	1.12	2.58	26.52
Callianassa subterranea	0.45	1.70	1.75	1.37	2.57	29.09
Streblosoma bairdi	0.00	1.44	1.73	1.80	2.54	31.63
Scalibregma inflatum	2.29	3.71	1.69	1.26	2.48	34.11
Terebellides stroemi	0.34	1.45	1.64	1.19	2.40	36.51
Hyala vitrea	1.39	0.69	1.50	1.17	2.20	38.71
Abyssoninoe hibernica	0.34	1.31	1.49	1.18	2.19	40.89
Anobothrus gracilis	1.17	0.00	1.49	1.03	2.18	43.08
Philine scabra	1.59	0.69	1.46	1.17	2.14	45.21
Pholoe baltica	0.88	1.03	1.37	1.11	2.01	47.23
Thysanocardia procera	0.00	1.09	1.35	1.14	1.97	49.20
Amphiura filiformis	3.95	2.84	1.33	1.79	1.94	51.14
Pectinaria auricoma	1.17	0.75	1.32	1.14	1.94	53.08
Edwardsiidae	1.20	0.69	1.23	1.13	1.80	54.88
Brissopsis lyrifera	0.85	0.69	1.22	0.97	1.79	56.67
Diastylis boecki	1.03	0.00	1.17	1.17	1.71	58.38
Nemertea	0.34	1.03	1.16	1.06	1.70	60.08
Praxillella praetermissa	0.45	0.86	1.15	0.85	1.69	61.77
Glycera alba	1.09	1.09	1.15	0.99	1.68	63.45
Pectinaria belgica	0.00	0.83	1.05	0.76	1.54	64.99
Trichobranchus roseus	1.02	0.00	1.05	0.80	1.53	66.52
Pectinaria koreni	0.81	0.00	1.01	0.79	1.47	68.00
Nucula nitidosa	0.41	0.69	1.00	0.89	1.46	69.46
Heteromastus filiformis	0.34	0.75	0.99	0.84	1.44	70.90
Echinocardium cordatum	0.34	0.75	0.98	0.85	1.44	72.34
Parvicardium pinnulatum	0.69	0.69	0.97	0.92	1.42	73.76
Ennucula tenuis	0.92	0.00	0.94	0.79	1.38	75.14
Chaetozone setosa	0.75	0.34	0.93	0.87	1.36	76.51
Euchone papillosa	0.79	0.00	0.86	0.78	1.26	77.76
Antalis entalis	0.69	0.00	0.85	0.79	1.24	79.00
Lipobranchius jeffreysii	0.69	0.00	0.82	0.79	1.20	80.20
Anthozoa	0.69	0.00	0.74	0.80	1.08	81.28
Chaetopterus norvegicus	0.34	0.34	0.73	0.65	1.07	82.35
Goniada maculata	0.41	0.34	0.73	0.68	1.06	83.42
Brada villosa	0.69	0.00	0.73	0.80	1.06	84.48
Cylichna cylindracea	0.34	0.41	0.71	0.67	1.05	85.53
Eriopisa elongata	0.34	0.34	0.68	0.66	1.00	86.53
Ampharete baltica	0.45	0.00	0.61	0.49	0.90	87.42
Mysella bidentata	0.51	0.00	0.57	0.49	0.83	88.26
Ophiuridae	0.41	0.00	0.55	0.49	0.81	89.07
Labidoplax buskii	0.51	0.00	0.53	0.49	0.78	89.85
Eclysippe eliasoni	0.48	0.00	0.50	0.49	0.74	90.58

Table S4. SIMPER-analysis from 80-95 m.

SIMPER Similarity Percentages - species contributions

One-Way Analysis

Data type: Abundance
Variable selection: All

Parameters
Transform: Fourth root
Resemblance: S17 Bray Curtis similarity
Cut off for low contributions: 90%

Factor Groups

**Sample**	**sample unit**
AC+clay-95:1A	AC+clay-95:1
AC+clay-95:1B	AC+clay-95:1
AC+clay-95:1C	AC+clay-95:1
Ref-80:1A	Ref-80:1
Ref-80:1B	Ref-80:1
Ref-80:1C	Ref-80:1
AC+clay-95:14A	AC+clay-95:14
AC+clay-95:14B	AC+clay-95:14
AC+clay-95:14C	AC+clay-95:14
AC+clay-95:14D	AC+clay-95:14
AC+clay-95:14E	AC+clay-95:14
Ref-80:14A	Ref-80:14
Ref-80:14B	Ref-80:14
Ref-80:14C	Ref-80:14
Ref-80:14D	Ref-80:14
Ref-80:14E	Ref-80:14
Ref-95:14A	Ref-95:14
Ref-95:14B	Ref-95:14
Ref-95:14C	Ref-95:14
Ref-95:14D	Ref-95:14
Ref-95:14E	Ref-95:14

Group AC+clay-95:1
Average similarity: 51.39%

**Species**	**Av. Abund**	**Av. Sim**	**Sim/SD**	**Contrib. %**	**Cumul. %**
Thyasira equalis	3.72	7.77	11.11	15.12	15.12
Spiophanes kroeyeri	2.87	6.03	14.33	11.73	26.86
Yoldiella philippiana	2.93	5.86	33.17	11.41	38.27
Phascolion strombus	2.32	4.35	3.40	8.46	46.73
Paramphinome jeffreysii	2.21	4.11	4.90	8.00	54.73
Aphelochaeta marioni	2.06	3.95	6.45	7.69	62.42
Ceratocephale loveni	2.10	3.95	6.45	7.69	70.11
Abra nitida	1.89	3.71	14.33	7.21	77.32
Chaetozone setosa	1.69	1.54	0.58	2.99	80.31
Heteromastus filiformis	1.36	1.35	0.58	2.63	82.94
Philomedes brenda	1.49	1.35	0.58	2.63	85.57
Pista cristata	1.25	1.29	0.58	2.52	88.09
Prionospio dubia	1.14	1.29	0.58	2.52	90.61

Group Ref-80:1
Average similarity: 52.02%

**Species**	**Av. Abund**	**Av. Sim**	**Sim/SD**	**Contrib. %**	**Cumul. %**
Thyasira equalis	3.87	5.13	18.92	9.87	9.87
Spiophanes kroeyeri	3.38	4.37	6.80	8.41	18.28
Yoldiella philippiana	2.95	3.69	6.59	7.09	25.37
Streblosoma bairdi	2.71	3.61	27.64	6.94	32.31
Aphelochaeta marioni	2.29	3.02	6.42	5.80	38.11
Abyssoninoe hibernica	2.24	2.93	28.23	5.63	43.74
Ennucula tenuis	2.25	2.62	8.13	5.03	48.77

Heteromastus filiformis	2.13	2.62	8.13	5.03	53.81
Amphiura filiformis	1.93	2.54	6.42	4.88	58.69
Abra nitida	2.33	2.52	15.17	4.85	63.54
Diplocirrus glaucus	2.00	2.52	15.17	4.85	68.39
Ampelisca gibba	1.82	2.38	18.72	4.58	72.97
Paramphitrite tetrabranchia	1.71	2.38	18.72	4.58	77.54
Pista cristata	1.50	1.10	0.58	2.12	79.67
Paramphinome jeffreysii	1.50	1.04	0.58	1.99	81.66
Brissopsis lyrifera	1.38	0.84	0.58	1.61	83.27
Chaetozone setosa	1.14	0.84	0.58	1.61	84.88
Eclysippe eliasoni	1.25	0.84	0.58	1.61	86.49
Glycera alba	1.38	0.84	0.58	1.61	88.11
Sosane sulcata	1.14	0.84	0.58	1.61	89.72
Euchone papillosa	1.14	0.79	0.58	1.51	91.23

Group AC+clay-95:14
Average similarity: 63.09%

Species	Av. Abund	Av. Sim	Sim/SD	Contrib. %	Cumul. %
Abra nitida	2.95	5.86	3.98	9.29	9.29
Spiophanes kroeyeri	2.85	5.40	6.81	8.57	17.85
Thyasira equalis	2.91	5.05	3.86	8.01	25.86
Aphelochaeta marioni	2.41	5.01	12.58	7.94	33.80
Paramphinome jeffreysii	3.22	4.53	1.13	7.18	40.98
Heteromastus filiformis	2.25	4.50	8.56	7.14	48.11
Chaetozone setosa	2.23	4.38	7.52	6.94	55.05
Ceratocephale loveni	2.14	4.26	12.47	6.75	61.80
Glycera alba	2.09	4.17	14.06	6.61	68.41
Philine scabra	2.07	4.05	9.51	6.42	74.83
Arrhis phyllonyx	1.78	2.64	1.13	4.19	79.02
Eudorella emarginata	1.65	2.56	1.14	4.06	83.08
Leucon nasica	1.56	2.51	1.15	3.98	87.06
Yoldiella philippiana	1.72	2.50	1.14	3.96	91.02

Group Ref-80:14
Average similarity: 65.88%

Species	Av. Abund	Av. Sim	Sim/SD	Contrib. %	Cumul. %
Paramphinome jeffreysii	3.75	4.81	8.17	7.30	7.30
Thyasira equalis	3.27	4.29	17.52	6.51	13.81
Spiophanes kroeyeri	3.17	3.93	6.87	5.96	19.77
Heteromastus filiformis	2.86	3.82	14.78	5.80	25.57
Aphelochaeta marioni	3.02	3.73	4.71	5.66	31.23
Chaetozone setosa	2.81	3.41	4.33	5.18	36.41
Streblosoma bairdi	2.63	3.36	31.70	5.10	41.51
Lipobranchius jeffreysii	2.42	3.15	16.76	4.78	46.29
Glycera alba	2.24	3.03	13.19	4.60	50.89
Abyssoninoe hibernica	2.31	2.98	12.02	4.53	55.42
Scalibregma inflatum	2.26	2.97	10.92	4.50	59.92
Ceratocephale loveni	2.23	2.96	15.15	4.50	64.42
Yoldiella philippiana	2.29	2.75	4.95	4.17	68.59
Diplocirrus glaucus	1.99	2.47	8.45	3.76	72.34
Eclysippe eliasoni	1.89	2.44	12.22	3.71	76.05
Amphiura chiajei	1.75	1.66	1.15	2.51	78.57
Rhodine loveni	1.69	1.59	1.14	2.41	80.98
Brada villosa	1.69	1.58	1.14	2.40	83.38
Abra nitida	1.62	1.53	1.13	2.32	85.70
Cerebratulus spp	1.56	1.52	1.15	2.31	88.01
Goniada maculata	1.44	1.47	1.16	2.23	90.24

Group Ref-95:14
Average similarity: 64.70%

Species	Av. Abund	Av. Sim	Sim/SD	Contrib. %	Cumul. %
Chaetozone setosa	4.02	6.48	25.74	10.01	10.01
Aphelochaeta marioni	3.27	5.24	31.79	8.10	18.11
Spiophanes kroeyeri	3.26	4.96	9.09	7.67	25.79
Paramphinome jeffreysii	3.08	4.37	4.58	6.76	32.54
Heteromastus filiformis	2.81	4.22	5.30	6.52	39.06
Thyasira equalis	3.00	4.07	3.37	6.29	45.35
Ceratocephale loveni	2.57	3.74	9.54	5.79	51.14
Leucon nasica	2.33	3.72	10.51	5.75	56.88
Abra nitida	2.41	3.71	12.58	5.74	62.62
Scalibregma inflatum	2.43	3.68	5.38	5.69	68.31
Arrhis phyllonyx	2.21	3.23	4.94	5.00	73.31
Goniada maculata	1.91	3.07	7.37	4.75	78.05
Prionospio cirrifera	1.86	2.11	1.15	3.25	81.31
Eudorella emarginata	1.80	2.04	1.16	3.15	84.45
Melinna cristata	1.37	1.68	1.16	2.60	87.05
Glycera alba	1.31	1.03	0.62	1.59	88.64
Yoldiella philippiana	1.23	0.96	0.62	1.49	90.13

Groups AC+clay-95:1 & Ref-80:1
Average dissimilarity = 57.35%

Species	Group AC+clay-95:1 Av. Abund	Group Ref-80:1 Av. Abund	Av. Diss	Diss/SD	Contrib. %	Cumul. %
Streblosoma bairdi	0.00	2.71	2.30	12.30	4.00	4.00
Ampelisca gibba	0.00	1.82	1.54	15.01	2.68	6.69
Phascolion strombus	2.32	0.57	1.46	1.82	2.55	9.23
Paramphitrite tetrabranchia	0.00	1.71	1.45	13.23	2.53	11.77
Ennucula tenuis	0.57	2.25	1.43	1.70	2.50	14.26
Abyssoninoe hibernica	0.57	2.24	1.37	2.01	2.40	16.66
Ceratocephale loveni	2.10	0.75	1.27	1.61	2.21	18.87
Philomedes brenda	1.49	0.00	1.22	1.32	2.12	20.99
Brissopsis lyrifera	0.00	1.38	1.20	1.29	2.10	23.09
Amphiura filiformis	0.57	1.93	1.18	1.52	2.05	25.14
Chaetozone setosa	1.69	1.14	1.12	1.39	1.95	27.09
Eclysippe eliasoni	0.00	1.25	1.09	1.32	1.91	29.00
Amphiura chiajei	0.00	1.32	1.09	1.27	1.91	30.91
Scalibregma inflatum	1.25	0.00	1.08	1.29	1.88	32.79
Tanaidacea	1.25	0.00	1.02	1.31	1.79	34.58
Glycera alba	0.57	1.38	1.02	1.17	1.77	36.35
Sosane sulcata	0.00	1.14	1.00	1.33	1.75	38.10
Luidia sarsi	0.00	1.14	0.94	1.33	1.64	39.73
Notomastus latericeus	0.00	1.14	0.94	1.33	1.64	41.37
Polynoidae	0.00	1.14	0.94	1.33	1.64	43.01
Prionospio dubia	1.14	0.68	0.91	1.22	1.59	44.60
Pista cristata	1.25	1.50	0.91	1.15	1.58	46.18
Euchone papillosa	0.68	1.14	0.90	1.21	1.57	47.75
Melinna cristata	0.68	0.75	0.83	0.87	1.44	49.19
Paramphinome jeffreysii	2.21	1.50	0.82	0.94	1.43	50.62
Heteromastus filiformis	1.36	2.13	0.81	0.96	1.41	52.03
Praxillella affinis	0.57	1.14	0.80	1.05	1.40	53.43
Hyala vitrea	0.75	0.57	0.78	0.91	1.36	54.78
Rhodine loveni	1.25	1.14	0.75	0.97	1.30	56.08
Gnathia spp	0.57	0.68	0.73	0.91	1.28	57.37
Diastyloides serratus	0.89	0.00	0.73	0.67	1.27	58.64
Diplocirrus glaucus	1.14	2.00	0.71	0.98	1.23	59.87
Neoamphitrite affinis	0.57	0.57	0.65	0.84	1.14	61.00
Campylaspis costata	0.57	0.57	0.63	0.84	1.09	62.10
Phyllodoce rosea	0.57	0.57	0.63	0.84	1.09	63.19
Terebellides stroemi	0.57	0.57	0.63	0.84	1.09	64.28
Maldane sarsi	0.00	0.75	0.60	0.67	1.04	65.32
Glycera rouxii	0.00	0.68	0.58	0.67	1.01	66.33
Montacuta tenella	0.00	0.68	0.58	0.67	1.01	67.34
Echinocardium flavescens	0.00	0.68	0.54	0.67	0.94	68.28

Lipobranchius jeffreysii	0.00	0.68	0.54	0.67	0.94	69.22
Philine aperta	0.00	0.68	0.54	0.67	0.94	70.16
Diastylis boecki	0.57	0.00	0.52	0.67	0.90	71.06
Chaetoderma nitidulum	0.00	0.57	0.51	0.67	0.90	71.95
Galathowenia oculata	0.00	0.57	0.51	0.67	0.90	72.85
Glycinde nordmanni	0.00	0.57	0.51	0.67	0.90	73.75
Harpinia antennaria	0.00	0.57	0.51	0.67	0.90	74.64
Leucothoe lilljeborgii	0.00	0.57	0.51	0.67	0.90	75.54
Spatangidae	0.00	0.57	0.51	0.67	0.90	76.44
Westwoodilla caecula	0.00	0.57	0.51	0.67	0.90	77.33
Abra nitida	1.89	2.33	0.49	1.02	0.86	78.19
Harpinia crenulata	0.00	0.57	0.49	0.67	0.85	79.04
Iphitime hartmanae	0.00	0.57	0.49	0.67	0.85	79.89
Nereiphylla lutea	0.00	0.57	0.49	0.67	0.85	80.74
Philine scabra	0.00	0.57	0.49	0.67	0.85	81.59
Polycirrus spp	0.00	0.57	0.49	0.67	0.85	82.44
Proclea graffii	0.00	0.57	0.49	0.67	0.85	83.29
Pseudamussium peslutrae	0.00	0.57	0.49	0.67	0.85	84.14
Chaetoparia nilssoni	0.57	0.00	0.47	0.67	0.82	84.96
Corbula gibba	0.57	0.00	0.47	0.67	0.82	85.78
Mysella bidentata	0.57	0.00	0.47	0.67	0.82	86.60
Neoamphitrite grayi	0.57	0.00	0.47	0.67	0.82	87.42
Leptostylis longimana	0.57	0.00	0.47	0.67	0.81	88.23
Leucon nasica	0.57	0.00	0.47	0.67	0.81	89.04
Ophiocten affinis	0.57	0.00	0.47	0.67	0.81	89.85
Phylo norvegica	0.57	0.00	0.47	0.67	0.81	90.67

Groups AC+clay-95:1 & AC+clay-95:14
Average dissimilarity = 54.26%

Species	Group AC+clay-95:1 Av. Abund	Group AC+clay-95:14 Av. Abund	Av. Diss	Diss/SD	Contrib. %	Cumul. %
Phascolion strombus	2.32	0.00	2.53	4.35	4.67	4.67
Philine scabra	0.00	2.07	2.24	7.61	4.13	8.79
Paramphinome jeffreysii	2.21	3.22	2.03	2.42	3.74	12.53
Arrhis phyllonyx	0.00	1.78	1.96	1.77	3.62	16.15
Eudorella emarginata	0.00	1.65	1.82	1.85	3.36	19.50
Glycera alba	0.57	2.09	1.59	1.81	2.94	22.44
Philomedes brenda	1.49	0.34	1.45	1.30	2.66	25.10
Leucon nasica	0.57	1.56	1.36	1.31	2.50	27.61
Yoldiella philippiana	2.93	1.72	1.31	1.24	2.42	30.03
Tanaidacea	1.25	0.00	1.30	1.34	2.39	32.42
Prionospio dubia	1.14	0.00	1.27	1.35	2.34	34.77
Scalibregma inflatum	1.25	0.34	1.27	1.19	2.33	37.10
Pista cristata	1.25	0.34	1.26	1.20	2.32	39.42
Abra nitida	1.89	2.95	1.21	2.05	2.22	41.65
Polynoidae	0.00	1.09	1.20	1.17	2.21	43.86
Chaetozone setosa	1.69	2.23	1.20	1.33	2.21	46.07
Goniada maculata	0.00	1.03	1.13	1.17	2.09	48.16
Euchone papillosa	0.68	1.09	1.13	1.11	2.09	50.25
Heteromastus filiformis	1.36	2.25	1.12	0.99	2.06	52.31
Diastyloides serratus	0.89	0.34	1.05	0.83	1.94	54.24
Rhodine loveni	1.25	1.16	1.05	1.00	1.93	56.17
Thyasira equalis	3.72	2.91	1.00	1.13	1.85	58.02
Diplocirrus glaucus	1.14	0.69	1.00	1.03	1.85	59.87
Hyala vitrea	0.75	0.00	0.79	0.68	1.45	61.31
Galathowenia oculata	0.00	0.69	0.76	0.78	1.39	62.71
Aphelochaeta sp	0.00	0.69	0.73	0.79	1.35	64.06
Amphiura filiformis	0.57	0.34	0.73	0.79	1.34	65.40
Chaetoparia nilssoni	0.57	0.34	0.72	0.79	1.33	66.73
Melinna cristata	0.68	0.00	0.71	0.68	1.31	68.04
Abyssoninoe hibernica	0.57	0.00	0.67	0.68	1.24	69.28
Diastylis boecki	0.57	0.00	0.67	0.68	1.24	70.52
Gnathia spp	0.57	0.00	0.67	0.68	1.24	71.77
Corbula gibba	0.57	0.00	0.60	0.68	1.10	72.87
Ennucula tenuis	0.57	0.00	0.60	0.68	1.10	73.97

Mysella bidentata	0.57	0.00	0.60	0.68	1.10	75.07
Neoamphitrite affinis	0.57	0.00	0.60	0.68	1.10	76.17
Neoamphitrite grayi	0.57	0.00	0.60	0.68	1.10	77.27
Praxillella affinis	0.57	0.00	0.60	0.68	1.10	78.37
Campylaspis costata	0.57	0.00	0.59	0.68	1.09	79.45
Leptostylis longimana	0.57	0.00	0.59	0.68	1.09	80.54
Ophiocten affinis	0.57	0.00	0.59	0.68	1.09	81.62
Phyllodoce rosea	0.57	0.00	0.59	0.68	1.09	82.71
Phylo norvegica	0.57	0.00	0.59	0.68	1.09	83.79
Terebellides stroemi	0.57	0.00	0.59	0.68	1.09	84.88
Thyasira sarsii	0.57	0.00	0.59	0.68	1.09	85.97
Spiophanes kroeyeri	2.87	2.85	0.50	1.60	0.91	86.88
Aphelochaeta marioni	2.06	2.41	0.42	1.39	0.78	87.65
Bylgides elegans	0.00	0.41	0.42	0.48	0.77	88.42
Polydora spp	0.00	0.34	0.40	0.48	0.74	89.16
Scoletoma fragilis	0.00	0.34	0.40	0.48	0.74	89.90
Ceratocephale loveni	2.10	2.14	0.39	1.39	0.73	90.63

Groups Ref-80:1 & Ref-80:14
Average dissimilarity = 50.87%

Species	Group Ref-80:1 Av. Abund	Group Ref-80:14 Av. Abund	Av. Diss	Diss/SD	Contrib. %	Cumul. %
Paramphinome jeffreysii	1.50	3.75	1.58	1.82	3.11	3.11
Scalibregma inflatum	0.00	2.26	1.58	9.00	3.11	6.22
Amphiura filiformis	1.93	0.00	1.35	7.81	2.65	8.87
Ampelisca gibba	1.82	0.00	1.27	15.93	2.49	11.36
Lipobranchius jeffreysii	0.68	2.42	1.24	1.68	2.43	13.79
Brada villosa	0.00	1.69	1.18	1.81	2.33	16.12
Chaetozone setosa	1.14	2.81	1.13	1.73	2.22	18.34
Cerebratulus spp	0.00	1.56	1.07	1.90	2.11	20.46
Ceratocephale loveni	0.75	2.23	1.05	1.51	2.07	22.52
Goniada maculata	0.00	1.44	1.01	1.88	1.99	24.51
Pista cristata	1.50	0.69	0.90	1.30	1.77	26.29
Paramphitrite tetrabranchia	1.71	0.90	0.88	2.00	1.73	28.02
Sosane sulcata	1.14	0.00	0.82	1.36	1.61	29.63
Brissopsis lyrifera	1.38	0.69	0.81	1.16	1.60	31.23
Luidia sarsi	1.14	0.00	0.78	1.36	1.53	32.75
Notomastus latericeus	1.14	0.00	0.78	1.36	1.53	34.28
Praxillella affinis	1.14	0.00	0.78	1.36	1.53	35.81
Ophiodromus flexuosus	0.00	1.09	0.76	1.17	1.49	37.30
Amphiura chiajei	1.32	1.75	0.74	1.09	1.46	38.76
Prionospio cirrifera	0.00	1.03	0.72	1.18	1.42	40.18
Rhodine loveni	1.14	1.69	0.70	1.14	1.38	41.55
Euchone papillosa	1.14	0.75	0.69	1.12	1.35	42.90
Polynoidae	1.14	0.75	0.68	1.12	1.34	44.24
Glycera alba	1.38	2.24	0.66	1.08	1.30	45.54
Abra nitida	2.33	1.62	0.66	1.10	1.29	46.83
Glycera rouxii	0.68	0.75	0.65	0.93	1.28	48.11
Philine scabra	0.57	1.03	0.63	1.03	1.24	49.35
Melinna cristata	0.75	0.34	0.61	0.83	1.19	50.54
Pseudamussium peslutrae	0.57	0.75	0.60	0.94	1.18	51.72
Neoamphitrite affinis	0.57	0.48	0.59	0.84	1.17	52.89
Ennucula tenuis	2.25	1.54	0.59	0.95	1.16	54.04
Aphelochaeta marioni	2.29	3.02	0.58	2.05	1.14	55.18
Galathowenia oculata	0.57	0.45	0.56	0.84	1.10	56.28
Terebellides stroemi	0.57	0.69	0.55	0.90	1.08	57.36
Leucothoe lilljeborgii	0.57	0.41	0.54	0.82	1.06	58.42
Yoldiella philippiana	2.95	2.29	0.54	1.47	1.06	59.47
Heteromastus filiformis	2.13	2.86	0.52	1.85	1.02	60.50
Eclysippe eliasoni	1.25	1.89	0.51	0.92	1.01	61.50
Pectinaria koreni	0.00	0.69	0.50	0.79	0.98	62.48
Maldane sarsi	0.75	0.00	0.50	0.68	0.97	63.46
Chaetoderma nitidulum	0.57	0.34	0.49	0.79	0.97	64.43
Pholoe baltica	0.00	0.69	0.49	0.79	0.97	65.40
Westwoodilla caecula	0.57	0.34	0.49	0.79	0.96	66.36

Nereiphylla lutea	0.57	0.34	0.48	0.79	0.94	67.29
Montacuta tenella	0.68	0.00	0.48	0.68	0.94	68.23
Diastylis boecki	0.00	0.69	0.47	0.79	0.92	69.15
Anobothrus gracilis	0.00	0.69	0.46	0.79	0.90	70.05
Echinocardium flavescens	0.68	0.00	0.45	0.68	0.88	70.93
Gnathia spp	0.68	0.00	0.45	0.68	0.88	71.81
Philine aperta	0.68	0.00	0.45	0.68	0.88	72.69
Prionospio dubia	0.68	0.00	0.45	0.68	0.88	73.57
Glycinde nordmanni	0.57	0.00	0.42	0.68	0.82	74.40
Harpinia antennaria	0.57	0.00	0.42	0.68	0.82	75.22
Phascolion strombus	0.57	0.00	0.42	0.68	0.82	76.04
Spatangidae	0.57	0.00	0.42	0.68	0.82	76.86
Thyasira equalis	3.87	3.27	0.41	1.54	0.81	77.67
Harpinia crenulata	0.57	0.00	0.40	0.68	0.79	78.46
Iphitime hartmanae	0.57	0.00	0.40	0.68	0.79	79.25
Polycirrus spp	0.57	0.00	0.40	0.68	0.79	80.03
Proclea graffii	0.57	0.00	0.40	0.68	0.79	80.82
Campylaspis costata	0.57	0.00	0.38	0.68	0.74	81.56
Cuspidaria spp	0.57	0.00	0.38	0.68	0.74	82.30
Gattyana amondseni	0.57	0.00	0.38	0.68	0.74	83.04
Glyphohesione klatti	0.57	0.00	0.38	0.68	0.74	83.78
Hyala vitrea	0.57	0.00	0.38	0.68	0.74	84.52
Lysianassidae	0.57	0.00	0.38	0.68	0.74	85.26
Nemertea	0.57	0.00	0.38	0.68	0.74	86.00
Pectinaria belgica	0.57	0.00	0.38	0.68	0.74	86.74
Phyllodoce rosea	0.57	0.00	0.38	0.68	0.74	87.48
Scoletoma fragilis	0.57	0.00	0.38	0.68	0.74	88.22
Tharyx killariensis	0.57	0.00	0.38	0.68	0.74	88.96
Spiophanes kroeyeri	3.38	3.17	0.36	1.15	0.71	89.67
Prionospio fallax	0.00	0.48	0.34	0.48	0.66	90.33

Groups AC+clay-95:14 & Ref-80:14
Average dissimilarity = 51.09%

Species	Group AC+clay-95:14 Av. Abund	Group Ref-80:14 Av. Abund	Av. Diss	Diss/SD	Contrib. %	Cumul. %
Streblosoma bairdi	0.00	2.63	2.23	9.80	4.37	4.37
Lipobranchius jeffreysii	0.00	2.42	2.05	13.47	4.02	8.38
Abyssoninoe hibernica	0.00	2.31	1.97	8.09	3.85	12.24
Scalibregma inflatum	0.34	2.26	1.65	2.55	3.23	15.46
Eclysippe eliasoni	0.00	1.89	1.61	7.55	3.14	18.61
Brada villosa	0.00	1.69	1.44	1.84	2.83	21.43
Eudorella emarginata	1.65	0.00	1.42	1.89	2.78	24.22
Leucon nasica	1.56	0.00	1.35	1.92	2.64	26.86
Arrhis phyllonyx	1.78	0.41	1.34	1.52	2.62	29.47
Amphiura chiajei	0.34	1.75	1.32	1.59	2.58	32.05
Ennucula tenuis	0.00	1.54	1.29	1.90	2.52	34.57
Abra nitida	2.95	1.62	1.17	1.49	2.29	36.86
Paramphinome jeffreysii	3.22	3.75	1.17	1.02	2.29	39.15
Cerebratulus spp	0.34	1.56	1.14	1.54	2.23	41.37
Diplocirrus glaucus	0.69	1.99	1.13	1.37	2.20	43.58
Philine scabra	2.07	1.03	0.91	1.12	1.78	45.36
Rhodine loveni	1.16	1.69	0.89	1.08	1.74	47.10
Prionospio cirrifera	0.00	1.03	0.88	1.19	1.72	48.83
Ophiodromus flexuosus	0.34	1.09	0.87	1.13	1.70	50.52
Polynoidae	1.09	0.75	0.85	1.09	1.67	52.19
Euchone papillosa	1.09	0.75	0.85	1.09	1.66	53.85
Paramphitrite tetrabranchia	0.00	0.90	0.73	0.80	1.43	55.28
Galathowenia oculata	0.69	0.45	0.73	0.93	1.43	56.71
Yoldiella philippiana	1.72	2.29	0.72	1.03	1.41	58.12
Glycera rouxii	0.34	0.75	0.70	0.87	1.38	59.50
Goniada maculata	1.03	1.44	0.69	0.94	1.35	60.85
Chaetozone setosa	2.23	2.81	0.67	2.05	1.31	62.16
Thyasira equalis	2.91	3.27	0.65	1.34	1.27	63.43
Aphelochaeta marioni	2.41	3.02	0.65	2.25	1.27	64.70
Pista cristata	0.34	0.69	0.64	0.87	1.25	65.95

Brissopsis lyrifera	0.34	0.69	0.63	0.87	1.24	67.18
Pseudamussium peslutrae	0.00	0.75	0.62	0.80	1.21	68.39
Pectinaria koreni	0.00	0.69	0.61	0.80	1.20	69.59
Pholoe baltica	0.00	0.69	0.60	0.80	1.18	70.78
Aphelochaeta sp	0.69	0.00	0.58	0.80	1.13	71.90
Diastylis boecki	0.00	0.69	0.57	0.80	1.11	73.01
Terebellides stroemi	0.00	0.69	0.57	0.80	1.11	74.12
Anobothrus gracilis	0.00	0.69	0.56	0.80	1.09	75.21
Spiophanes kroeyeri	2.85	3.17	0.55	1.31	1.08	76.29
Heteromastus filiformis	2.25	2.86	0.53	1.68	1.04	77.33
Bylgides elegans	0.41	0.34	0.53	0.69	1.03	78.36
Polydora spp	0.34	0.34	0.47	0.67	0.92	79.28
Turbellaria	0.34	0.34	0.46	0.67	0.91	80.19
Nudibranchia	0.34	0.34	0.46	0.67	0.90	81.09
Harmothoe spp	0.34	0.34	0.46	0.67	0.89	81.98
Diastyloides serratus	0.34	0.34	0.45	0.67	0.88	82.87
Neoamphitrite affinis	0.00	0.48	0.42	0.49	0.83	83.69
Ampelisca macrocephala	0.00	0.45	0.41	0.49	0.81	84.51
Prionospio fallax	0.00	0.48	0.41	0.49	0.80	85.30
Eriopisa elongata	0.00	0.41	0.37	0.49	0.73	86.04
Leucothoe lilljeborgii	0.00	0.41	0.36	0.49	0.70	86.73
Aora gracilis	0.00	0.41	0.33	0.49	0.65	87.38
Chaetoderma nitidulum	0.00	0.34	0.31	0.49	0.62	88.00
Scoletoma fragilis	0.34	0.00	0.31	0.49	0.60	88.61
Diastyloides biplicatus	0.00	0.34	0.30	0.49	0.59	89.19
Eumida bahusiensis	0.00	0.34	0.30	0.49	0.59	89.78
Nephtys incisa	0.34	0.00	0.30	0.49	0.58	90.35

Groups AC+clay-95:14 & Ref-95:14
Average dissimilarity = 41.86%

Species	Group AC+clay-95:14 Av. Abund	Group Ref-95:14 Av. Abund	Av. Diss	Diss/SD	Contrib. %	Cumul. %
Scalibregma inflatum	0.34	2.43	2.00	2.47	4.78	4.78
Prionospio cirrifera	0.00	1.86	1.75	1.87	4.18	8.97
Chaetozone setosa	2.23	4.02	1.70	4.23	4.06	13.03
Paramphinome jeffreysii	3.22	3.08	1.47	1.40	3.50	16.53
Melinna cristata	0.00	1.37	1.28	1.95	3.06	19.59
Cerebratulus spp	0.34	1.20	1.09	1.14	2.61	22.20
Yoldiella philippiana	1.72	1.23	1.02	1.13	2.44	24.64
Eriopisa elongata	0.00	1.09	1.00	1.19	2.39	27.03
Euchone papillosa	1.09	0.34	0.97	1.12	2.33	29.35
Philine scabra	2.07	1.20	0.96	1.10	2.30	31.66
Polynoidae	1.09	0.75	0.95	1.09	2.27	33.92
Glycera alba	2.09	1.31	0.95	1.00	2.26	36.18
Rhodine loveni	1.16	1.16	0.93	1.03	2.23	38.41
Galathowenia oculata	0.69	0.83	0.91	1.01	2.17	40.58
Glycera rouxii	0.34	1.03	0.90	1.10	2.15	42.73
Thyasira equalis	2.91	3.00	0.89	1.26	2.13	44.86
Eudorella emarginata	1.65	1.80	0.85	0.94	2.02	46.88
Goniada maculata	1.03	1.91	0.83	1.01	1.98	48.87
Aphelochaeta marioni	2.41	3.27	0.81	3.45	1.94	50.80
Bylgides elegans	0.41	0.75	0.80	0.89	1.91	52.71
Diastyloides serratus	0.34	0.75	0.78	0.88	1.87	54.58
Arrhis phyllonyx	1.78	2.21	0.75	1.00	1.79	56.37
Pectinaria koreni	0.00	0.75	0.72	0.80	1.72	58.09
Terebellides stroemi	0.00	0.75	0.71	0.79	1.71	59.80
Diplocirrus glaucus	0.69	0.34	0.71	0.87	1.70	61.50
Leucon nasica	1.56	2.33	0.71	0.95	1.70	63.20
Sige fusigera	0.00	0.75	0.68	0.80	1.64	64.84
Abra nitida	2.95	2.41	0.66	1.73	1.59	66.43
Aphelochaeta sp	0.69	0.00	0.64	0.80	1.54	67.96
Heteromastus filiformis	2.25	2.81	0.64	1.28	1.52	69.49
Spiophanes kroeyeri	2.85	3.26	0.63	1.18	1.50	70.98
Pista cristata	0.34	0.45	0.61	0.70	1.45	72.43
Lysianassidae	0.34	0.34	0.53	0.67	1.27	73.70

Ceratocephale loveni	2.14	2.57	0.53	1.30	1.26	74.96
Polydora spp	0.34	0.34	0.53	0.67	1.26	76.22
Chaetoparia nilssoni	0.34	0.34	0.52	0.67	1.24	77.46
Brissopsis lyrifera	0.34	0.34	0.51	0.67	1.22	78.68
Ophiuridae	0.00	0.41	0.42	0.49	1.00	79.68
Streblosoma bairdi	0.00	0.41	0.36	0.49	0.86	80.54
Anobothrus gracilis	0.00	0.34	0.35	0.49	0.84	81.38
Lipobranchius jeffreysii	0.00	0.34	0.35	0.49	0.84	82.21
Ophelina norvegica	0.00	0.34	0.35	0.49	0.84	83.05
Phoxocephalidae	0.00	0.34	0.35	0.49	0.84	83.89
Scoletoma fragilis	0.34	0.00	0.35	0.49	0.83	84.72
Ennucula tenuis	0.00	0.34	0.33	0.49	0.80	85.52
Westwoodilla caecula	0.00	0.34	0.33	0.49	0.80	86.32
Nephtys incisa	0.34	0.00	0.33	0.49	0.79	87.11
Amphiura chiajei	0.34	0.00	0.33	0.49	0.79	87.89
Amphiura filiformis	0.34	0.00	0.33	0.49	0.79	88.68
Parvicardium pinnulatum	0.34	0.00	0.33	0.49	0.79	89.47
Ophiodromus flexuosus	0.34	0.00	0.33	0.49	0.79	90.25

Groups Ref-80:14 & Ref-95:14
Average dissimilarity = 46.68%

Species	Group Ref-80:14 Av. Abund	Group Ref-95:14 Av. Abund	Av. Diss	Diss/SD	Contrib. %	Cumul. %
Leucon nasica	0.00	2.33	1.79	9.88	3.83	3.83
Abyssoninoe hibernica	2.31	0.00	1.77	8.07	3.79	7.62
Streblosoma bairdi	2.63	0.41	1.72	2.47	3.67	11.30
Lipobranchius jeffreysii	2.42	0.34	1.57	2.95	3.36	14.66
Eclysippe eliasoni	1.89	0.00	1.44	7.60	3.09	17.75
Arrhis phyllonyx	0.41	2.21	1.42	2.16	3.04	20.79
Eudorella emarginata	0.00	1.80	1.36	1.86	2.92	23.71
Amphiura chiajei	1.75	0.00	1.35	1.88	2.89	26.59
Brada villosa	1.69	0.00	1.30	1.84	2.78	29.38
Diplocirrus glaucus	1.99	0.34	1.27	2.02	2.72	32.10
Ennucula tenuis	1.54	0.34	1.01	1.51	2.16	34.26
Prionospio cirrifera	1.03	1.86	0.95	1.37	2.02	36.28
Chaetozone setosa	2.81	4.02	0.94	1.74	2.01	38.29
Yoldiella philippiana	2.29	1.23	0.91	1.27	1.96	40.25
Melinna cristata	0.34	1.37	0.88	1.42	1.89	42.14
Ophiodromus flexuosus	1.09	0.00	0.83	1.19	1.78	43.92
Rhodine loveni	1.69	1.16	0.80	1.09	1.72	45.64
Eriopisa elongata	0.41	1.09	0.80	1.15	1.71	47.35
Philine scabra	1.03	1.20	0.76	1.12	1.63	48.98
Glycera alba	2.24	1.31	0.76	0.90	1.63	50.61
Cerebratulus spp	1.56	1.20	0.74	1.04	1.58	52.20
Glycera rouxii	0.75	1.03	0.73	1.09	1.57	53.76
Galathowenia oculata	0.45	0.83	0.72	0.89	1.53	55.30
Polynoidae	0.75	0.75	0.70	0.98	1.51	56.81
Pectinaria koreni	0.69	0.75	0.69	0.99	1.47	58.27
Terebellides stroemi	0.69	0.75	0.68	0.98	1.45	59.72
Paramphinome jeffreysii	3.75	3.08	0.67	1.23	1.43	61.15
Paramphitrite tetrabranchia	0.90	0.00	0.66	0.80	1.42	62.57
Abra nitida	1.62	2.41	0.65	1.01	1.40	63.97
Pista cristata	0.69	0.45	0.65	0.93	1.39	65.36
Diastyloides serratus	0.34	0.75	0.63	0.88	1.35	66.71
Bylgides elegans	0.34	0.75	0.62	0.89	1.34	68.05
Euchone papillosa	0.75	0.34	0.62	0.88	1.32	69.37
Pseudamussium peslutrae	0.75	0.34	0.61	0.88	1.31	70.68
Thyasira equalis	3.27	3.00	0.60	1.34	1.29	71.97
Anobothrus gracilis	0.69	0.34	0.57	0.86	1.22	73.19
Brissopsis lyrifera	0.69	0.34	0.57	0.87	1.21	74.40
Sige fusigera	0.00	0.75	0.56	0.80	1.19	75.59
Pholoe baltica	0.69	0.00	0.54	0.80	1.16	76.75
Diastylis boecki	0.69	0.00	0.51	0.80	1.10	77.85
Leucothoe lilljeborgii	0.41	0.34	0.47	0.69	1.02	78.86
Spiophanes kroeyeri	3.17	3.26	0.47	1.35	1.00	79.86

Ophelina norvegica	0.34	0.34	0.42	0.67	0.91	80.77
Westwoodilla caecula	0.34	0.34	0.42	0.67	0.89	81.66
Callianassa subterranea	0.34	0.34	0.41	0.67	0.88	82.55
Polydora spp	0.34	0.34	0.41	0.67	0.88	83.42
Goniada maculata	1.44	1.91	0.39	0.74	0.84	84.26
Neoamphitrite affinis	0.48	0.00	0.38	0.49	0.81	85.08
Ceratocephale loveni	2.23	2.57	0.37	1.25	0.79	85.87
Ampelisca macrocephala	0.45	0.00	0.37	0.49	0.79	86.66
Prionospio fallax	0.48	0.00	0.37	0.49	0.79	87.45
Aphelochaeta marioni	3.02	3.27	0.36	1.07	0.77	88.22
Heteromastus filiformis	2.86	2.81	0.35	1.63	0.74	88.96
Ophiuridae	0.00	0.41	0.33	0.49	0.71	89.67
Scalibregma inflatum	2.26	2.43	0.31	1.50	0.66	90.33
